# Ultrasensitive plasma-based monitoring of tumor burden using machine learning-guided signal enrichment

**DOI:** 10.1038/s41591-024-03040-4

**Published:** 2024-06-14

**Authors:** Adam J. Widman, Minita Shah, Amanda Frydendahl, Daniel Halmos, Cole C. Khamnei, Nadia Øgaard, Srinivas Rajagopalan, Anushri Arora, Aditya Deshpande, William F. Hooper, Jean Quentin, Jake Bass, Mingxuan Zhang, Theophile Langanay, Laura Andersen, Zoe Steinsnyder, Will Liao, Mads Heilskov Rasmussen, Tenna Vesterman Henriksen, Sarah Østrup Jensen, Jesper Nors, Christina Therkildsen, Jesus Sotelo, Ryan Brand, Joshua S. Schiffman, Ronak H. Shah, Alexandre Pellan Cheng, Colleen Maher, Lavinia Spain, Kate Krause, Dennie T. Frederick, Wendie den Brok, Caroline Lohrisch, Tamara Shenkier, Christine Simmons, Diego Villa, Andrew J. Mungall, Richard Moore, Elena Zaikova, Viviana Cerda, Esther Kong, Daniel Lai, Murtaza S. Malbari, Melissa Marton, Dina Manaa, Lara Winterkorn, Karen Gelmon, Margaret K. Callahan, Genevieve Boland, Catherine Potenski, Jedd D. Wolchok, Ashish Saxena, Samra Turajlic, Marcin Imielinski, Michael F. Berger, Sam Aparicio, Nasser K. Altorki, Michael A. Postow, Nicolas Robine, Claus Lindbjerg Andersen, Dan A. Landau

**Affiliations:** 1 New York Genome Center, New York, NY, USA; 2 Memorial Sloan Kettering Cancer Center, New York, NY, USA; 3 Department of Molecular Medicine, Aarhus University Hospital, Aarhus, Denmark; 4 Department of Clinical Medicine, Aarhus University, Aarhus, Denmark; 5 Weill Cornell Medicine, New York, NY, USA; 6 Gastro Unit, Copenhagen University Hospital, Amager – Hvidovre Hospital, Hvidovre, Denmark; 7 Parker Institute for Cancer Immunotherapy, San Francisco, CA, USA; 8 Cancer Dynamics Laboratory, The Francis Crick Institute, London NW1 1AT, UK; 9 Renal and Skin Unit, The Royal Marsden NHS Foundation Trust, London SW3 6JJ, UK; 10 Mass General Cancer Center, Massachusetts General Hospital, Boston, MA, USA; 11 Department of Medical Oncology, BC Cancer Agency, Vancouver, Canada; 12 Michael Smith Genome Sciences Centre, Vancouver, Canada; 13 Department of Molecular Oncology, BC Cancer Agency, Vancouver, Canada; 14 Department of Pathology and Laboratory Medicine, University of British Columbia, Vancouver, Canada

**Keywords:** Machine learning, Minimal residual disease, Circulating tumor DNA, cell free DNA, Whole genome sequencing, Cancer monitoring, Immunotherapy

## Abstract

In solid tumor oncology, circulating tumor DNA (ctDNA) is poised to transform care through accurate assessment of minimal residual disease (MRD) and therapeutic response monitoring. To overcome the sparsity of ctDNA fragments in low tumor fraction (TF) settings and increase MRD sensitivity, we previously leveraged genome-wide mutational integration through plasma whole genome sequencing (WGS). We now introduce MRD-EDGE, a machine learning-guided WGS ctDNA single nucleotide variant (SNV) and copy number variant (CNV) detection platform designed to increase signal enrichment. MRD-EDGE^SNV^ uses deep learning and a ctDNA-specific feature space to increase SNV signal-to-noise enrichment in WGS by ~300X compared to previous WGS error suppression. MRD-EDGE^CNV^ also reduces the degree of aneuploidy needed for ultrasensitive CNV detection through WGS from 1 Gb to 200 Mb, vastly expanding its applicability within solid tumors. We harness the improved performance to identify MRD following surgery in multiple cancer types, track changes in TF in response to neoadjuvant immunotherapy in lung cancer and demonstrate ctDNA shedding in precancerous colorectal adenomas. Finally, the radical signal-to-noise enrichment in MRD-EDGE^SNV^ enables plasma-only (non tumor-informed) disease monitoring in advanced melanoma and lung cancer, yielding clinically informative TF monitoring for patients on immune checkpoint inhibition (ICI).

## Introduction

Liquid biopsy offers to reshape cancer care through the noninvasive detection and monitoring of plasma circulating tumor DNA (ctDNA). Recent efforts have focused on extending the use of liquid biopsy to low tumor fraction (TF) settings including therapeutic response monitoring or minimal residual disease (MRD) assessment. To overcome ctDNA sparsity in these settings, many have employed deep targeted sequencing to capture mutations from tumor-informed bespoke panels^1–3^ or common cancer driver genes^4–7^. However, missed detections are prevalent in current assays. For example, MRD identified via bespoke panels in urothelial carcinoma is strongly prognostic of disease recurrence, though ~40% of ctDNA-negative patients experienced relapse^1^. Similar false negatives were seen in breast^4,8^ and colorectal cancer^3,9,10^, suggesting that further improvement in sensitivity is needed.

Sensitivity barriers in deep targeted panels arise from the limited number of ctDNA fragments recovered at targeted loci^11–14^. Even with ultra-deep sequencing, a somatic mutation cannot be observed if it is not sampled in the limited plasma volume collected in routine testing, which imposes a hard barrier on effective coverage depth. Targeted approaches seek to overcome this limitation by increasing the number of panel-covered mutations to dozens^1,2,6,7,15^ or hundreds^11,16,17^.

We previously proposed an alternative approach where sequencing breadth supplants sequencing depth via integration of thousands of single nucleotide variants (SNVs) and copy number variants (CNVs) across the cancer genome through plasma whole genome sequencing (WGS)^14^. We designed a support vector machine approach, MRDetect, to suppress low quality cfDNA SNV artifacts derived from WGS errors. Building on this work, we reasoned that learning patterns specific to ctDNA mutagenesis can offer *signal enrichment* to complement *suppression of sequencing error*. We developed MRD-EDGE (**E**nhanced ct**D**NA **G**enome-wide signal **E**nrichment), which integrates complementary signal from SNVs (MRD-EDGE^SNV^) and CNVs (MRD-EDGE^CNV^) to increase ctDNA signal enrichment in plasma WGS. We demonstrate the clinical utility of this approach in critical low TF settings.

## Results

### Deep learning distinguishes ctDNA SNVs from sequencing error

**Extended Data Fig. 1:**
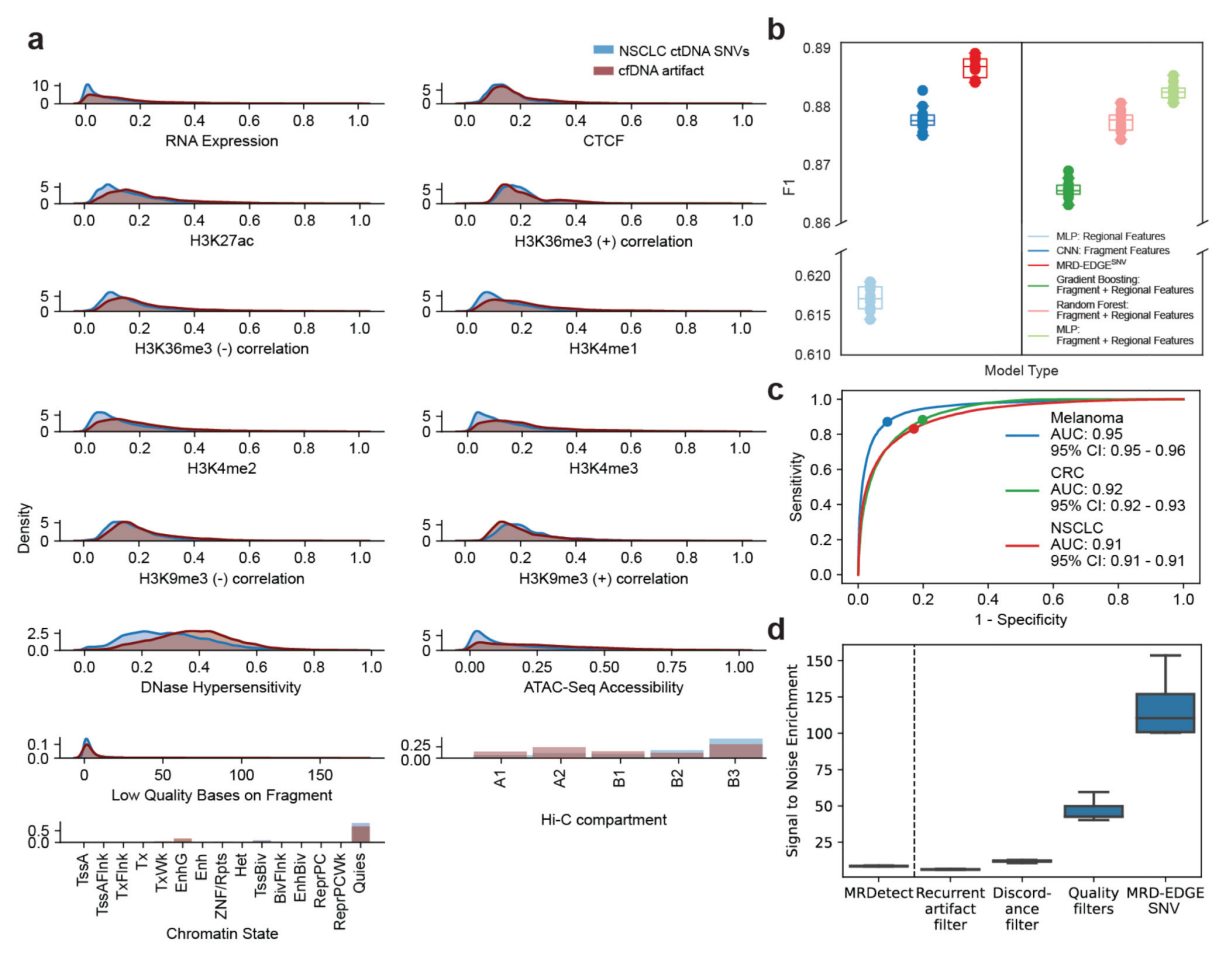
MRD-EDGE^SNV^ feature selection, model architecture and performance **a)** Feature density plots for ctDNA and cfDNA SNV artifacts used in the MRD-EDGE^SNV^ NSCLC model. These fragments were subject to quality filters (Supplementary Table 2) to remove low quality SNV artifacts prior to this analysis. In this comparison, ctDNA SNV fragments are identified from consensus mutation calls in high-burden NSCLC plasma samples (Supplementary Table 1) and compared to cfDNA SNV fragments (sequencing errors) drawn from within the same plasma sample to preclude sample-specific biases when establishing predictive ability of individual features. **b)** SNV classification performance for different machine learning models. F1 score was assessed on tumor-confirmed melanoma ctDNA SNV fragments vs. cfDNA artifacts from healthy controls. Random subsamplings were drawn from the held-out melanoma validation set (Supplementary Table 1), which was split into tenths for this analysis. We compared performance between MRD-EDGE^SNV^ and its separate components (left), as well as to other ML architectures (right) **c)** Fragment-level ROC analysis for MRD-EDGE^SNV^ classifier for different cancer types. Performance is assessed on filtered fragments (~90% of low-quality cfDNA artifacts are excluded by quality filters) in held-out validation sets (Supplementary Table 1) for melanoma (blue), CRC (green), and NSCLC (red). Colored dots on curves indicate the tumor-informed decision threshold (0.5) used in each tumor type to classify individual SNV fragments as ctDNA or cfDNA artifact. **d)** Signal-to-noise enrichment analysis for MRDetect and for each step of the MRD-EDGE^SNV^ tumor-informed pipeline. Final pipeline enrichment is 118-fold for MRD-EDGE^SNV^ vs. 8.3-fold for MRDetect^SNV^ in the same datasets.

**Figure 1:**
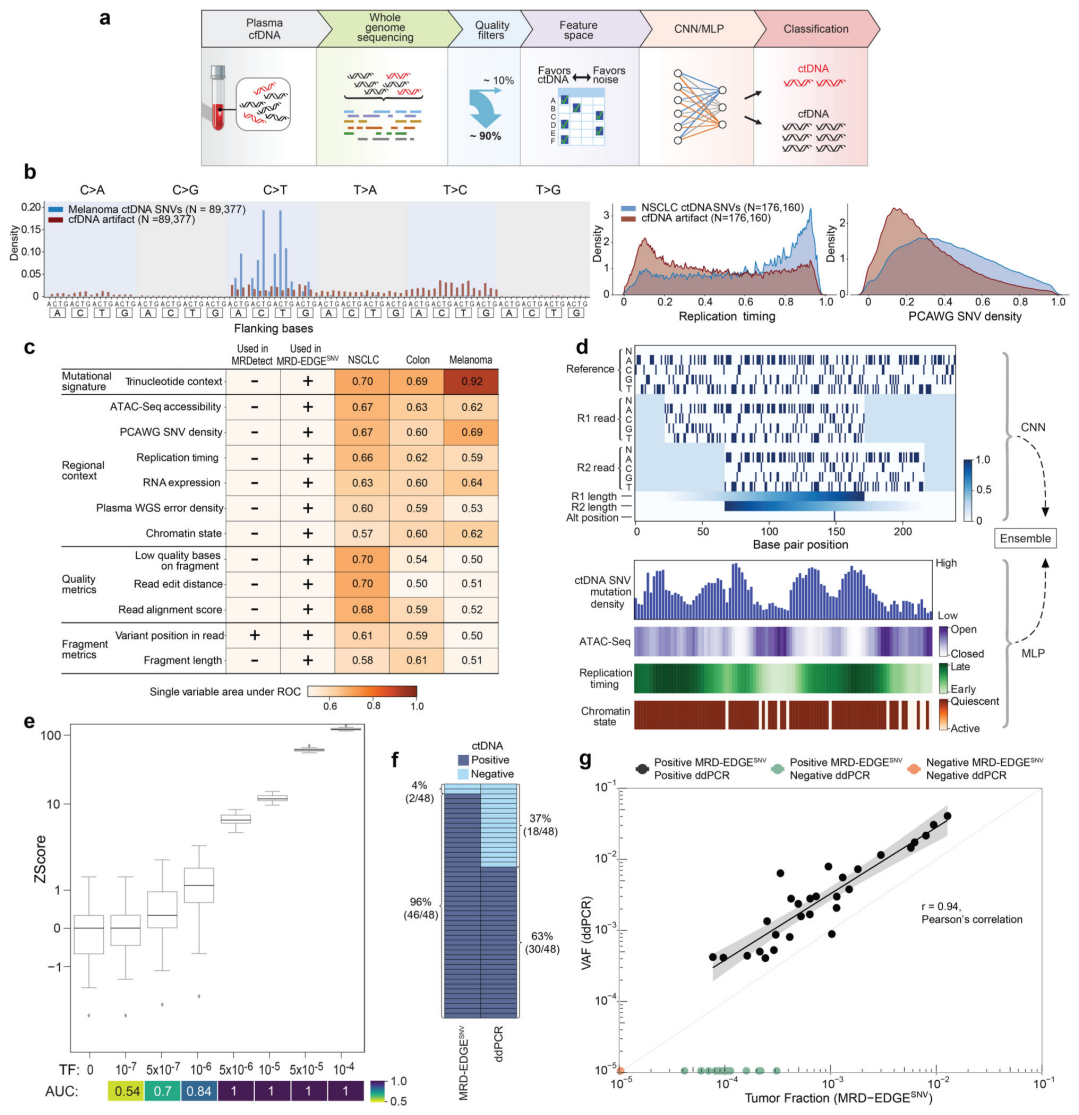
MRD-EDGE^SNV^ deep learning classifier distinguishes ctDNA SNV fragments from cfDNA artifacts **a)** MRD-EDGE schematic. **b)** Selected feature density plots for ctDNA and cfDNA SNV artifacts: trinucleotide context (left), replication timing (middle)^25^, PCAWG^60^ (right). **c)** Heatmap of predictive power of selected features (Methods) measured by single variable area under the receiver operating curve (svAUC, Methods) in NSCLC, CRC, and melanoma. Feature use in MRDetect or MRD-EDGE^SNV^ is indicated. **d)** (top) Illustration of the fragment tensor, an 18x240 matrix encoding of the reference sequence, R1 and R2 read pairs, R1 and R2 read length, and SNV position in the fragment (‘Alt position’). The fragment tensor is passed as input to a convolutional neural network (CNN). (bottom) Relationship between local ctDNA SNV mutation density at the chromosome level and regional features: cancer type-specific chromatin inaccessibility (ATAC-Seq), late replicating regions (Replication timing) and quiescent genomic regions (Chromatin state) are associated with increased density of tumor-confirmed ctDNA SNVs. Regional features (Supplementary Table 2) are encoded as tabular values and passed as input to a multilayer perceptron (MLP). An ensemble classifier takes input from both the fragment and regional models to determine the likelihood that each fragment is ctDNA or cfDNA SNV artifact. **e)** *In silico* studies of cfDNA from the metastatic cutaneous melanoma sample MEL-100 mixed into cfDNA from a healthy plasma sample (CTRL-216) at mix fractions TF = 10^-7^–10^-4^ at 16X coverage depth, performed in 20 technical replicates with independent sampling seeds. An AUC heatmap demonstrates detection performance at the different admixed TFs vs. negative controls (TF=0) as measured by Z score, with tumor-informed MRD-EDGE^SNV^ enabling sensitive detection at TF=5*10^-7^ (AUC 0.70). Box plots represent median, lower and upper quartiles; whiskers correspond to 1.5 x interquartile range. **f)** ctDNA detection status of preoperative stage III CRC plasma samples analyzed by MRD-EDGE^SNV^ and ddPCR (n = 48). **g)** Comparison of estimated ctDNA levels estimated by MRD-EDGE^SNV^ (TFs) and ddPCR (variant allele frequency, VAF). Estimated TFs/VAFs of ctDNA-negative samples were set to 0. Linear regression includes samples called positive by both ddPCR and MRD-EDGE^SNV^ (black dots). Shaded area represents 95% confidence interval.

Our previous error suppression framework (“MRDetect^SNV^”) focused on using quality metrics to eliminate WGS sequencing error. We posited that signal-to-noise enrichment may emerge not only from characterizing features specific to sequencing errors (decreasing noise), but also from learning features indicative of true ctDNA mutations (increasing signal). To do so, we developed a new model training paradigm in which we compared SNV fragments (true label) from plasma samples with high TF (range 8-37%, Supplementary Table 1) to SNV fragments containing sequencing errors drawn from controls without known cancer. First, we implemented a set of quality filters to remove low quality SNV artifacts prior to classification, eliminating ~90% cfDNA artifacts (Supplementary Table 2). We next explored a feature space to help distinguish true ctDNA SNVs from cfDNA sequencing error variants. We evaluated features such as single base substitutions (SBS) sequence patterns^18,19^, cfDNA fragment size^20–22^, and regional predilection for somatic mutagenesis^23–28^ across cancer types (Fig. 1a-c, Extended Data Fig. 1a, Supplementary Table 2).

To integrate this expanded feature set for optimal classification, we developed a two-dimensional convolutional neural network representation of a cfDNA fragment (fragment CNN; Fig. 1d) to capture fragment-level features such as SBS, fragment length, and quality metrics like read edit distance and position in read (PIR). In parallel, a second multilayer perceptron model was designed whereby each SNV-containing fragment is classified based on salient regional features (e.g., replication timing) associated with mutation frequency (regional MLP; Fig. 1d). We combined our fragment and regional models as inputs to an ensemble model, which outperformed each of the models individually as well as other machine learning architectures (Extended Data Fig. 1b, Supplementary Table 3). Our classification yielded high performance in distinguishing true ctDNA SNVs from sequencing artifacts across 3 common cancer types with high mutational burden: melanoma, non-small cell lung cancer (NSCLC), and colorectal cancer (CRC) (Extended Data Fig. 1c, Supplementary Table 1, interpretability assessments Supplementary Fig. 1).

**Extended Data Fig. 2:**
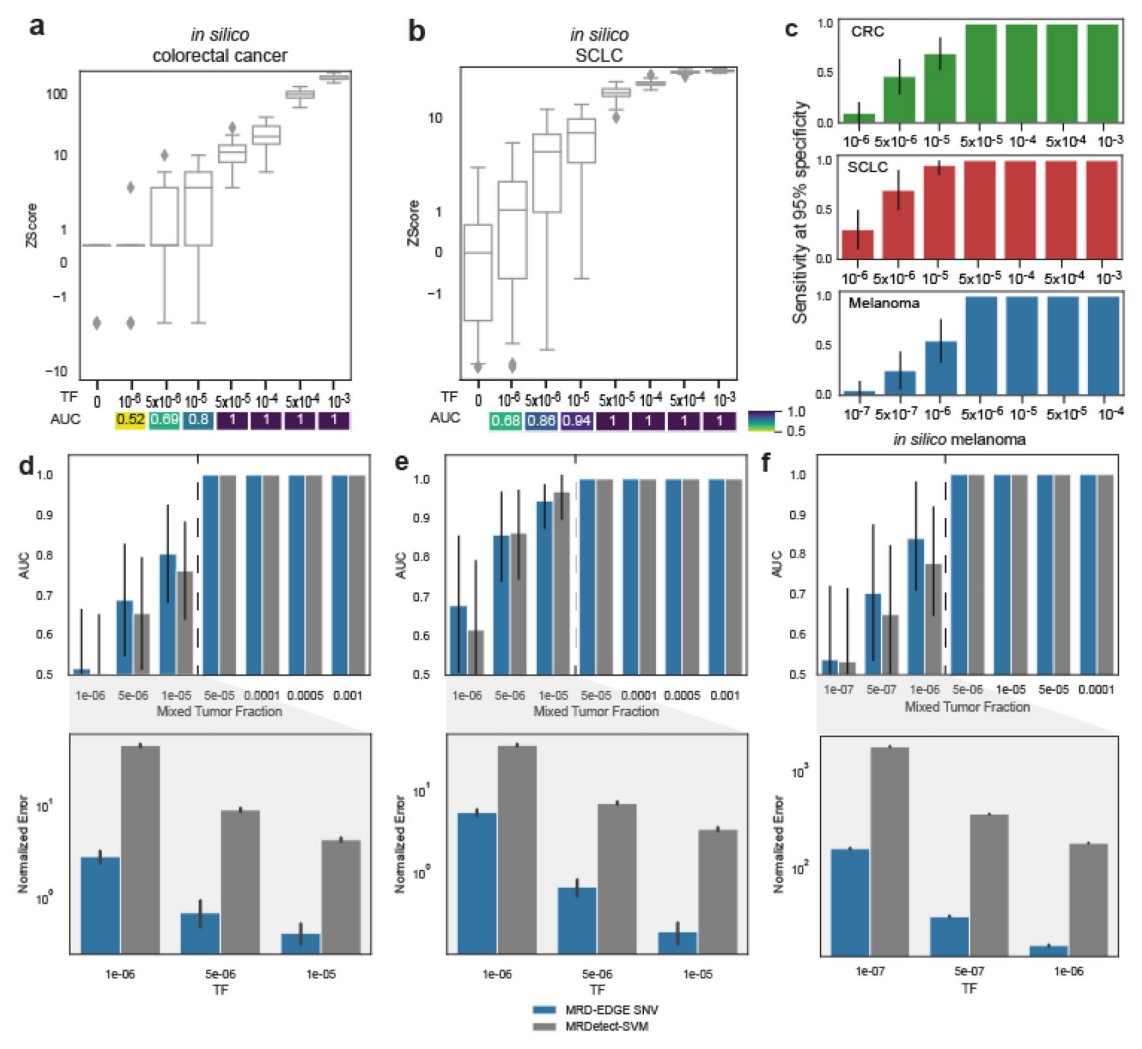
Lower limit of detection studies with MRD-EDGE^SNV^ **a)** *In silico* studies of cfDNA from the metastatic colorectal cancer sample CRC-863 mixed into cfDNA from a healthy plasma sample (CTRL-335) at mixing fractions TF = 10^-6^–10^-3^ at 29X coverage depth, performed in 30 technical replicates with independent sampling seeds. Tumor-informed MRD-EDGE^SNV^ enables sensitive detection of TF as low as 1*10^-5^ (AUC 0.80), measured by Z score of SNV detection rates against unmixed control plasma (TF=0, n=30 randomly chosen replicates). **b)** *In silico* studies of cfDNA from the metastatic small cell lung cancer sample SC-128_0w mixed into cfDNA from a healthy plasma sample (CTRL-216) at mixing fractions TF = 10^-6^–10^-3^ at 25X coverage depth, performed in 20 technical replicates with independent sampling seeds. Tumor-informed MRD-EDGE^SNV^ enables sensitive detection of TF as low as 5*10^-6^ (AUC 0.86), measured by Z score of SNV detection rates against unmixed control plasma (TF=0, *n*=20 randomly chosen replicates). Box plots represent median, lower and upper quartiles; whiskers correspond to 1.5 x interquartile range. An AUC heatmap measures detection vs. TF=0 at different mixed TFs. **c)** Sensitivity at 95% specificity for tumor-informed MRD-EDGE^SNV^ *in silico* studies in green) CRC, red) SCLC, and blue) melanoma. Mixed TF replicates were compared to TF=0 replicates by sample-level MRD-EDGE^SNV^ Z score. **d-f)** Detection performance vs. TF=0 at different mixed TFs for MRD-EDGE^SNV^ (blue) and MRDetect^SNV^ SVM (gray). The AUC is measured by a sample Z score (positive label) compared to TF=0 distribution (negative label) for each replicate at each TF. Error bars represent 95% CI (DeLong AUC variance). (bottom) Normalized error for a subset of mixed TFs between MRD-EDGE^SNV^ and MRDetect^SNV^. Error bars represent 95% CI. Normalized error is shown for TFs where AUC is less than 1 and is measured as (TF_estimated_-TF_mixed_)/TF_mixed_. **d)** *in silico* CRC studies as defined in **(a), e)** in silico SCLC studies as defined in **(b), f)** *In silico* studies of cfDNA from the metastatic cutaneous melanoma sample MEL-100 mixed into cfDNA from a healthy plasma sample (CTRL-216) at mixing fractions TF = 10^-7^–10^-4^ at 16X coverage depth, performed in 20 technical replicates with independent sampling seeds.

We evaluated MRD-EDGE^SNV^ performance in the tumor-informed setting, where SNVs observed in matched tumor WGS are evaluated in patient-matched vs. control (non-cancer) plasma WGS. Here, we determine plasma ctDNA content by comparing the number of cfDNA fragments that match known tumor SNVs against a background rate of ‘noise’ (observed tumor SNV fragments in non-cancer plasma, Methods)^14^. Tumor-informed MRD-EDGE^SNV^ produced higher signal-to-noise enrichment (mean 118-fold) compared to MRDetect^SNV^ (mean 8.3-fold, Extended Data Fig. 1d). We next evaluated the lower limit of detection (LLOD) for MRD-EDGE^SNV^ with *in silico* TF admixtures (melanoma plasma WGS mixed into plasma WGS from an individual with no known cancer; see Methods for *in silico* admixtures detail; Fig. 1e, Supplementary Table 4). Detection sensitivity was at the parts per million range (AUC of 0.84 for discriminating TF = 1*10^-6^ samples from samples with TF = 0 as controls), with further effective discrimination between different TFs (Supplementary Fig. 2). To confirm sensitivity in other high mutational burden solid tumors, we evaluated MRD-EDGE^SNV^ LLOD in CRC and SCLC in *in silico* TF admixtures and found strong performance at low TFs (AUC of 0.80 at 1*10^-5^ in CRC, 0.86 at 5*10^-6^ in SCLC; Extended Data Fig. 2a-b, clinical detection sensitivity Extended Data Fig. 2c). In each mixing study, MRD-EDGE^SNV^ trended towards improved LLOD performance compared to MRDetect^SNV^ and provided more accurate TF resolution at low TFs (Extended Data Fig. 2d-f).

**Extended Data Fig. 3:**
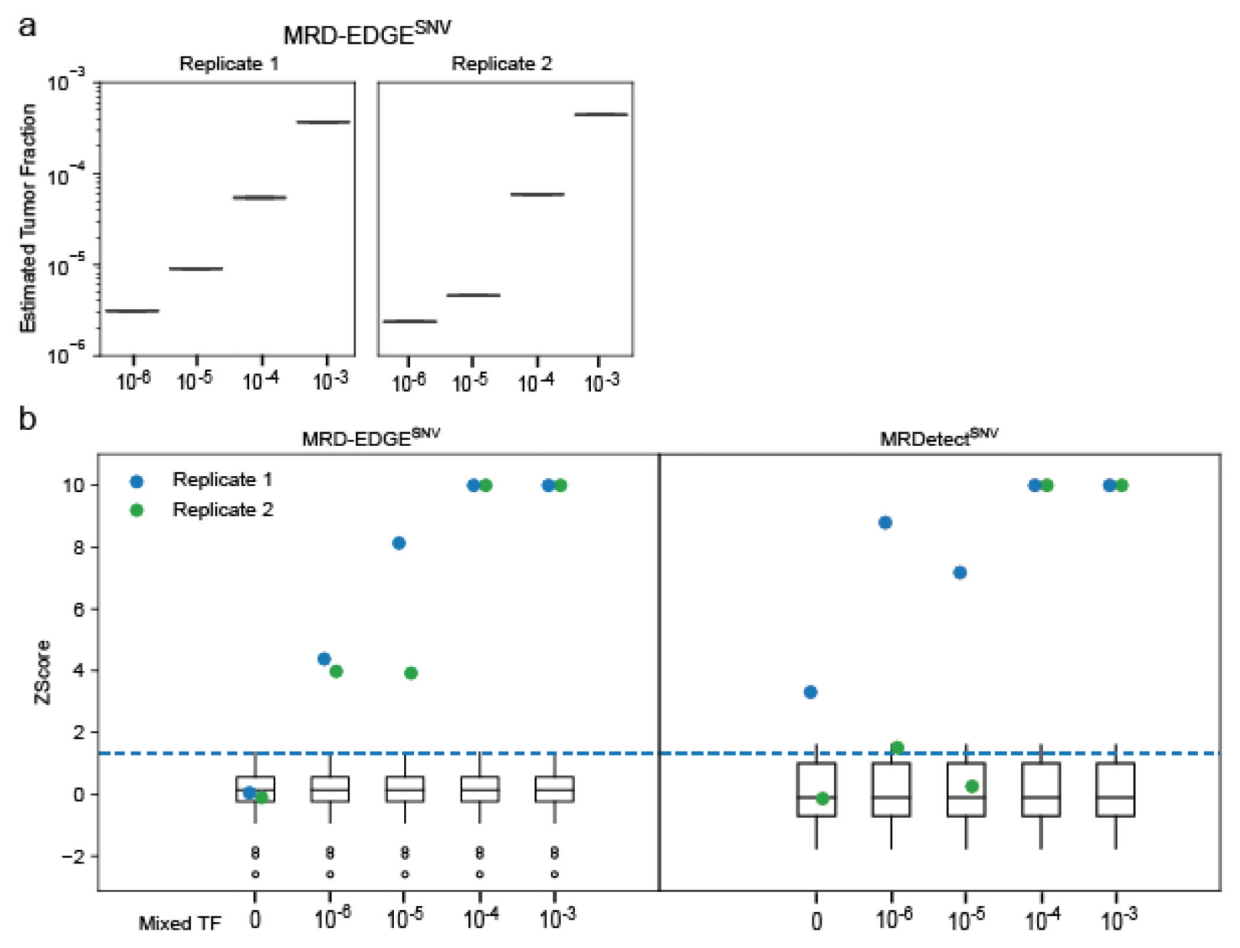
Estimated tumor fractions in experimental mixing studies with MRD-EDGE^SNV^ **a)** Plasma TF inference with MRD-EDGE^SNV^ using genome-wide SNV integration for *in vitro* dilutions of the pretreatment melanoma plasma MEL-137_A in expired plasma harvested through plasmapheresis from a donor without known cancer. Dilutions were performed in 2 replicates, and a mean noise rate for the patient-specific mutation profile was drawn from *n*=17 concurrently sequenced SCLC plasma samples (Supplementary Table 5). **b)** MRD-EDGE^SNV^ (left) and MRDetect^SNV^ (right) Z score discrimination between ctDNA detected in experimental plasma replicates (blue dots, replicate 1, and green dots, replicate 2) from the patient MEL-137 and downsampled TF=0 replicates (white boxes, n=30, 15 downsampled alignment files from 2 TF=0 replicates). Signal is measured from SNV detection rates on patient plasma and the downsampled TF=0 plasma samples using the patient-specific SNV profile for MEL-137. Positive ctDNA detection (dotted blue line) was defined as patient plasma MRD-EDGE^SNV^ or MRDetect^SNV^ Z score above a detection threshold of 95% specificity against downsampled TF=0 plasma in the ROC for each platform (Supplementary Table 4). Sample-level Z scores were capped at 10 to allow greater visibility of Z scores around the detection threshold.

To experimentally validate *in silico* results, we diluted cfDNA from plasma sample from a melanoma patient into healthy donor plasma (Methods), confirming parts per million detection and demonstrating accurate estimated TFs (Methods, Extended Data Fig. 3a-b). Finally, to orthogonally validate TF estimates, we applied MRD-EDGE^SNV^ and digital droplet PCR (ddPCR, Methods) to preoperative plasma samples from 48 patients with stage III CRC. At low TFs (< 4*10^-4^), MRD-EDGE^SNV^ detected 89% (16/18) of samples that were ctDNA-negative by ddPCR. Samples classified as ctDNA-positive by both methods (n = 30) showed high correlation in estimated ctDNA levels (r=0.94 95%-CI: 0.87-0.97, p=1.9*10^-14^, Pearson’s correlation; Fig. 1f-g).

### Multiple features enhance ctDNA detection with MRD-EDGE^CNV^

Aneuploidy is observed in most of solid tumors and is a prominent hallmark of the cancer genome^29^. We have shown that MRDetect-based CNV detection (“MRDetect^CNV^”) can monitor disease burden in cancers with high aneuploidy but low SNV mutation burden using read depth skews^14^. This approach, however, required substantial aneuploidy (>1 Gb altered genome) to detect TFs of 5*10^-5^.

**Extended Data Fig. 4:**
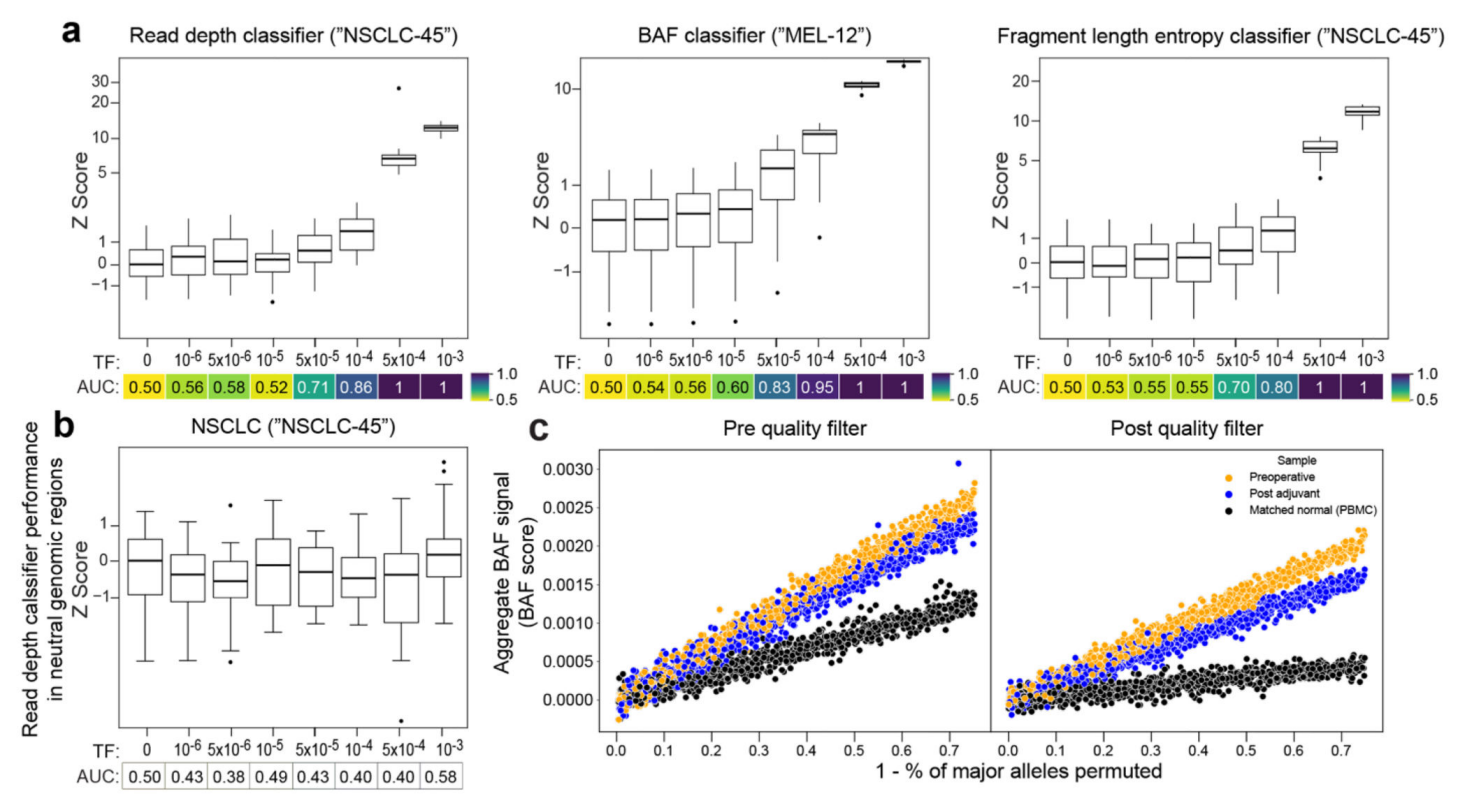
*In silico* mixing studies of MRD-EDGE^CNV^ in CRC, NSCLC, and melanoma **a**,**b**) *In silico* mixing studies in which high TF plasma samples were admixed into non-cancer plasma (**a**) or low TF plasma samples (**b**). Admixtures model tumor fractions of 10^-6^–10^-3^ (see Methods for detailed description of *in silico* admixture process). Box plots represent median, lower and upper quartiles; whiskers correspond to 1.5 x interquartile range. An AUC heatmap demonstrates detection performance vs. TF=0 at different mixed TFs as measured by a sample Z score (derived from summed read-depth skews for read depth classifier, BAF score for BAF classifier, summed fragment length entropy for fragment length entropy classifier, Methods) compared to TF=0 distribution for each replicate. **a**) Pretreatment NSCLC plasma from the patient NSCLC-45 was mixed into non-cancer control plasma from the patient CTRL-206 in 25 technical replicates (each subsampling seed represents a technical replicate). The read depth (left) and fragment length entropy (right) classifiers demonstrate similar performance in pretreatment NSCLC admixtures compared to CRC admixtures (Fig. 2b-d). (middle) Pretreatment melanoma plasma from the patient MEL-12 was mixed into posttreatment plasma following a major response to immunotherapy in 25 technical replicates. The BAF classifier demonstrates similar performance compared to CRC admixtures (Fig. 2c) and accounts for bias that may be encountered when mixing plasma into matched peripheral blood mononuclear cell (PBMC) normal, as performed in CRC. **b**) Z scores for the read depth classifier in neutral regions (no copy number gain or loss in the matched tumor WGS data) for NSCLC demonstrates the expected absence of directional read depth skew in copy neutral regions. **c**) Assessment of preoperative plasma, post adjuvant plasma, and matched normal (from PBMCs) BAF in SNPs before (left) and after (right) SNP quality filters in CRC (patient CRC-465). Filters include mapping bias correction and outlier exclusion criteria (Methods). BAF signal is calculated through least squares linear regression on SNPs from LOH regions identified in matched tumor WGS, accounting for underlying copy number state in tumors (Methods). To demonstrate the relationship between signal and phased SNPs, the major allele in plasma is randomly permuted to be in phase or out of phase at the percentage specified along the x axis. Following quality filtering, signal can be appropriately inferred and demonstrates the expected relationship between preoperative plasma (highest signal), postoperative MRD (intermediate signal), and PBMC BAF (minimal signal).

**Figure 2:**
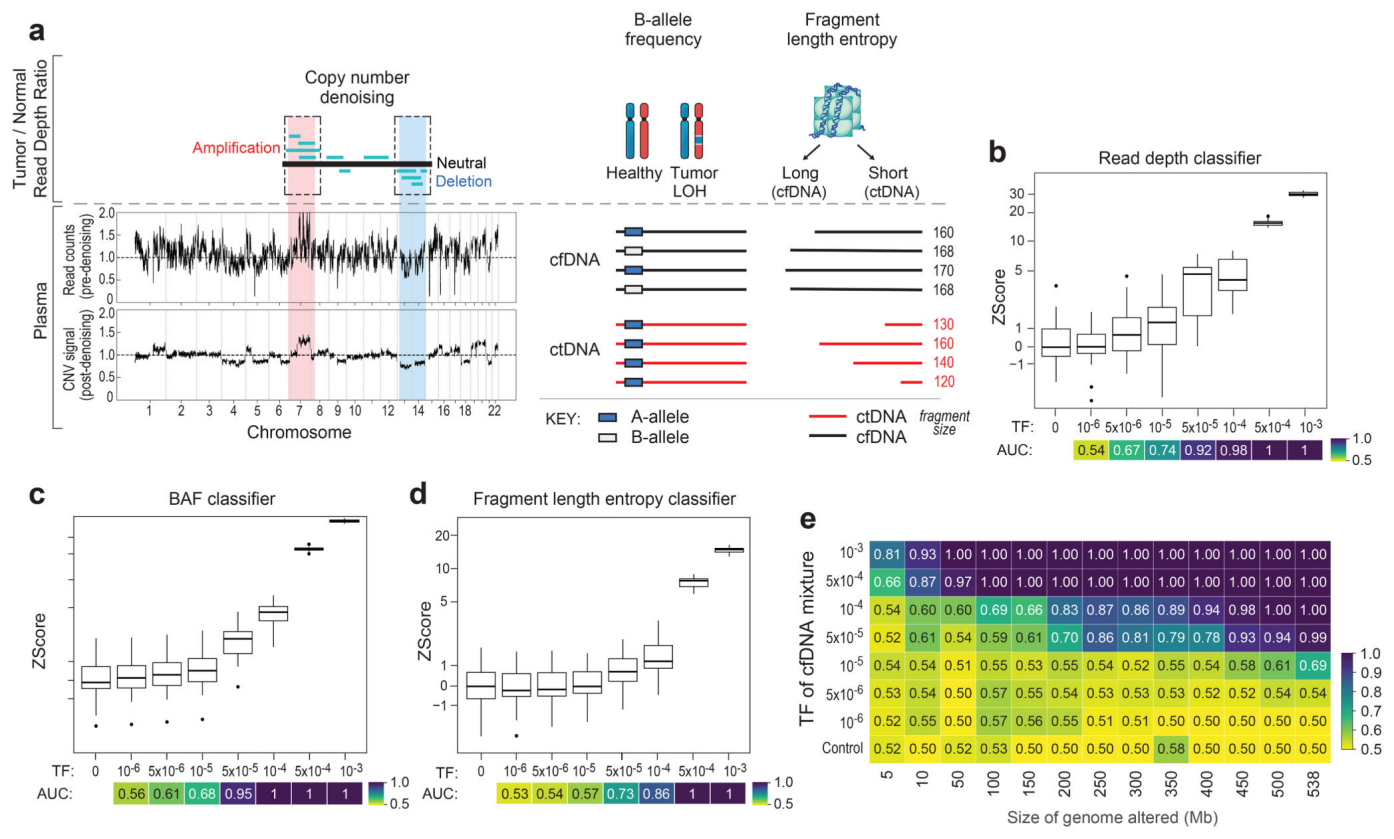
Machine learning-based error suppression and additional features enhance plasma WGS-based CNV detection sensitivity **a)** (left) Copy number denoising for inference of plasma read depth. Patient-specific CNV segments are selected by comparing tumor and germline WGS. In plasma, CNV segments may be obscured within noisy raw read depth profiles. Machine-learning guided denoising using a panel of normal (PON) healthy control plasma samples removes recurrent background noise to produce denoised plasma read depth profiles. PON plasma samples are excluded from downstream CNV analysis. (middle) Loss of heterozygosity (LOH) can be measured via changes in the B-allele frequency of SNPs in cfDNA. (right) Increased or decreased fragment length heterogeneity is expected in regions of tumor amplifications or deletions, respectively, due to varying contribution of ctDNA (shorter fragment size) to the plasma cfDNA pool. Fragment length heterogeneity is measured through Shannon’s entropy of fragment insert sizes. **b-e)** *In silico* mixing studies of admixed high and low TF samples from the CRC patient CRC-930. Pretreatment plasma (TF = 12%) was mixed into non-cancer plasma (CTRL-443, **b** and **d**) or matched PBMC (**c**) in 25 replicates. Admixtures model tumor fractions of 10^-6^–10^-3^. Box plots represent median, lower and upper quartiles; whiskers correspond to 1.5 x interquartile range. An AUC heatmap demonstrates detection performance at the different admixed TFs vs. negative controls (TF=0), measured by Z score (derived from summed read-depth skews for read depth classifier, BAF score for BAF classifier, summed fragment length entropy for fragment length entropy classifier, Methods). **b)** Read depth classifier demonstrates detection sensitivity above TF=0 as low as 5*10^-5^ (AUC 0.92). **c-d)** SNP B-allele frequency (BAF) **(c)** and fragment length entropy **(d)** classifiers demonstrate detection sensitivity at 5*10^-5^ (AUC 0.95 and 0.73, respectively). **e)** Measurement of the MRD-EDGE^CNV^ lower limit of detection for the combined feature set as a function of the CNV load and admixture modeled TF. Sensitive detection (AUC 0.70) is observed at TF = 5*10^−5^ at 200 Mb. Control row is shown for an additional 25 TF=0 seeds held out from downsampling analysis. AUCs were confined to a range of 0.50-1.00.

Detection of subtle read-depth skews in low TF ctDNA may be hindered by biases that arise from sample preparation (e.g., GC bias), alignment (e.g., variable mapping), and biological factors (e.g., replication timing). To correct for such biases, we developed a machine-learning guided CNV denoising platform for plasma WGS^30^. Our plasma read depth classifier uses robust principal component analysis (rPCA) trained on a panel of normal samples (PON) to correct read depth distortions due to background artifacts (Methods, Fig. 2a). To evaluate performance, we admixed *in silico* reads from a high-burden CRC or NSCLC plasma sample into a non-cancer control sample (Supplementary Table 4), identifying signal from read depth skews at TF admixtures as low as 1*10^-5^ (Fig. 2b, Extended Data Fig. 4a, **left**. Lack of directionally skewed signal in copy neutral regions in the matched tumor served as a negative control (Extended Data Fig. 4b).

Loss of heterozygosity (LOH) can also be an important additional source of CNV signal. Copy neutral LOH (cnLOH) cannot be captured by read depth skews but can be measured through allelic imbalances in germline single nucleotide polymorphisms (SNPs) in plasma. Here, inference of the major alleles in LOH and amplification regions is derived from tumor WGS^31,32^ and the B-allele frequency (BAF) in plasma is indicative of ctDNA signal (Fig. 2a). Our BAF classifier aggregates SNPs across these regions (see Methods for quality filters, Extended Data Fig. 4c, and estimates sample-wide plasma allelic imbalance from a least squares linear regression accounting for regional copy number state (Methods). To validate this approach, we created *in silico* admixtures by diluting reads from plasma WGS of a CRC or melanoma patient into their matched peripheral blood mononuclear cells WGS to maintain SNP phasing consistency (Supplementary Table 4), identifying allelic imbalance signal in TF admixtures as low as 5*10^-5^ (Fig. 2c, Extended Data Fig 4a, **middle**).

Finally, we leveraged well-characterized abnormal ctDNA fragmentation patterns^20,22,33–35^ as an additional source of aneuploidy signal. ctDNA is associated with more heterogenous fragment lengths than normal cfDNA^33,34^. We therefore measured fragment length entropy (Methods) in plasma WGS segments. While existing approaches identify altered fragmentation profiles inherently or compared to non-cancer plasma^33,36^, our fragment length entropy classifier compares cfDNA fragment lengths in tumor-informed CNV segments versus copy-neutral segments, overcoming sample-level technical variation in fragment length. Thus, in regions with copy number amplifications, we anticipate greater fragment length entropy due to a larger contribution of ctDNA fragments, whereas in deletions less plasma fragment length entropy is expected due to lower ctDNA contribution. In our *in silico* admixtures, our fragment length entropy classifier identified signal in TFs as low as 5*10^-5^ (Fig. 2d, Extended Data Fig 4a, **right**, discrimination between mixed TFs for each CNV classifier Supplementary Fig. 3).

Read depth, BAF, and fragment length entropy serve as independent and complementary sources of CNV signal. MRD-EDGE^CNV^ combines signals from these classifiers as independent inputs at the sample level to comprehensively assess for plasma TF (Methods). Because the aneuploidy signal in plasma WGS is a function of both the proportion of the cancer genome affected by aneuploidy and the TF, we evaluated classifier performance by downsampling both the TF (as above in Fig. 2b-d) and the cumulative size of CNV segments to characterize a LLOD matrix (Fig. 2e). As expected, classifier performance improved with increased aneuploidy. While MRDetect required 1 Gb of aneuploidy^14^ for a LLOD of 5*10^-5^, MRD-EDGE^CNV^ achieved the same LLOD with only 200 Mb of aneuploidy, extending applicability to many solid tumors^37^.

### MRD-EDGE detects early-stage cancer and postoperative MRD

In our WGS tumor-informed ctDNA detection paradigm, tumor WGS provides patient-specific SNVs and CNVs. Sample ctDNA enrichment is measured as a Z score for the matched patients’ plasma signal against a noise distribution generated by applying the patient-specific SNVs and CNVs to unmatched non-cancer (control) plasma samples (Methods). Throughout the study, a Z score exceeding the 95% specificity threshold in the control noise distribution was used as the ctDNA detection threshold.

**Extended Data Fig. 5:**
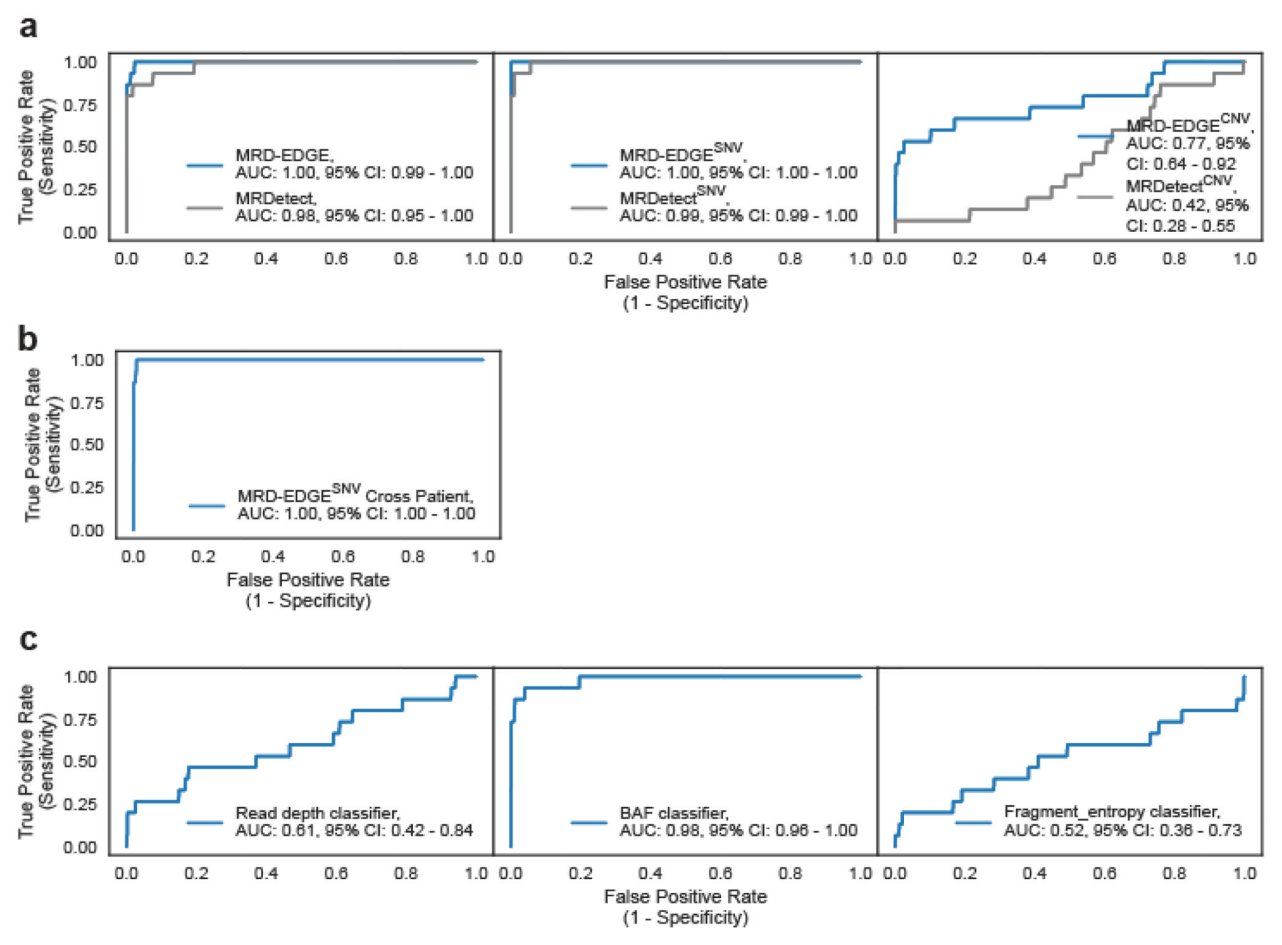
Clinical performance of tumor-informed MRD-EDGE in stage III perioperative colorectal cancer **a)** (left) ROC analysis on MRD-EDGE (blue), a combined detection model of SNV and CNV mutation profiles, and MRDetect (gray) in preoperative stage III CRC. Preoperative plasma samples with matched tumor mutation profiles (*n*=15, Supplementary Table 5) are compared with control plasma samples assessed against all unmatched stage III CRC tumor mutation profiles (*n*=15 tumor profiles assessed across 25 control samples from Aarhus controls cohort, *n*=375 control-comparisons). Twenty control samples included in SNV model training and / or used in the MRD-EDGE^CNV^ read depth panel of normals were withheld from this analysis. (middle) ROC analysis with MRD-EDGE^SNV^ (blue), and MRDetect^SNV^ (gray). Preoperative plasma samples with matched tumor mutation profiles (*n*=15) are compared with unmatched control plasma samples assessed against all unmatched stage III CRC tumor mutation profiles (*n*=15 tumor profiles assessed across 40 control samples from Aarhus controls cohort, *n*=600 control-comparisons). Five control samples included in SNV model training were withheld from this analysis. (right) ROC analysis with MRD-EDGE^CNV^ (blue), and MRDetect^CNV^ (gray). Preoperative plasma sample CNV-based Z scores (*n*=15) are compared against control plasma samples assessed against all unmatched stage III CRC tumor mutation profiles (*n*=15 tumor profiles assessed across 25 control samples from Aarhus controls cohort, *n*=375 control-comparisons). Twenty control samples included in the read depth panel of normals were withheld from this analysis. **b)** Cross-patient ROC analysis on preoperative stage III CRC plasma samples for MRD-EDGE^SNV^ demonstrates similar performance to control (non-cancer) plasma. Preoperative plasma samples with matched tumor profiles (*n*=15) are compared with stage III CRC plasma samples assessed against all unmatched stage III CRC tumor profiles (*n*=15 tumor profiles assessed across 14 cross-patient samples, *n*=210 cross-comparisons). **c)** ROC analysis performed on CNV-based Z-score values for read depth (left), BAF (middle), and fragment length entropy (right) CNV classifiers in preoperative stage III CRC. Preoperative plasma samples with matched tumor profiles (*n*=15) are compared with control plasma samples assessed against all unmatched tumor profiles (*n*=375 comparisons for read depth, 15 tumor profiles assessed across 25 control samples; *n*=675 comparisons for BAF and fragment length entropy, 15 tumor profiles assessed across 45 control samples). Twenty control samples included in the read depth panel of normal samples were withheld from read-depth analysis.

**Figure 3:**
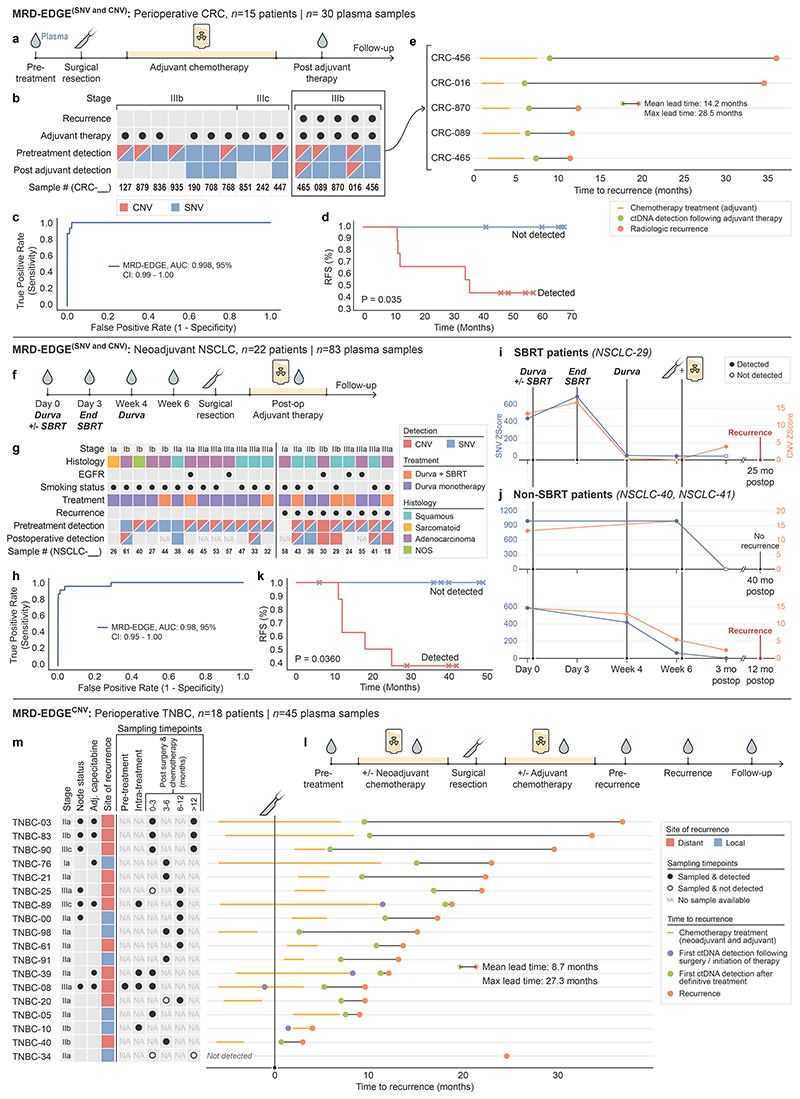
Tumor-informed monitoring of minimal residual disease in perioperative, neoadjuvant, and recurrent disease settings **a**) Perioperative colorectal cancer ctDNA assessment. Plasma TF is tracked prior to surgery, and after surgery and adjuvant chemotherapy. **b**) Clinical characteristics and detection status of the stage III CRC cohort. **c**) ROC analysis on MRD-EDGE in preoperative stage III CRC with matched tumor mutation profiles (*n*=15) compared to control plasma samples assessed against all unmatched stage III CRC tumor mutation profiles (*n*=15 tumor profiles assessed across 25 control samples from Aarhus controls cohort, *n*=375 control-comparisons). **d**) Kaplan–Meier disease-free survival analysis of all patients with detected (*n*=9) and non-detected (*n*=6) postoperative ctDNA. Postoperative ctDNA detection was associated with shorter recurrence-free survival (two-sided log-rank test). **e**) Time to recurrence in stage III CRC patients with disease recurrence (*n*=5) after ctDNA detection post-therapy (green dot). Red dot indicates confirmed recurrence on CT imaging. **f**) Neoadjuvant NSCLC clinical treatment protocol^41^. Plasma TF is tracked in the preoperative period to evaluate for response to SBRT and ICI (durvalumab) therapy and after surgery to evaluate for MRD. **g**) Clinical characteristics and detection status of the neoadjuvant NSCLC cohort (*n*=22 patients). **h)** ROC analysis on MRD-EDGE in pretreatment early-stage NSCLC. Preoperative plasma samples with matched tumor mutation profiles (*n*=22) are compared with control samples assessed against all unmatched NSCLC tumor mutation profiles (*n*=22 mutation profiles assessed across 20 control samples from NYGC control cohort, *n*=440 control-comparisons). **i**) Tumor burden monitoring on neoadjuvant immunotherapy and SBRT with MRD-EDGE^SNV^ (blue) and MRD-EDGE^CNV^ (orange) Tumor burden estimates are measured as the Z score of the patient tumor mutation profile against healthy control plasma. **j**) Tumor burden monitoring with MRD-EDGE^SNV^ and MRD-EDGE^CNV^ in 2 NSCLC patients on neoadjuvant ICI monotherapy (top, NSCLC-40; bottom, NSCLC-41). Red dot indicates recurrence; black dot indicates absence of recurrence at last known follow-up. **k**) Kaplan–Meier disease-free survival analysis of all patients with detected (*n*=8) and non-detected (*n*=6) postoperative ctDNA. Postoperative ctDNA detection was associated with shorter recurrence-free survival (two-sided log-rank test). **i)** Observational TNBC recurrence cohort. Early-stage TNBC patients underwent surgical resection plus neoadjuvant and/or adjuvant chemotherapy. Plasma was sampled intermittently throughout clinical course. **m)** (left) Clinical characteristics and sampling timepoints for the observational TNBC recurrence cohort (*n*=18 patients). (right) Lead-time calculations for ctDNA detection post-therapy (green dot) versus clinical recurrence (red dot). Where available, purple dot shows ctDNA detection after surgery or initiation of chemotherapy.

We first applied tumor-informed MRD-EDGE to perioperative plasma samples from patients with stage III CRC (*n*=15) compared to controls without known cancer (*n*=40, Supplementary Table 5). Samples were drawn preoperatively and postoperatively following completion of adjuvant chemotherapy (ACT, mean 8.1 months after surgery, Fig. 3a-b). Any samples used in machine learning model training (Supplementary Table 1) were excluded from clinical analyses. AUC for tumor-informed preoperative ctDNA detection with MRD-EDGE was 0.998 (95% CI: 0.99-1.00, Fig. 3c, Extended Data Fig. 5). MRD-EDGE^SNV^ maintained performance in a cross-patient analysis (Methods) that compared the detection in plasma WGS of patient-specific SNVs in matched versus unmatched preoperative plasma from CRC patients (Extended Data Fig. 5b).

In samples drawn after ACT completion, MRD-EDGE detected MRD in 9 / 15 patients, 5 of whom later had disease recurrence. Post-adjuvant MRD was associated with shorter disease-free survival (Fig. 3d) over a median 54 months (range 37.4 – 67.2) of follow-up. Recurrence was not observed in any of the 6 patients without ctDNA detection. A lead time analysis at this first available timepoint after definitive treatment found lead times of 14.2 months (range 4.1 - 28.5 months, Fig. 3e), which compares favorably to lead times in leading bespoke panels (8.7 months)^38^. The 4 patients with positive MRD-EDGE detection with no evidence of recurrence may be due to late recurrence not captured in the available follow-up or to false positive detection, as has been observed for WGS ^14,39^ and leading bespoke panels^8–10^ including after adjuvant therapy in CRC^40^.

MRD-EDGE maintained robust performance in CRC and NSCLC samples from our previous manuscript^29^ (Supplementary Fig. 4-8).

### Tracking plasma TF throughout neoadjuvant therapy in NSCLC

We next applied MRD-EDGE to the challenging setting of tracking plasma tumor burden in response to neoadjuvant therapy. Disease burden monitoring during neoadjuvant therapy could help optimize care during the crucial period between early-stage cancer detection and definitive surgery. We evaluated plasma from 22 early-stage NSCLC patients on a neoadjuvant immunotherapy protocol [NCT02904954^41^] that randomized early-stage bulky NSCLC patients to treatment with the immune checkpoint inhibition (ICI) agent durvalumab, with or without stereotactic body radiation therapy (SBRT), followed by surgical resection (Fig. 3f-g).

**Extended Data Fig. 6:**
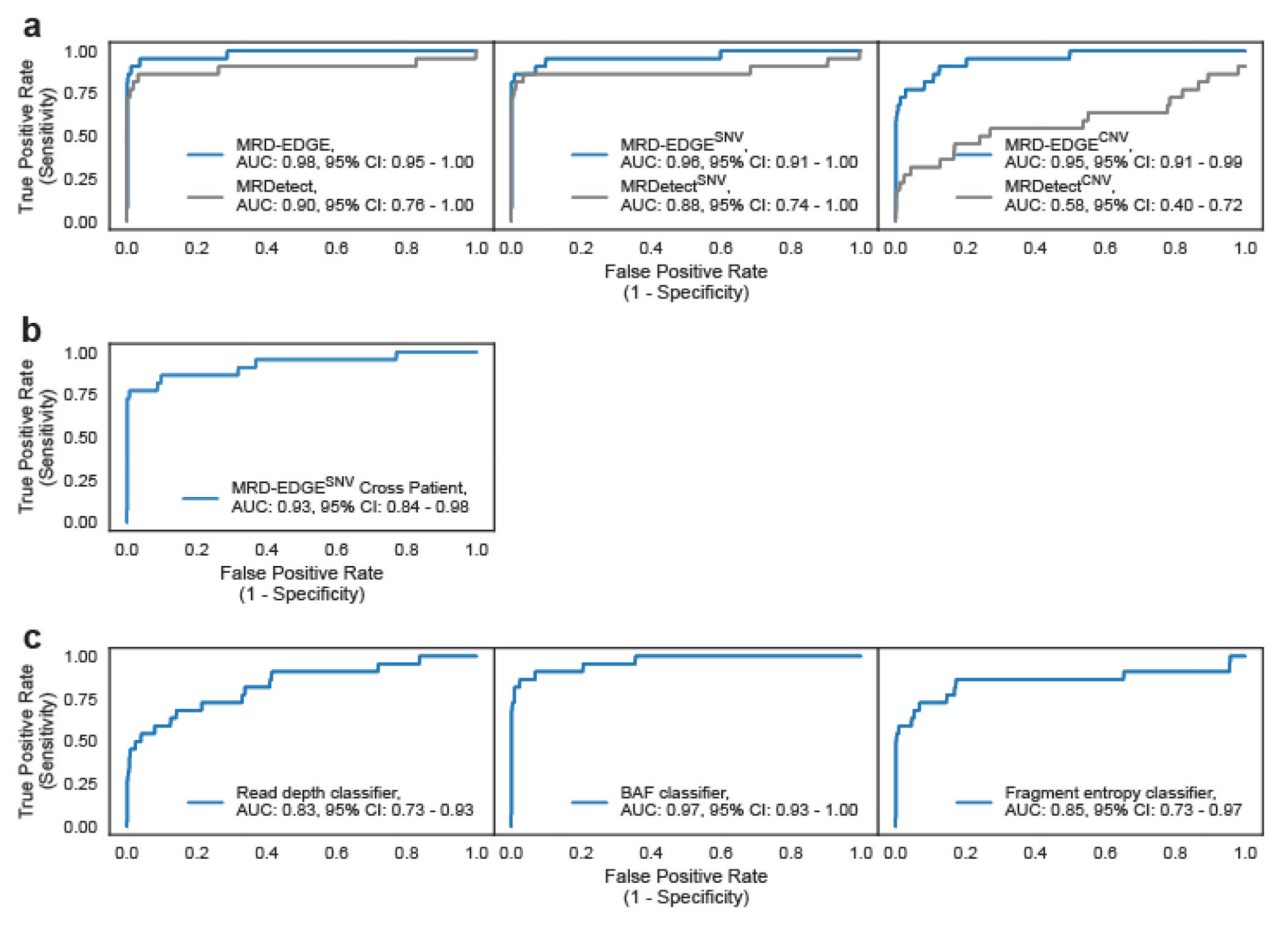
Comparison of MRD-EDGE and MRDetect in preoperative, pretreatment NSCLC **a)** (left) ROC analysis of NSCLC plasma samples for MRD-EDGE (blue) and MRDetect (gray). NSCLC plasma samples with matched tumor profiles (*n*=22 samples, Supplementary Table 5) are compared with control plasma samples assessed against all unmatched NSCLC tumor mutation profiles (*n*=22 tumor profiles assessed across 20 control samples from NYGC controls cohort, *n*=440 control-comparisons). (middle) ROC analysis of NSCLC plasma samples for MRD-EDGE^SNV^ (blue) and MRDetect^SNV^ (gray). NSCLC plasma samples with matched tumor profiles (*n*=22, Supplementary Table 5) are compared with control plasma samples assessed against all unmatched NSCLC tumor mutation profiles (*n*=22 tumor profiles assessed across 40 control samples from NYGC controls cohort, *n*=660 control-comparisons). Five patients used in MRD-EDGE^SNV^ NSCLC model training were excluded from downstream analysis. (right) ROC analysis of NSCLC plasma samples for MRD-EDGE^CNV^ (blue) and MRDetect^CNV^ (gray). NSCLC plasma samples with matched tumor profiles (*n*=22, Supplementary Table 5) are compared against control plasma samples assessed against all unmatched NSCLC tumor mutation profiles (*n*=22 tumor profiles assessed across 20 control samples from NYGC controls cohort, *n*=440 control-comparisons). Fifteen patients used in the read depth panel of normal samples were excluded from downstream analysis. **b)** Cross-patient ROC analysis on pretreatment NSCLC tumor profiles for MRD-EDGE^SNV^ demonstrates similar performance to control (non-cancer) plasma. Preoperative plasma samples with matched tumor profiles (*n*=22) are compared with NSCLC plasma samples assessed against all unmatched NSCLC tumor profiles (*n*=22 tumor profiles assessed across 21 cross-patient samples, *n*=462 cross-comparisons). **c)** ROC analysis performed on CNV-based Z-score values for read depth (left), BAF (middle), and fragment length entropy (right) CNV classifiers in preoperative stage III CRC. Preoperative plasma samples with matched tumor profiles (*n*=22) are compared with control plasma samples assessed against all unmatched tumor profiles (*n*=440 comparisons for read depth, 22 tumor profiles assessed across 20 control samples; *n*=770 comparisons for BAF and fragment length entropy, 22 tumor profiles assessed across 35 control samples). Twenty control samples included in the read depth panel of normal samples were withheld from read-depth analysis.

MRD-EDGE was highly sensitive for pretreatment cancer (AUC 0.98: 95% CI 0.95-1.00, Fig. 3h and Extended Data Fig. 6), as ctDNA was undetectable in only two patients with clinical stage IA disease. Serial sampling allowed us to observe ctDNA kinetics in the neoadjuvant period. In patients who received durvalumab and SBRT, ctDNA shedding increased during SBRT, as is seen with ddPCR^42^, and subsequently decreased at Week 4 following SBRT treatment (Fig. 3i), demonstrating dynamic TF trends during and after radiation. For patients on durvalumab monotherapy, MRD-EDGE monitored TF trends throughout the neoadjuvant treatment period, reflecting a response (Fig. 3j, **bottom**) or lack of response to ICI (Fig. 3j, **top**).

Sixteen patients had postoperative plasma available for WGS analysis; at the time of surgery, 2 patients had unresectable tumors and were excluded from our survival analyses. Among the 14 patients that underwent surgical resection, MRD detection was associated with shorter disease-free survival (*P*=3.6*10^-2^, logrank test, Fig. 3k). None of the MRD-negative patients (*n*=6) had recurrence, while recurrence was observed in 5/8 of MRD positive patients. As postoperative samples analyzed for MRD were drawn amid adjuvant therapy, including immunotherapy, chemotherapy, or chemoradiation therapy (Supplementary Table 6), the detection of residual disease in patients without recurrence may be due to short follow-up (cohort median 30 months), clearance of residual disease following adjuvant therapy, or may indicate false positive results.

### MRD-EDGE^CNV^ in monitoring of high aneuploidy tumors

To demonstrate the value of standalone MRD-EDGE^CNV^ ctDNA monitoring in a high aneuploidy but low mutational burden solid tumor, we evaluated serial plasma samples from an observational cohort of triple negative breast cancer (TNBC) patients (*n*=18) with disease recurrence after definitive therapy (surgery combined with neoadjuvant (*n*=9) or adjuvant (*n*=9) chemotherapy, Fig. 3l-m, Supplementary Table 6).

MRD-EDGE^CNV^ demonstrated strong sensitivity for MRD in this cohort, as ctDNA was detected following treatment initiation (neoadjuvant chemotherapy or surgery) and prior to recurrence in 17 of 18 patients (94.4%, Fig. 3m). As in other tumor-informed analyses, positive detection required an MRD-EDGE^CNV^ Z score in excess of 95% specificity in the control noise distribution (Supplementary Table 7). Following completion of definitive treatment (surgery and ACT), average lead time of ctDNA detection was 9 months and maximum lead-time was 27 months (Fig. 3m), competitive with leading bespoke panels^43^ in TNBC despite sparse sampling at varying time points.

### Detection of ctDNA shedding in adenomas and pT1 carcinomas

Whether noninvasive (precancerous) lesions shed ctDNA is unknown. While tumor-informed MRD-EDGE cannot be used for screening, the exquisite sensitivity of our approach allowed us to address whether ctDNA is shed from adenomas and polyp cancers (pT1pN0), where ctDNA detection through existing methods such as ddPCR has been limited^44–46^.

**Figure 4:**
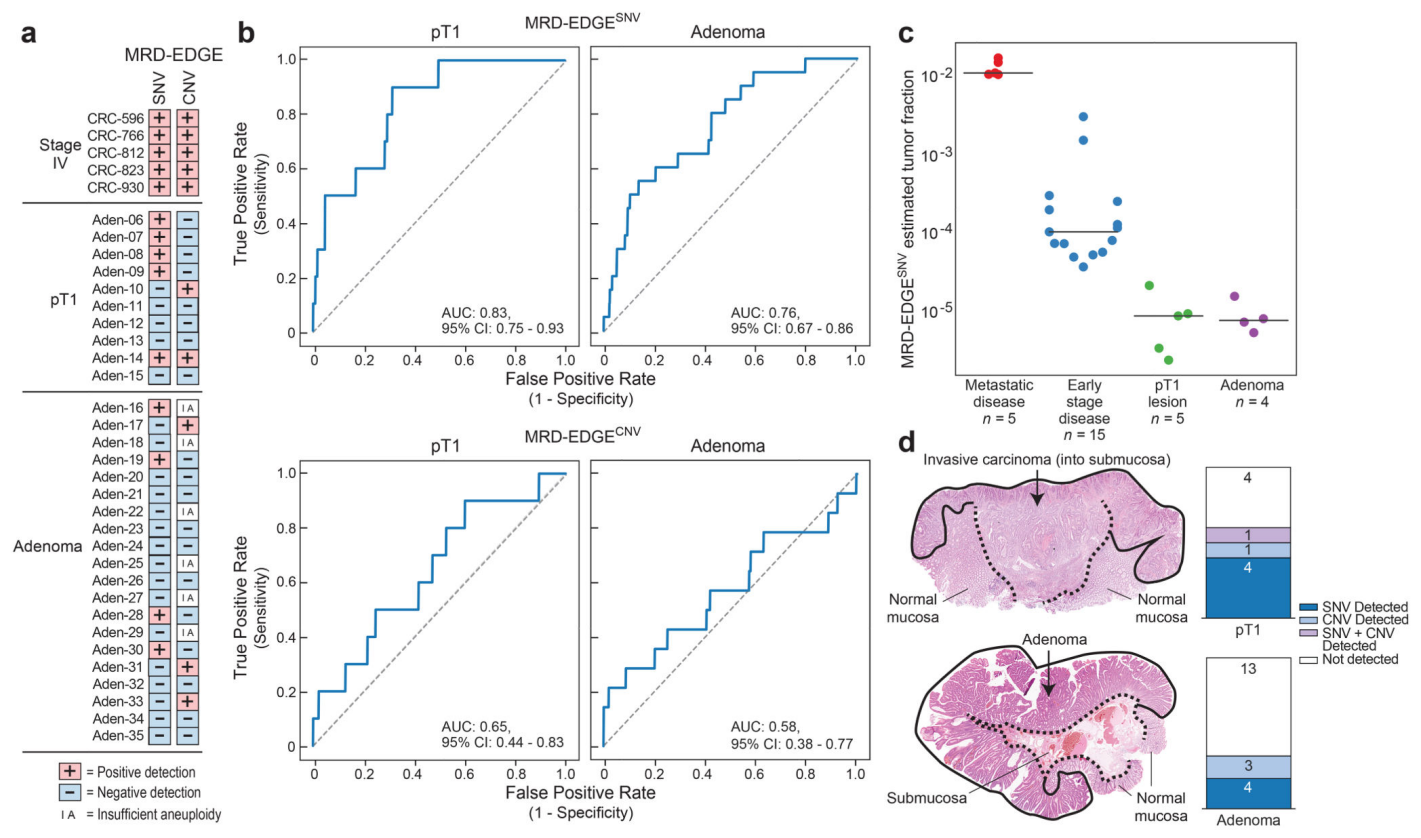
MRD-EDGE tumor-informed detection of ctDNA from screen-detected adenomas and pT1 lesions **a)** Detection status of the cohort of stage IV colorectal (CRC, *n*=5), screen-detected pT1 lesions (*n*=10) and screen-detected adenoma plasma samples (*n*=20) according to MRD-EDGE^SNV^ and MRD-EDGE^CNV^ classifiers. Samples with a Z score above the detection threshold as prespecified in the stage III CRC cohort (Fig. 3a-b) are highlighted (Supplementary Table 7). **b)** ROC analysis for MRD-EDGE^SNV^ (top) and MRD-EDGE^CNV^ (bottom) classifiers in screen-detected pT1 lesions (left) and adenomas (right) compared to cancer-free control plasma samples. The SNV analysis excluded 5 Aarhus control samples (n=45 total Aarhus control plasma samples) used in SNV model training, yielding *n=*40 controls as a comparator. The CNV analysis excluded 20 Aarhus control samples used in the panel of normal samples, yielding *n=*25 control samples as a comparator. **c)** Plasma TF inference using genome-wide SNV integration for stage IV CRC (*n*=5), stage III CRC (*n*=15), SNV detected pT1 lesions (*n*=5), and SNV detected adenomas (*n*=4) shows decreasing estimated TF by CRC stage. Lines indicate median estimated TF. **d)** (left) Histology image of the pT1 lesion Aden-14 (top) demonstrates invasion of the submucosa by dysplastic cancer cells, while an image of the adenoma Aden-17 (bottom) demonstrates the presence of dysplasia and absence of submucosal invasion. (right) Barplots demonstrate number of plasma samples with detected ctDNA in patients with pT1 lesions (top) and adenomas (bottom). Detections are shaded by dark blue (MRD-EDGE^SNV^ detections), light blue (MRD-EDGE^CNV^ detections), light purple (SNV and CNV detections), and white (non-detected).

We evaluated pre-resection plasma from 30 patients with early lesions detected through screening^47^ (Fig. 4). Ten patients had pT1 lesions (defined as invasion of the submucosa but not the muscular layer, Fig. 4d), and 20 patients had screen-detected precancerous adenomas (Fig. 4a). Consistent with prior reports^48–50^, we found decreased aneuploidy in adenomas (median 235 Mb of genome-wide aneuploidy) compared to our stage III CRC samples (median 1.2 GB, *P*=2.8*10^-6^).

**Extended Data Fig. 7:**
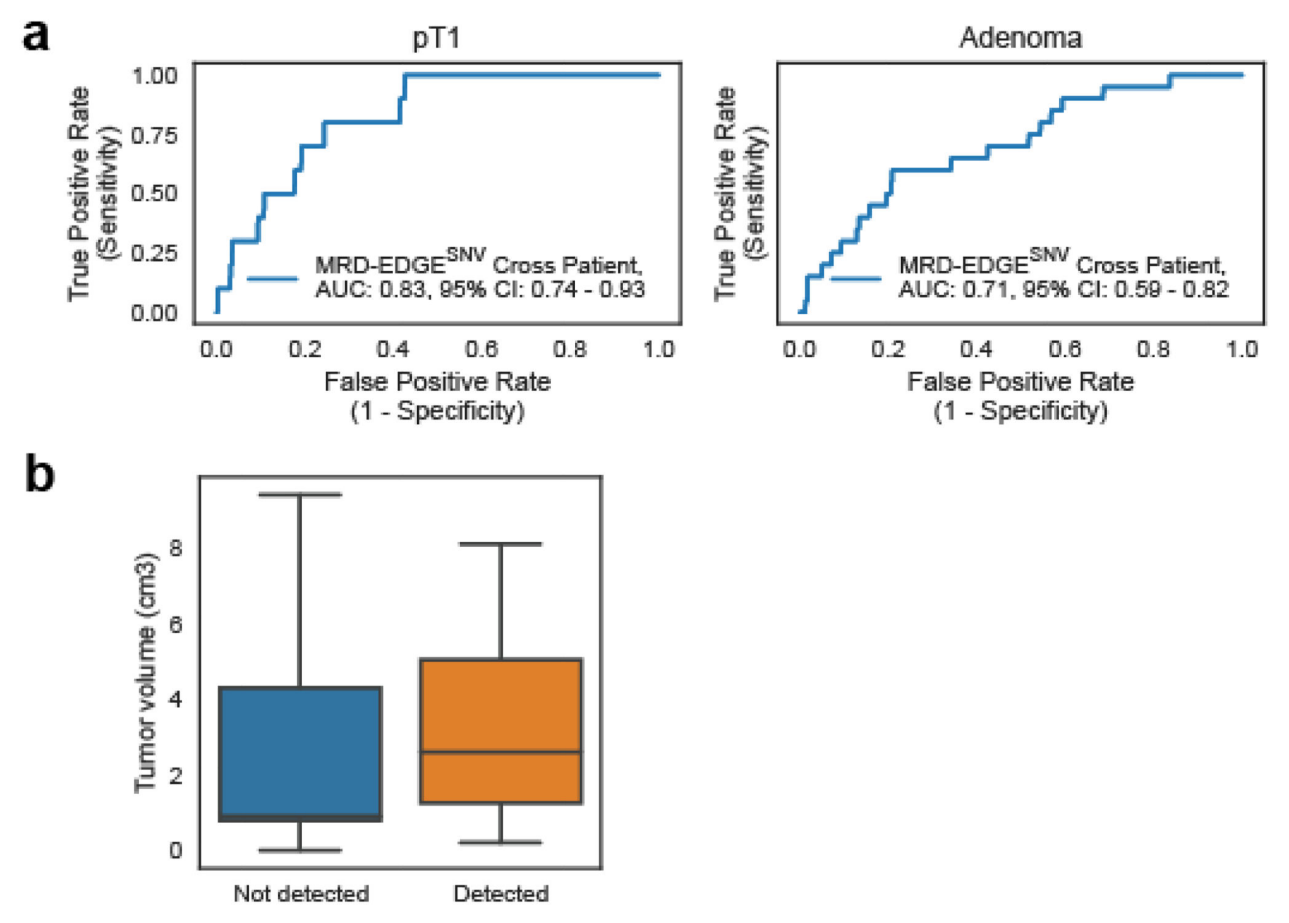
MRD-EDGE detection of ctDNA from colorectal pT1 carcinomas and adenomas **a)** Cross-patient ROC analysis for MRD-EDGE^SNV^ in screen-detected pT1 lesions (left) and adenomas (right). Preoperative plasma samples with matched tumor mutation profiles are compared with a cross-patient panel of plasma samples assessed against all unmatched cross-patient tumor profiles (*n*=44, including 29 pT1 and adenoma cross patients and 15 stage III preoperative patients). **b)** Tumor resection volume for adenoma samples in which ctDNA was detected (orange) and non-detected (blue). Box plots represent median, bottom and upper quartiles; whiskers correspond to 1.5 x interquartile range.

We compared these samples to healthy control plasma samples (*n*=40), using a prespecified ctDNA detection threshold value drawn from our preoperative stage III CRC cohort (Fig. 3a-b). MRD-EDGE detected ctDNA shedding in 6 / 10 (60%) pT1 lesions and 7 / 20 (35%) precancerous adenomas (Fig. 4a-d, cross-patient Extended Data Fig. 7a, clinical features Extended Data Fig. 7b, Supplementary Table 8). Detection AUCs were higher for pT1 lesions than adenomas, as expected (Fig. 4b). We further found lower estimated TFs in detected adenomas (median 8.0*10^-6^) and pT1 lesions (median 9.1*10^-6^) than stage III (median 1.1*10^-4^) and metastatic (median 1.2*10^-2^) CRC samples (Fig. 4c, for description of additional detection metrics see Supplementary Note). These data demonstrate that even without a significant invasive component, dysplastic tissue may shed ctDNA, forming an important consideration as advanced non-tumor informed methods are deployed for early cancer detection efforts.

### Plasma-only ctDNA monitoring in metastatic disease

In prior bespoke panel studies, a substantial proportion of eligible patients were excluded due to low tumor DNA purity or absence of matched tumor tissue^2,43,51^, introducing the need for plasma-only ctDNA detection in clinical application.

Tumor-informed approaches consider only positions in the genome that overlap with tumor SNVs, thereby excluding the vast majority of non-overlapping sequencing artifacts. Without matched tissue, we can instead form a sample-level ctDNA detection rate defined as the number of SNV fragments classified as ctDNA over all cfDNA SNV fragments evaluated (Methods). Because we evaluate all cfDNA fragments that contain a variant (~10^8^ per plasma WGS, Supplementary Table 5), plasma-only (non-tumor informed) fragment classification requires a higher specificity threshold than the fragment classification in the tumor-informed setting (for optimal specificity calculation see Methods, Supplementary Fig. 9).

**Figure 5:**
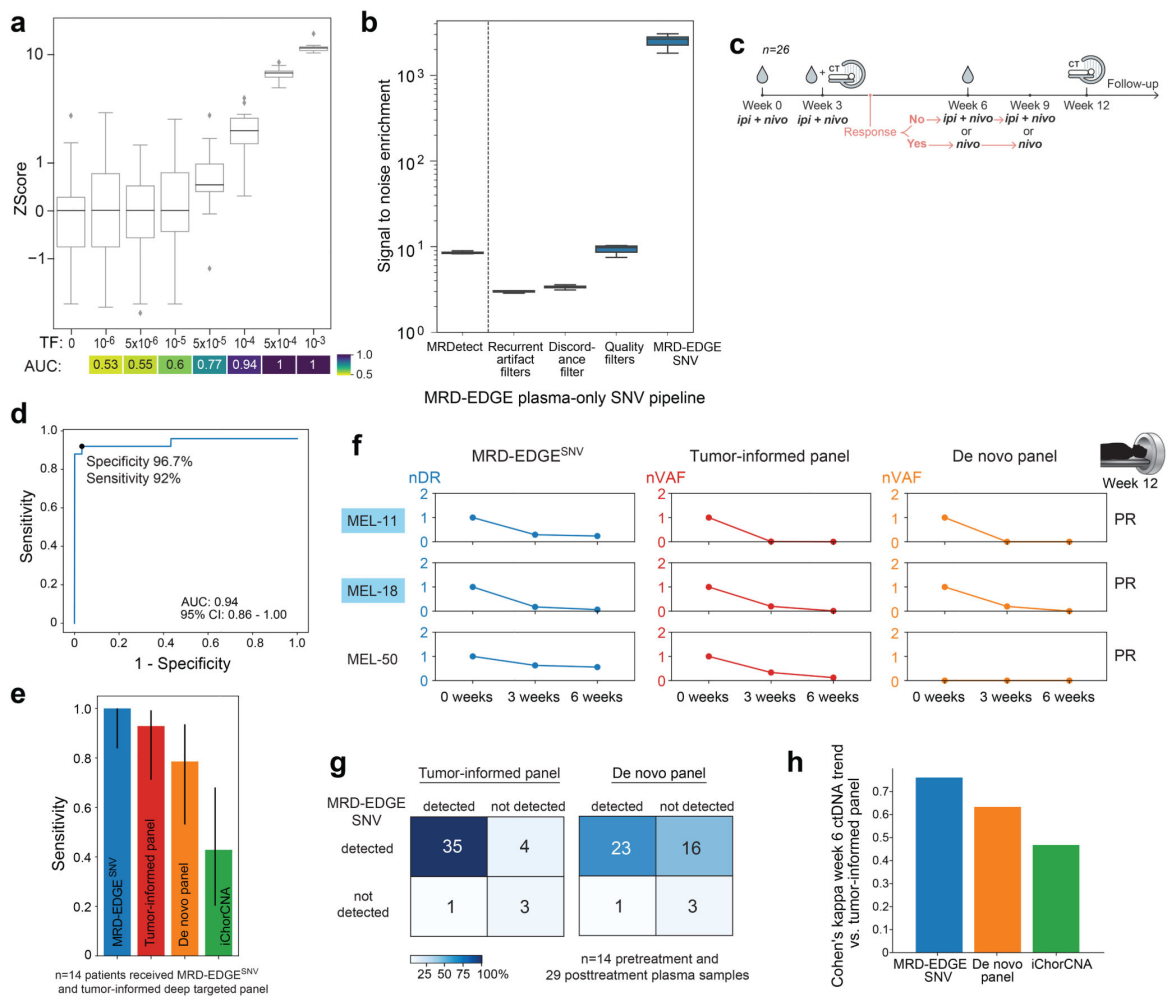
ctDNA detection in melanoma plasma WGS without matched tumor **a)** *In silico* mixing of cfDNA from metastatic melanoma sample MEL-100 (TF = 6.1%) into control cfDNA (TF=0) at mix fractions 10^-6^–10^-3^ at 16X coverage depth (20 technical replicates). MRD-EDGE^SNV^ detects TF as low as TF=5*10^-5^ (AUC 0.77), measured by Z score of SNV fragment detection rates against unmixed control plasma (TF=0), without matched tumor tissue. AUC heatmap measures detection vs. TF=0 at different mixed TFs. **b)** Signal-to-noise enrichment analysis for MRDetect^SNV^ and staged steps of MRD-EDGE^SNV^ using the same *in silico* mixing replicates as in **a)**. MRD-EDGE^SNV^ produces 2,518-fold enrichment vs. 8.3-fold for MRDetect^SNV^. **c)** Adaptive dosing melanoma cohort (*n*=26 patients). All patients began treatment with combination ipilimumab and nivolumab. **d)** ROC analysis for MRD-EDGE^SNV^ detection of pretreatment melanoma for healthy individuals (*n*=30) and melanoma patients (*n*=25). Detection rate cutoff was selected as the first operational point with specificity ≥ 95%. **e)** Fourteen of 26 melanoma patients underwent tumor-informed targeted panel^7^ sequencing. Barplot demonstrates pretreatment detection sensitivity for MRD-EDGE^SNV^, tumor-informed panel, *de novo* panel (Methods) and ichorCNA. Error bars indicate 95% binomial confidence interval for empiric sensitivity within 14 trials. **f)** Tumor burden monitoring on ICI with MRD-EDGE^SNV^, tumor-informed panel, and *de novo* panel for 3 melanoma patients, measured as normalized detection rate (nDR) to the pretreatment sample (MRD-EDGE^SNV^) and as normalized variant allele fraction (nVAF) normalized to the pretreatment VAF (tumor-informed and *de novo* panels). Blue name indicates samples with ≥14 SNVs covered in the tumor-informed panel. **g)** Forty-three pre- and posttreatment samples from the melanoma cohort underwent sequencing with MRD-EDGE^SNV^ and the tumor-informed panel. (left) Heatmap demonstrating high concordance (88%) between MRD-EDGE^SNV^ and the tumor-informed panel for detected ctDNA and undetectable ctDNA. (right) Lower detection overlap (60%) is seen between MRD-EDGE^SNV^ and the *de novo* targeted panel. **h)** Barplot of Cohen’s kappa agreement metric for Week 6 ctDNA increase or decrease compared to pretreatment baseline between 3 mutation callers (MRD-EDGE^SNV^, *de novo* panel, ichorCNA) and the tumor-informed panel. Box plots-median, bottom and upper quartiles; whiskers- 1.5 x interquartile range.

To evaluate our plasma-only MRD-EDGE^SNV^ approach, we first evaluated LLOD by using the same *in silico* admixtures used in the tumor-informed setting (Fig. 1e). Here, we found plasma-only detection of TF admixtures at 5*10^-5^ (AUC 0.77 for discrimination against TF=0 as controls, Fig. 5a). To benchmark performance improvement relative to our prior work^14^, we compared signal-to-noise enrichment for MRD-EDGE^SNV^ with MRDetect^SNV^ and found 301-fold (Fig. 5b) higher enrichment for MRD-EDGE^SNV^.

**Extended Data Fig. 8:**
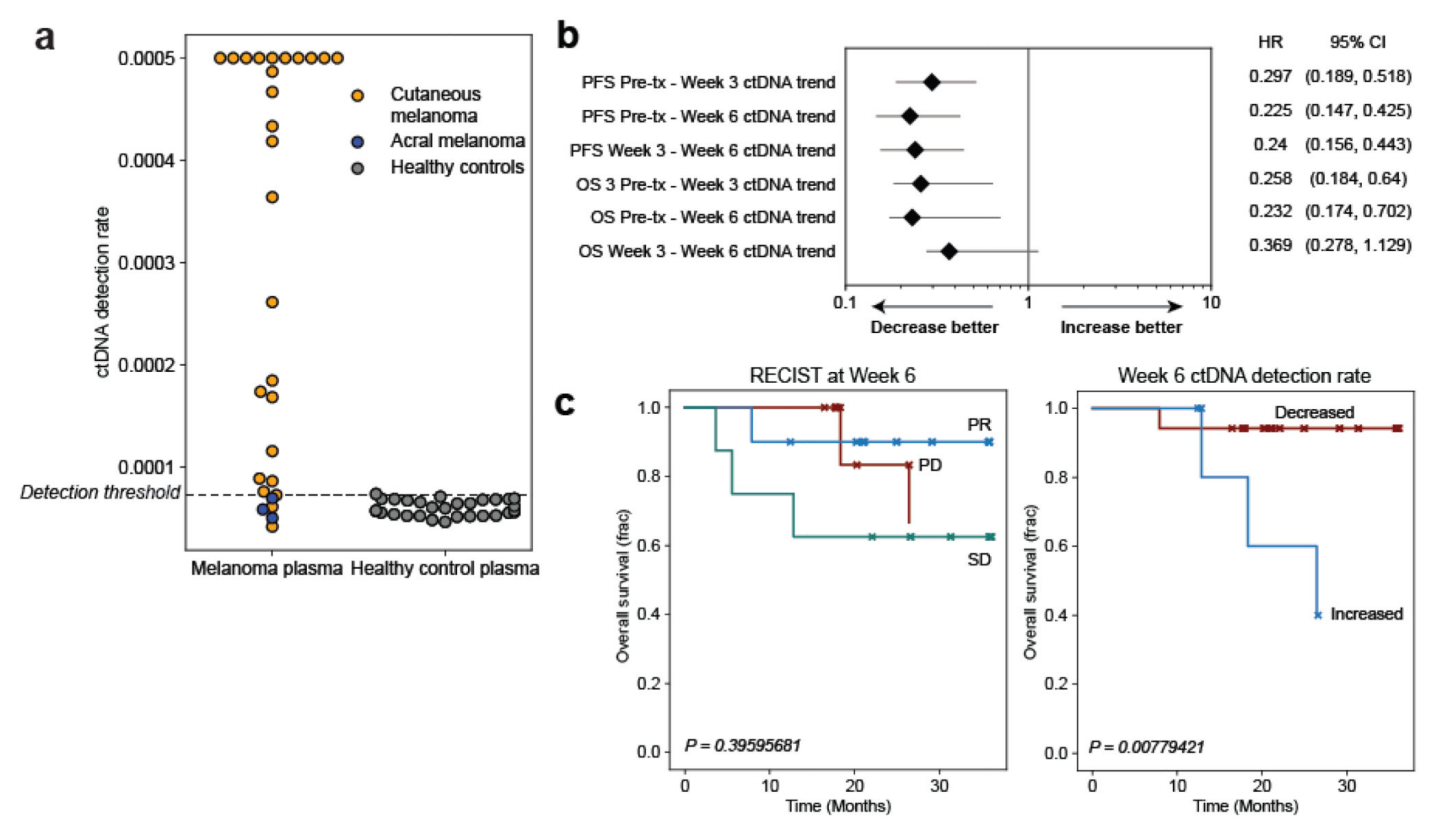
Use of MRD-EDGE^SNV^ in acral melanoma and monitoring response to immunotherapy with MRD-EDGE^SNV^ **a)** ctDNA detection rates for pretreatment cutaneous melanoma samples from the adaptive dosing cohort (*n*=26, orange, detection rate was capped at 0.0005) compared to acral melanoma samples (*n*=3, blue, pre- and posttreatment timepoints from one patient with acral melanoma) sequenced within the same batch and flow cell and detection rates as healthy control plasma (*n*=30, gray). ctDNA is not detected from acral melanoma plasma, demonstrating absence of batch effect and the specificity of MRD-EDGE^SNV^ for the UV signatures associated specifically with cutaneous melanoma. **b)** Forest plot demonstrating relationship between ctDNA TF trend (increase or decrease) and progression-free survival (PFS) and overall survival (OS) at serial posttreatment timepoints. MRD-EDGE^SNV^ TF estimates are measured as a detection rate normalized to the pretreatment sample (normalized detection rate, nDR). Each posttreatment timepoint is prognostic of PFS outcomes. HR, hazard ratio. **c)** (left) Kaplan–Meier overall survival analysis for Week 6 RECIST response (*n*=10 partial response, ‘PR’, *n*=8 stable disease, ‘SD’, *n*=6 progressive disease, ‘PD’) in the adaptive dosing melanoma cohort (*n*=26 patients) where CT imaging was available at Week 6 shows no significant relationship with OS (multivariate logrank test). (right) Kaplan–Meier OS analysis for Week 6 ctDNA trend in adaptive dosing melanoma patients with decreased (*n*=17) or increased (*n*=5) nDR compared to pretreatment timepoint as measured by MRD-EDGE^SNV^. Patients with undetectable pretreatment ctDNA *(n=*2) were excluded from the analysis, as were 2 patients where Week 6 plasma was not available for analysis. Increased nDR at Week 6 was associated with shorter overall survival (two-sided log-rank test).

We evaluated MRD-EDGE^SNV^ performance on samples from patients with advanced cutaneous melanoma treated with combination ICI on The Adaptively Dosed Immunotherapy Trial^52^ (‘adaptive dosing cohort’, *n*=26 patients Fig. 5c). The protocol aimed to spare excess combination ICI treatment by identifying responders through early imaging at Week 6 and transitioning these patients to monotherapy with nivolumab. Plasma-only MRD-EDGE^SNV^ fragment detection rates distinguished pretreatment melanoma samples from non-cancer plasma samples (*n*=30) with an AUC of 0.94 (95% CI: 0.86–1.0, Fig. 5d). In keeping with our tumor-informed analyses, detection threshold was set at a specificity of 95% or greater, yielding sensitivity of 92%. As a negative control, we included pre- and posttreatment plasma from a patient with acral melanoma, a cancer without SBS7 UV light signature, within the same sequencing batch. As expected, we observed no ctDNA detection in these samples (Extended Data Fig. 8a), confirming that our classifier is specific to cutaneous melanoma.

We benchmarked MRD-EDGE^SNV^ ctDNA detection in pretreatment plasma against a targeted panel^7^ with tumor-informed mutation calling covering 129 common cancer genes (‘tumor-informed panel’) in a subset of 14 patients with available samples (Supplementary Table 9). In parallel, results were also compared to the same targeted panel with *de novo* mutation calling (‘*de novo* panel’) and to ichorCNA^53^, an established WGS CNV TF estimator. Among these approaches, ctDNA sensitivity was highest for MRD-EDGE^SNV^ and the tumor-informed panel (Fig. 5e). Comparison of serial samples demonstrate broadly similar trends following ICI treatment between MRD-EDGE^SNV^ and the tumor-informed panel (Fig. 5f, Methods).

Among samples evaluated across platforms (*n*=43 total, 14 pretreatment and 29 post-treatment samples), detection consistency (the agreement between platforms for detected versus undetected ctDNA) was highest between MRD-EDGE^SNV^ and the tumor-informed panel (88%, Fig. 5g), and MRD-EDGE^SNV^ detected the lowest VAF detected by the tumor-informed panel, estimated at 1*10^-4^. To benchmark MRD-EDGE^SNV^ in clinical surveillance, we compared changes in ctDNA TF at Week 6 following ICI treatment and found that MRD-EDGE^SNV^ showed higher agreement with the tumor-informed panel than the *de novo* panel and ichorCNA (Fig. 5h).

### Tracking response to ICI with plasma-only MRD-EDGE^SNV^

**Figure 6:**
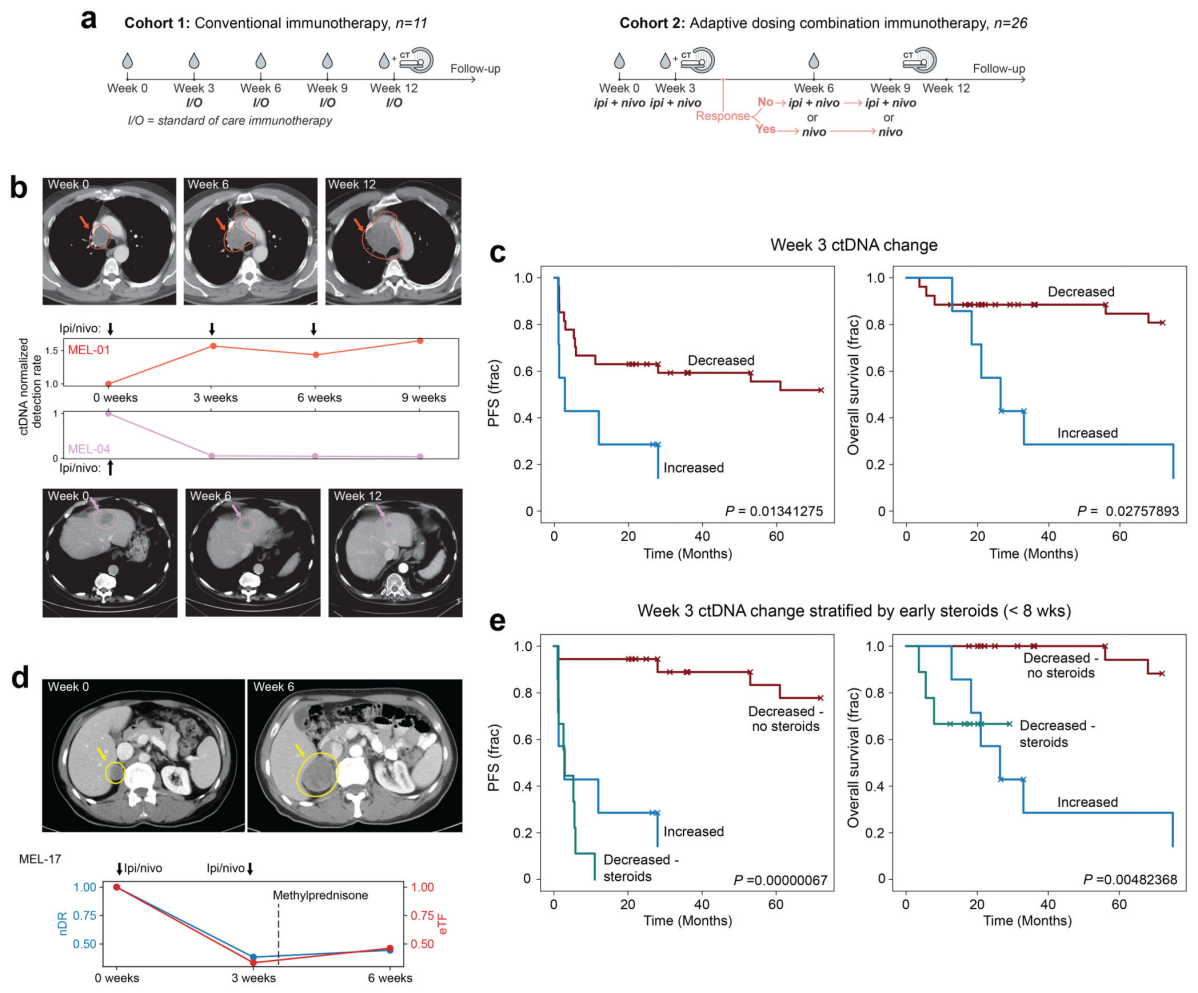
Serial monitoring of clinical response to immunotherapy with MRD-EDGE^SNV^ **a)** Two advanced melanoma cohorts. (left) conventional immunotherapy cohort received nivolumab monotherapy or combination ICI. Plasma was collected at pretreatment timepoint and weeks 3, 6, and 12. Cross sectional imaging to evaluate response to treatment was performed at 12 weeks. (right) adaptive dosing cohort received combination immunotherapy as in Fig. 5c. **b)** Serial plasma TF monitoring with MRD-EDGE^SNV^ corresponds to changes seen on imaging. TF estimates are measured as normalized detection rate (nDR) to the pretreatment sample for MRD-EDGE^SNV^. (top) ctDNA nDR increases over time in a patient with disease refractory to ICI. The patient had progressive disease at Week 6 and Week 12 CT assessment. (bottom) ctDNA nDR decreased at Week 3 in a patient with a partial response to therapy. CT imaging demonstrates tumor shrinkage at Week 6 and Week 12. **c)** Kaplan–Meier progression-free (left) and overall (right) survival analysis for Week 3 ctDNA trend in patients with decreased (*n*=27) or increased (*n*=7) nDR, measured by MRD-EDGE^SNV^. Patients with undetectable pretreatment ctDNA *(n=*3) were excluded. Increased nDR at Week 3 was associated with shorter progression-free and overall survival (two-sided log-rank test). **d)** (top left) pretreatment CT imaging of a patient with decreased ctDNA in response to ICI at Week 3 on both MRD-EDGE^SNV^ (nDR, blue) and a tumor-informed panel (normalized variant allele frequency, nVAF, red). Following the administration of methylprednisone at Week 3, estimated TF (eTF) on both ctDNA detection platforms increased. At Week 6, progressive disease is seen on CT imaging (top right). **e)** Early steroids for immune-related adverse events (irAEs) within the combination ICI dosing period (prior to Week 8) further stratify Week 3 survival analyses. Kaplan–Meier progression-free survival (left) and overall survival (right) analysis for patients with primary refractory disease (‘Increased’, blue, *n*=7), defined as rising nDR seen at Week 3 following first dose of treatment, decreasing ctDNA who did not receive steroids (‘Decreased - no steroids’, red, *n*=18), and patients who received steroids for irAEs within the combination ICI dosing period (‘Decreased - steroids’, green, *n*=9). P value reflects multivariate logrank test.

In advanced melanoma, radiographic imaging may lag ICI response by months, and bespoke panel approaches have shown that liquid biopsy can provide faster response readouts^2,6,51,54,55^. To explore the role of plasma-only MRD-EDGE^SNV^ in ICI response prognostication, we expanded the adaptive dosing melanoma^52^ cohort described above to include additional patients treated with standard of care immunotherapy (‘conventional immunotherapy’, *n*=11 patients, Fig. 6a, Supplementary Table 5). We evaluated the ability of MRD-EDGE^SNV^ to prognosticate clinical outcomes at serial plasma timepoints (122 plasma samples from *n*=37 patients, Supplementary Table 10). Serial cfDNA measurements were normalized to pretreatment levels, and patients with undetected pretreatment ctDNA (*n*=3) were excluded from further clinical analyses. Trends in MRD-EDGE^SNV^ normalized fragment detection rate (nDR, Methods) tracked radiographic imaging results (Fig 6b). We found that decreasing ctDNA TF was associated with longer progression-free survival (PFS) (*P*=0.01) and overall survival (OS) (*P*=0.03, Fig. 6c) as early as at week 3 after the first ICI infusion and at week 6 (Extended Data Fig. 8b). In contrast, CT imaging at week 6, which defines PFS, showed no significant relationship between RECIST response and OS (*P*=0.40, Extended Data Fig. 8c).

We observed several instances where decreasing ctDNA at week 3 was not linked to a durable ICI response. For example, both MRD-EDGE^SNV^ and the tumor-informed panel captured decreasing ctDNA at week 3 in patient MEL-17. However, both platforms found increasing ctDNA between week 3 and week 6 and the patient had progression of disease in the liver on week 6 imaging (Fig. 6d). We reasoned that the high toxicity rate from combination ICI, where nearly 40% of patients will stop treatment early because of immune-related adverse events (irAEs)^56^, may have confounded classification at week 3. Clinically, irAEs are often treated with corticosteroids, and early steroid use (within 8 weeks of ICI treatment) is associated with shorter PFS and OS in melanoma^57^. We therefore stratified our melanoma patients into 3 groups, patients with no ctDNA response (*n*=7), and patients with an initial ctDNA response either treated or untreated with early steroids (*n*=9 and *n*=18, respectively). Here, we observed an association between shorter PFS (*P*=6.7*10^-7^) and OS (*P*=4.8*10^-3^, Fig. 6e) and early steroid administration. Our findings invite further inquiry into how to incorporate ctDNA serial measurement to optimize immunosuppressive treatment in the weeks following ICI initiation.

**Extended Data Fig. 9:**
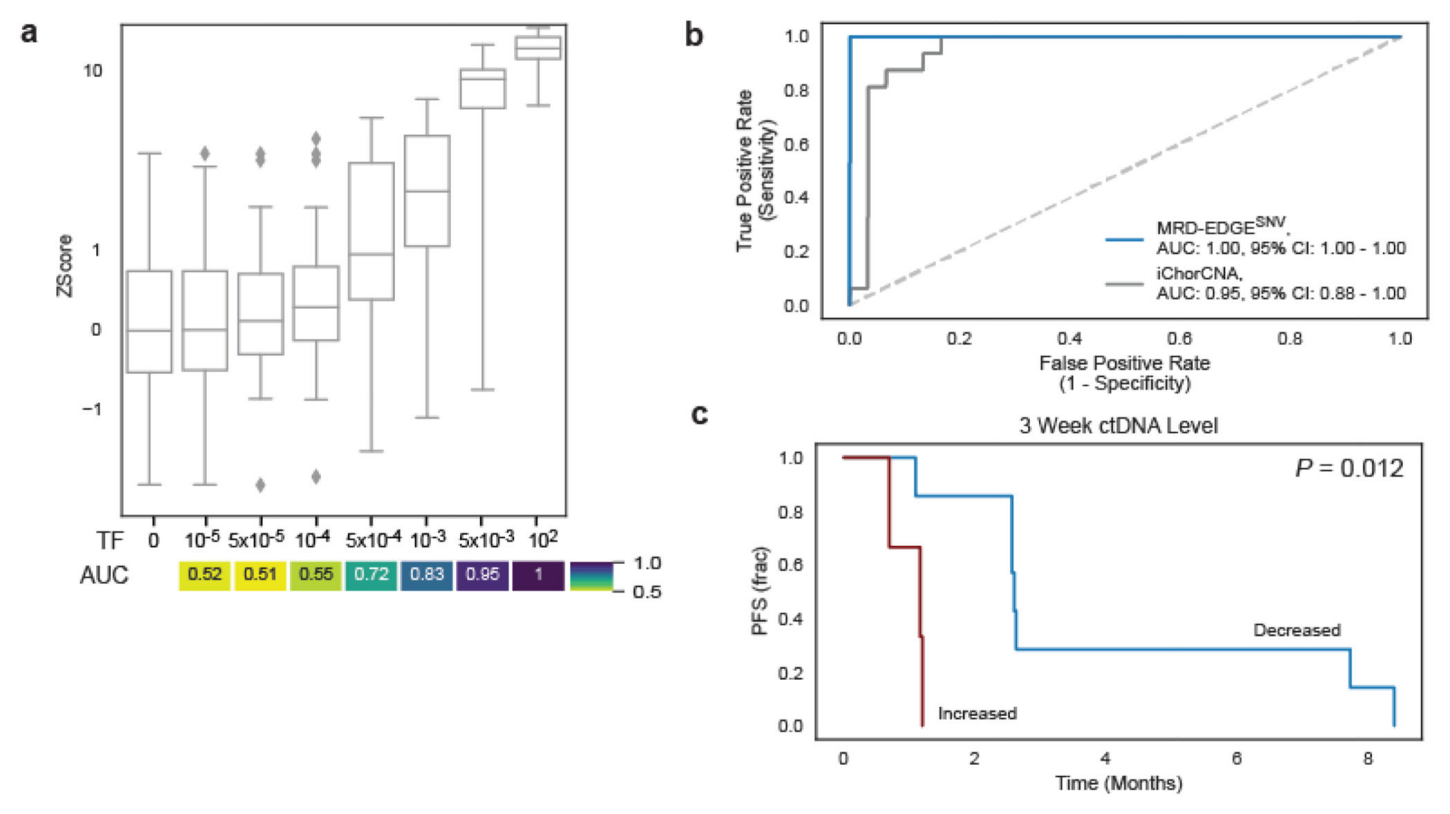
Use of MRD-EDGE^SNV^ to monitor response to ICI in small cell lung cancer **a)** *In silico* studies of cfDNA from the SCLC sample SC-128 (pretreatment TF = 22.9%) mixed in *n*=20 replicates against cfDNA from a healthy plasma sample (TF=0) at mix fractions 10^-5^–10^-2^ at 25X coverage depth. MRD-EDGE^SNV^ enables sensitive detection of TF as low as TF=5*10^-4^ (AUC 0.72), measured by Z score of SNV fragment detection rate against unmixed control plasma (TF=0, n=20 randomly chosen replicates), without matched tumor tissue to guide SNV identification. Box plots represent median, bottom and upper quartiles; whiskers correspond to 1.5 x interquartile range. An AUC heatmap measures detection vs. TF=0 at different mixed TFs. **B)** ROC analysis on detection rates for MRD-EDGE^SNV^ (blue) and TF estimation with ichorCNA (gray) in pretreatment SCLC plasma samples (Supplementary Table 7). Fragment detection rates in SCLC plasma samples (*n*=16 plasma samples, Supplementary Table 5) were compared with fragment detection rates in control plasma samples (*n*=30). **C)** Kaplan–Meier progression-free survival analysis for Week 3 ctDNA trend in SCLC patients with decreased (*n*=7) or increased (*n*=3) normalized detection rate (nDR) as measured by MRD-EDGE^SNV^. Increased nDR at Week 3 was associated with shorter progression-free survival (two-sided log-rank test).

To determine if MRD-EDGE^SNV^ is applicable to other high-mutation burden solid tumors, we applied MRD-EDGE^SNV^ to 16 advanced SCLC patients treated with combination ICI. *In silico* mixing studies demonstrated detection at TF=5*10^-4^ (AUC 0.72, Extended Data Fig. 9a). MRD-EDGE^SNV^ was highly sensitive for pretreatment SCLC ctDNA (Extended Data Fig. 9b, Supplementary Table 10). As in melanoma, increasing ctDNA at week 3, as measured by nDR, was associated with shorter PFS (Extended Data Fig. 9c).

## Discussion

The use of noninvasive liquid biopsy to detect MRD and track response to therapy heralds the next frontier in precision oncology. MRD-EDGE leverages the breadth of plasma WGS to increase liquid biopsy sensitivity. Broadly, MRD-EDGE^SNV^ uses advanced machine learning and a biologically-informed feature space to enrich ctDNA signal. The deep learning SNV architecture in MRD-EDGE^SNV^ provides a flexible platform for integrating disease-specific molecular features, outperforms other machine learning approaches, and demonstrates generalizability across cancer types and sequencing preparations. For CNVs, machine-learning guided signal denoising enables accurate inference of plasma read depth skews, BAF inference expands applicability through incorporation of plasma cnLOH, and fragment length entropy provides an orthogonal metric for CNV assessment. The lower degree of aneuploidy needed for ultrasensitive detection (Fig. 2e) and ability to capture signal from cnLOH will enable application to a diverse set of solid tumors lacking high somatic SNV burden, as demonstrated by our CNV-only analysis of TNBC recurrence after definitive treatment (Fig. 3m-l).

Our simple WGS molecular workflow avoids the clinical complexity of bespoke panels, and the smaller plasma cfDNA input requirements will enhance MRD-EDGE’s translational impact in diverse clinical settings, especially given the rapid decline in sequencing costs^58^. MRD-EDGE enabled the detection of postoperative CRC and TNBC MRD, as well as tracking of plasma TF dynamics in response to neoadjuvant ICI. Further, the unique sensitivity of MRD-EDGE allowed us to examine ctDNA shedding from precancerous colorectal adenomas. While this tumor-informed approach cannot be used for screening, the detection of ctDNA in a substantial proportion of cases argues that ctDNA may be present without invasive disease. ctDNA-guided detection of premalignant lesions is therefore a viable goal, if tools with sufficient sensitivity can be developed for this setting. Finally, we leveraged the enhanced signal-to-noise enrichment of MRD-EDGE to perform plasma-only (non-tumor informed) ctDNA detection in advanced melanoma and SCLC. MRD-EDGE^SNV^ allowed for early and accurate assessment of response to ICI, a challenging clinical setting for prognostication^54,59^.

Collectively, our data support using plasma WGS as a complementary strategy to the prevailing paradigm of ctDNA mutation detection via deep targeted panel sequencing in critical therapeutic contexts. Future large-scale interventional studies will be necessary to demonstrate the value of this approach to inform real-time clinical decision making.

## Methods

### Human subjects and sample processing

This study was approved by the relevant local ethics committees and institutional review boards (IRB), and was conducted in accordance with the Declaration of Helsinki protocol. Blood samples were collected from patient and healthy adult volunteers enrolled in clinical research protocols at NewYork-Presbyterian/Weill Cornell Medical Center, Memorial Sloan Kettering Cancer Center, Massachusetts General Hospital, the Royal Marsden NHS Foundation Trust in the United Kingdom, British Columbia Cancer Center in Canada, or Aarhus University Hospital, Bispebjerg Hospital, Randers Hospital, Herning Hospital, Hvidovre Hospital, and Viborg Hospital in Denmark. Melanoma tumor, normal and plasma samples from the Royal Marsden NHS Foundation Trust were obtained under the ethically approved protocol Melanoma TRACERx (Research Ethics Committee Reference 11/LO/0003). Adenoma and pT1 lesion samples were obtained under the ethically approved Endoscopy III protocol H-4-2013-050. Tumor tissues were collected from biopsied or resected lung, melanoma, colorectal, triple negative breast cancer, and adenoma specimens. Cutaneous melanoma, NSCLC, CRC, TNBC, adenoma, and SCLC were diagnosed according to World Health Organization criteria and confirmed in all cases by an independent pathology review. Informed consent on IRB-approved protocols for genomic sequencing of patients’ samples was obtained before the initiation of sequencing studies. Sex was self-reported. Participants did not receive compensation for participation.

### Germline and tumor DNA processing

Tumor tissue and matched germline DNA from PBMCs or adjacent normal tissue were collected and stored at −80 °C until they were processed for extraction. Genomic DNA was extracted from tumor tissue using the QIAamp DNA Mini Kit (Qiagen). Genomic DNA was extracted from PBMCs using the QIAamp DNA Blood Kit (Qiagen). Libraries were prepared using either TruSeq DNA PCR-Free Library Preparation Kit (Illumina) or Agilent Sure Select (Supplementary Table 11 and Supplementary Table 12). Input was 1 μg of DNA per the recommended protocol^63^, with minor modifications as described below. Intact genomic DNA was concentration normalized and sheared using the Covaris LE220 sonicator to a target size of 450 bp. After cleanup and end repair, an additional double-sided bead-based size selection was added to produce sequencing libraries with highly consistent insert sizes. This was followed by A-tailing, ligation of Illumina DNA Adapter Plate adapters and two post-ligation bead-based library cleanups. These stringent cleanups resulted in a narrow library size distribution and the removal of remaining unligated adapters. Final libraries were run on a Fragment Analyzer (Agilent) to assess their size distribution and quantified by qPCR with adapter-specific primers (Kapa Biosystems). Libraries were pooled together based on expected final coverage and sequenced across multiple flow cell lanes to reduce the effect of lane-to-lane variations in yield. WGS was performed on the HiSeq X (HCS HD 3.5.0.7; RTA v2.7.7) or NovaSeq 6000 (Illumina) at 2 x 150-bp read length, using SBS v3 (Supplementary Table 11).

### Plasma DNA processing

On the same day of blood collection, blood collection tubes (Streck or K2-EDTA, Supplementary Table 5) were centrifuged at 2,000 r.p.m. for 10 min to separate plasma. cfDNA was then extracted from human blood plasma by using the Mag-Bind cfDNA Kit (Omega Bio-Tek). The protocol was optimized and modified to optimize yield^14^. Elution time was increased to 20 min on a thermomixer at 1,600 r.p.m. at room temperature and eluted in 35-μl elution buffer. The concentration of the samples was quantified by a Qubit Fluorometer (Thermo Fisher), and samples were run on a fragment analyzer by using the High Sensitivity NGS Fragment Analysis Kit (Agilent) to define the size of cfDNA extracted and genomic DNA contamination. For plasma samples with significant genomic DNA contamination (fragment size > 240 base pairs for more than 20% of fragments at library preparation in tape station analysis), we performed a 0.4x cleanup using SPRIselect magnetic beads (Beckman Coulter) on the extracted cfDNA. Samples that underwent bead cleaning are listed in Supplementary Table 5. Bead cleanup did not change fragment insert size distributions in affected samples (Supplementary Fig. 10).

A subset of plasma samples was sequenced at Aarhus University in Denmark (Supplementary Table 5). For these samples, cfDNA was extracted from human blood plasma using the QIAmp Circulating Nucleic Acids kit (Qiagen) and eluted in 60 μl elution buffer (10 mM Tris-Cl, pH 8.5). The concentration of the samples was quantified by droplet digital PCR (ddPCR, Bio-Rad Laboratories), using assays specific to two highly conserved regions on Chr3 and Chr7, as previously described^64^. In addition, all samples were screened for contamination of genomic DNA from leukocytes using a ddPCR assay targeting the VDJ rearranged IGH locus specific for B cells, as previously described^64^. No samples were contaminated by genomic DNA from leukocytes.

### Plasma cfDNA library preparation and sequencing

Samples sequenced at the New York Genome Center and the British Columbia Cancer Center were processed using KAPA Hyper Library Preparation. Cohorts included in Zviran et al. were processed as previously described^14^. Samples with a mass above 5 ng were prepared for next-generation sequencing on Illumina’s HiSeq X or NovaSeq by using a modified manufacturer’s protocol. The protocol was scaled down to half reaction by using 25μl of extracted cfDNA. IDT for Illumina TruSeq Unique Dual Indexes^63^ was used by diluting 1:15 with EB (elution buffer), and ligation reaction was adjusted to 30 minutes. Additional 0.8x SPRIselect magnetic bead (Beckman Coulter) cleanup was included after post-ligation cleanup to remove excess adapters and adapter dimers. cfDNA from 1 mL of plasma was used for all of the plasma samples in this study. For samples with low concentration, an additional 1 ml of plasma was extracted, and the DNA aliquot with the highest mass was used for library preparation. The number of PCR cycles was dependent on initial cfDNA total mass. For samples with more than 5 ng of total cfDNA, 5-7 PCR cycles were performed. For samples with less than 5 ng of total cfDNA, 7–10 PCR cycles were performed (Supplementary Table 5). Quality metrics were performed on the libraries by Qubit Fluorometer, High Sensitivity DNA Analysis Kit and KAPA SYBR FAST qPCR Kit (Roche). WGS was performed on the HiSeq X (HCS HD 3.5.0.7; RTA v2.7.7) at 2 × 150-bp read length or NovaSeq 6000 at 2 x 150-bp read length (Supplementary Table 5) to a target depth of 30x.

At Aarhus University, cfDNA from 2mL plasma (see Supplementary Table 5 for DNA mass) was used as input for library preparation using a modified manufacturer’s protocol. xGen UDI-UMI Adapters were used and the ligation reaction was adjusted to 30 minutes. Agencourt AMPure XP beads (Beckman Coulter) were used for both cleanup steps with a bead:DNA ratio of 1.2x and 1.0x for the post-ligation and post-PCR cleanup, respectively. The number of PCR cycles was 7 for all cfDNA samples. Qubit Fluorometer and TapeStation D1000 were used for library quality control. WGS was performed on NovaSeq 6000 at 2 x 150-bp read length to a target depth of 30x.

### Preprocessing, quality control analysis and sample identification and concordance

WGS reads for primary tumor, matched germline and plasma samples were demultiplexed using Illumina’s bcl2fastq (v2.17.1.14) to generate FASTQ files. The primary tumor and matched germline WGS were submitted to the New York Genome Center somatic preprocessing pipeline, which includes alignment to the GRCh38 reference (1000 Genomes version) with BWA-MEM (v0.7.15)^65^. For plasma cfDNA, we used a modified alignment pipeline to accommodate adapter trimming after observing increased adapter contaminated reads in cfDNA samples compared with tumor samples, given that cfDNA has shorter fragment size, which can lead to R1 and R2 overhang. We therefore used Skewer^66^ for adapter trimming (default settings) and subsequently aligned samples using BWA-MEM (default settings) to the GRCh38 reference (1000 Genomes version). For all samples, duplicate marking and sorting was done using NovoSort MarkDuplicates (v3.08.02), a multi-threaded bam sort/merge tool by Novocraft Technologies; http://www.novocraft.com), followed by indel realignment (performed jointly for the tumor and matched germline) and base quality score recalibration using GATK (v4.1.8; https://software.broadinstitute.org/gatk), resulting in a final coordinate sorted bam file per sample. Alignment quality metrics were computed using Picard (v2.23.6; QualityScoreDistribution, MeanQualityByCycle, CollectBaseDistributionByCycle, CollectAlignmentSummaryMetrics, CollectInsertSizeMetrics, CollectGcBiasMetrics) and GATK (v4.1.8; average coverage, percentage of mapped and duplicate reads). To specifically assess for sample contamination, we applied Conpair^67^ (v.0.2), which validated genetic concordance among the matched germline, tumor and plasma samples, as well as evaluated any inter-individual contamination in the samples. Samples that showed low concordance (<0.99) were excluded from further analysis. Specifically, one set of serially monitored cutaneous melanoma samples from the melanoma patient MEL-155 was rejected from analysis due to low concordance score (Supplementary Table 5).

### Tumor / Normal somatic mutation calling

To achieve stringent somatic variant calling, we enforced high-confidence SNV calls according to published methods from our center^68^. The tumor and normal bam files were processed through NYGC’s variant calling pipeline which consists of MuTect2 (GATK v4.0.5.1), Strelka2 (v2.9.3) and Lancet (v1.0.7) for calling SNVs. High confidence SNV calls were defined as those that were called by two or more variant callers. We further excluded variants that were present at any allelic fraction in the matched normal sample.

To identify SNVs for colorectal adenomas and pT1 lesions within formalin-fixed paraffin-embedded (FFPE) tumor tissue, we used univariate Gaussian mixture models (GMM, sklearn.mixture) with the underlying assumption that FFPE artifactual noise SNVs and true SNVs can be expressed as a mixture of Gaussian densities according to VAF (low VAF for artifactual noise and high VAF for true tumor mutations). For each FFPE tumor sample, we set a VAF threshold at a 10% false positive rate according to the GMM, and only included SNVs with VAFs above this threshold.

CNVs, including deletions, amplifications and copy-neutral LOH, were called using Sequenza (v3.0.0)^69^. We only considered CNVs in autosomal regions (chr1-22) of the genome where the size of the CNV was greater than 1.5 Mb. Segments with Depth Ratio of 1 (Depth Ratio 0.8-1.2) were characterized as neutral while those with Depth Ratio >1.2) were selected as amplifications, and Depth Ratios < 0.8 were selected as deletions. Copy neutral LOH segments were selected when Minor Copy-number was assigned 0 by Sequenza.

Sequenza required >15% tumor purity for tumor-informed BAF and CNV calling at WGS sequencing depths of 20-80X, as used in this study. A subset of NSCLC CNVs was called with ichorCNA (Supplementary Table 11) as low tumor purity precluded accurate CNV calls with Sequenza. In this low purity setting, CNV calls were quality filtered using tumor BAF and read depth ratio to separate aneuploidy from artifact.

### Tumor-informed plasma cfDNA SNV identification

Detection of patient-specific SNV profiles was performed by searching the plasma WGS for all sites from the matched tumor SNV profile with corresponding mutations in the same genomic site and the same substitution. To efficiently identify variants present in the sequencing data, we used a custom Python script (Python version 3.6.8), which uses the pysam (v0.15.2) module to efficiently extract alignments harboring variants and extracted any read that both uniquely maps to a variant of interest and was in an aligned portion of the read (no clipping or soft masking at the position of the variant).

### Plasma and tumor recurrent artifact, germline, and regional filters

In all plasma samples, we removed artifactual variants using a local recurrent artifact plasma ‘blacklist’ filter generated by aggregating pileup SNVs within our plasma WGS and tumor WGS databases. We then counted individual SNVs within all pileups, excluding recurrent SNVs in samples from the same patient. For tumor-informed analyses, both plasma and tumor recurrent artifact filters were applied, while for plasma-only analyses, only plasma blacklists were applied. Use of cohort-specific blacklists is summarized in Supplementary Table 13. To further exclude potential germline variants, we used the gnomAD database (version 3.0) which contains genetic variants from >70,000 whole genomes^70^. We downloaded the gnomAD version 3.0 variant call format (VCF) file that was available in hg38 coordinates from the gnomAD browser. We annotated single base changes that we identified with their population allele frequency and removed any candidate variants that were present in gnomAD with an allele frequency > 1/100. Finally, we excluded variants from simple repeat regions and centromeres from a problematic region blacklist^71^.

### Construction of ctDNA SNV training sets

Training sets consisted of ctDNA SNV fragments (true label) from plasma samples with high ctDNA burden from patients with metastatic disease and cfDNA variant containing fragments (false label) from healthy controls without known cancer, processed in the same location and sequenced under the same settings. Supplementary Table 1 lists samples used in training for NSCLC, CRC, and melanoma. These plasma samples were drawn from patients with high TF, advanced disease and were not included in any downstream MRD-EDGE^SNV^ clinical application such as ctDNA detection from early-stage CRC or NSCLC plasma (Fig. 3).

Prior to fragment classifier training, we first implemented quality filters to filter low-quality noise, germline SNPs, and genomic DNA contamination (see Supplementary Table 2 for quality filters by model type). Filters removed SNV fragments with low base quality (<25 on Phred scale) or low depth (<10 supporting reads), and removed fragments with insert sizes outside of a 40 bp – 240 bp range to reduce genomic DNA contamination. Germline variants were excluded by filtering high VAF variants (VAF > 0.2), except in cases where estimated ichorCNA TF was > 0.2. In *plasma-only* settings, only candidate variants found on overlapping paired reads (R1 and R2 concordant) were retained.

To maximize the accuracy of true (positive) labels, we implemented the following strategies to limit noise contamination in our ctDNA (true label) SNV fragment sets. In all true label settings, we used training samples from patients with high burden metastatic disease (TF 9-24% as called by ichorCNA, Supplementary Table 1). In samples with matched tumor tissue, we identified ctDNA SNVs by intersecting tumor high confidence somatic calls from the NYGC Somatic Pipeline^68^ with SNVs in plasma. When matched tumor tissue was not available, we called mutations directly in the plasma against normal germline samples using Mutect2^72^, leveraging the high TF in these samples to identify consensus somatic mutations (Supplementary Table 1). To further filter noise, when possible, we used the intersection of ctDNA SNV fragments from two high TF timepoints from the same patient (Supplementary Table 1).

To identify cfDNA SNV artifacts for our false labels, we identified all fragments with a SNV against the reference genome through samtools (v.3.1) mpileup. Fragments were then subjected to the above quality filters, including VAF filter and recurrent artifact filter. After filtering, remaining SNV fragments were randomly sampled to select the quantity required to match the number of positive label fragments for each model training set in Supplementary Table 1.

### Construction of SNV feature space

Feature evaluation was performed on high quality SNV fragments that passed initial quality filters (Supplementary Table 2, see **Construction of ctDNA SNV training sets**). To preclude batch effects from sample mixing in this analysis, our positive label ctDNA SNV fragments (see **Tumor / Normal somatic mutation calling**) were compared to negative label cfDNA SNV artifacts drawn from the same plasma sample. For example, ctDNA fragments from the NSCLC sample NSCLC-206 were compared to cfDNA SNV artifacts from NSCLC-206, meaning inferences on feature predictive power cannot be attributed to plasma sample quality or sequencing batch effects. To measure the individual contribution of candidate features, we assessed svROC (single variable area under the receiver operating curve), a measure of how well each individual feature separates ctDNA (true label) from cfDNA artifacts (false label). For example, svROC for the feature fragment length represents the AUC of the fragment length size for discriminating ctDNA SNV fragments from cfDNA SNV artifacts. For categorical features, AUC is assessed on a held-out validation set of fragments after a linear classifier was trained to predict positive or negative label based on one-hot encoded categorical features. Features and corresponding svROC scores are reported in Supplementary Table 2.

We implemented several strategies to create tissue-specific regional features that could inform the regional likelihood of somatic mutagenesis. For each candidate feature, quantitative values were calculated in a sliding interval window around every individual SNV fragment. The size of this window was optimized by comparing the correlation between feature and label between true and false label SNVs from our training set. Window sizes are reported in Supplementary Table 2. Quantitative features were min / max normalized to values between 0 and 1.

To evaluate local tumor mutational density, we aggregated WGS SNV mutation calls from the PCAWG database^60^ and counted the aggregate number of SNV mutations across all available tumor samples in a specific primary disease (melanoma, NSCLC or CRC). Local transcription factor and histone ChIP-Seq marks as well as tissue-specific bulk RNA expression values were calculated as reads per kilobase per million mapped reads (RPKM) and were obtained from primary tissue alignments in ENCODE^73^. For each feature category (e.g., H3K4me3 ChIP-Seq marks), we assessed all alignments in ENCODE and selected those with the highest Pearson correlation between training set true and false label SNVs on Chromosomes 1-10. Regional DNase peaks were downloaded as narrowpeak files from ENCODE^73,74^ and lifted to GRCh38. Disease-specific ATAC peak calls were downloaded from TCGA^61^. Plasma WGS sequencing error density was calculated by aggregating all SNV pileup variants from non-cancer control plasma sequenced at the New York Genome Center. ChromHMM^62^ chromatin annotation tracks were downloaded from ENCODE and lifted to GRCh38. Hi-C compartment information was drawn from Hi-C SNIPER^75^ bed files. Replication timing and mean expression values were taken from prior work^25^ and lifted to GRCh38. Supplementary Table 2 lists features used in each model type.

### SNV deep learning model architecture and model training

To evaluate SNV fragments with our MRD-EDGE^SNV^ fragment classifier, candidate SNV fragments were pulled from alignment files using pysam (v0.15.2) and salient features were encoded as input to our deep learning model architecture (Fig. 1d) with a custom Python (v3.6.8) script. There are two main components of our deep learning SNV fragment classifier: a regional MLP, and a fragment CNN. The MLP takes a tabular feature representation as input and consists of five fully-connected layers with ReLU activation functions of decreasing size. Each layer is preceded by a batch normalization layer and followed by a dropout layer (with the exception of dropout following the final layer).

We chose to represent cfDNA fragments as an 18x240 tensor (Fig. 1d). Within the rows of the tensor, we compared the one-hot encoded reference sequence to the R1 and R2 sequence of a cfDNA fragment containing a variant (either true somatic mutation or sequencing artifact). We also encoded the length and position of R1 and R2, and we marked the position of the SNV to be classified as ctDNA or noise. The columns of the matrix mark individual nucleotides along the length of the fragment. The R1 and R2 regions were padded with neutral values (0.2 in each of the 5 possible nucleotides N, A, C, T, G) where the read does not overlap the reference sequence. This tensor serves as input to a CNN which consists of 4 one-dimensional convolution layers (convolving over the base pair width dimension), each followed by a max pooling operation. This is then followed by three fully-connected layers (with ReLU activation) and a subsequent dropout layer, and ends with a single sigmoid-activated fully-connected layer (parallel to the MLP). Model architectures were built in Keras (v.2.3.0) with a Tensorflow base (1.14.0). The fragment tensor has potential access to features including fragment length; key genomic features including mutation type, trinucleotide context, and leading or lagging strand; and quality metrics such as PIR and edit distance (how many variants against the reference sequence are present in a fragment). The tensor structure was coded to account for all possible CIGAR outputs, including insertions, deletions, skips, and soft masks, by inserting ‘N’ (base undetermined) values in reads (deletions, soft skips, soft masks) or the reference sequence and as needed in the alternate read (insertions).

Finally, to integrate fragment and regional information, an ensemble classifier with sigmoid activation jointly evaluated the latent space outputs from both the fragment CNN and regional MLP to generate a score between 0 and 1, reflecting the model-based likelihood that a candidate variant containing cfDNA fragment harbored a true somatic mutation (1) vs. a sequencing artifact (0).

We trained our deep learning classifiers (melanoma, CRC, NSCLC) using Keras with tensorflow base on randomly chosen fragments from our disease-specific training sets (NSCLC, CRC, and melanoma, Supplementary Table 1). Validation and test sets were held out from training and drawn from separate patient samples. All performance metrics, including F1, AUC and accuracy within balanced sets, are reported for train, validation, and test sets (Supplementary Table 1).

Our models were constructed for specific use in the tumor-informed or *de novo* mutation calling setting. In the tumor-informed setting, to harvest more candidate SNVs overlapping tumor SNV loci, we allowed mutations to be present on R1, R2, or on both R1 and R2 (paired read concordance). In the *de novo* mutation calling setting, we enforced paired read concordance as an additional quality filter to reduce sequencing artifacts. This resulted in the application of two lung cancer models: tumor-informed NSCLC and *de novo* SCLC. The models share the same feature space and training samples, and the sole difference between them is the use of paired read concordance as an additional quality filter in the *de novo* setting. All reads with discordant SNVs were excluded.

### Comparison of MRD-EDGE^SNV^ deep learning classifier performance to other machine learning models

The MRD-EDGE^SNV^ ensemble classifier (Fig. 1d) was compared to its individual components (fragment CNN and regional MLP) and other machine learning architectures (MLP random forest, and gradient boosting model) by randomly subsampling without replacement in ten parts ctDNA and cfDNA SNV fragments from the held-out melanoma validation set (Supplementary Table 1) and assessing F1 performance on each subsampling set (Extended Data Fig. 1b). To assess fragment-level features in the random forest, gradient boosting, and MLP models, salient features were encoded as tabular values, including one-hot categorical encodings for trinucleotide context and mutation type of the candidate SNV as well as numerical representation of fragment-length, position of the variant within the read (PIR), read 1 length, and read 2 length. For both gradient boosting and random forest classifiers, we performed a grid search across the space of feature count (1-5) and number of trees (1-20), nominating an optimal parameter choice for maximizing AUC performance on our validation set. The MLP for Fragment + Regional Features has the same architecture as the Regional MLP (see **SNV deep learning model architecture and model training**). The Random Forest Fragment + Regional Features model and the Gradient Boosting Classifier Fragment + Regional Features model were constructed using the Python (version 3.6.8) module sklearn sklearn.ensemble.RandomForestClassifier and sklearn.ensemble.GradientBoostingClassifier, respectively, with default settings. The computational time for model training for each approach is reported in Supplementary Table 3.

Model performance was evaluated at the sample level for the training sample MEL-05_B and the held-out validation sample MEL-100, and held-out test sample MEL-137_B against SNV fragments from non-cancer plasma (control samples evaluated are in Supplementary Table 1). Results are reported in Supplementary Table 3. The MRD-EDGE^SNV^ classifier had the highest F1 and AUC in held-out validation and test sets of all methods evaluated.

### Generation of *in silico* plasma DNA admixtures

See Supplementary Table 4 for samples and metrics. In each study, a high TF sample is paired with a non-cancer plasma sample from the same sequencing center and sequencing platform to remove cohort-specific biases in mixing. Coverage depth is dictated by the underlying coverage of the cancer and non-cancer plasma samples (Supplementary Table 5). In each study, *mu* and *sigma* for Z scores are derived from ctDNA detection rates in the set of unmixed control plasma (TF=0) replicates (see **Plasma SNV-based ctDNA detection and quantification in the tumor-informed approach** and **Evaluating SNVs for *de novo* mutation calling in MRD-EDGE**^**SNV**^.)

For the MRD-EDGE^CNV^ BAF classifer, given the challenges of applying LOH-based classification on samples with different germline SNPs, we generated in silico dilutions, with varying fractions (range 10^-6^–10^-3^), of reads from a pretreatment high burden melanoma plasma sample (MEL-12, pretreatment timepoint, TF 17%, with 1.6 GB of aneuploidy) into a posttreatment plasma sample from the same patient following a major response to immunotherapy (MEL-12 Week 6 timepoint, TF <5% without observable aneuploidy).

SAMtools (v1.1, view -s and merge commands) was used to downsample and admix high-burden cancer plasma cfDNA reads into low-burden or healthy control plasma cfDNA reads accounting for TF and tumor ploidy.

The downsampling ratio S to generate dilutions at various TFs was described previously^14^ and is as follows:

Eq. 1 $$S=\frac{TF_{required\;}}{H_{TF}}=TF_{required\;}*\frac{H_{TF}*P_{L}+\left(1-H_{TF}\right)*2}{H_{TF}*P_{L}}$$

Where H_TF_ denotes ctDNA TF in the high burden cfDNA sample, P_L_ denotes ploidy in the tumor sample. High burden and control coverage is scaled followed by merging of reads:

Eq. 2 $$\begin{array}{l} \;high\;burden\;read\;ratio\;=S*\frac{cov_{req\;}}{cov_{H}}\\ \;control\;read\;ratio\;=(1-S)*\frac{cov_{req\;}}{cov_{C}} \end{array}$$

Where cov_req_ is the required read depth coverage for the admixture sample and cov_H_, cov_C_ are the read depth coverage of the high burden and control samples, respectively. Each study was performed using independent technical replicates, and *mu* and *sigma* for Z scores are derived from the set of unmixed control plasma (TF=0) replicates. Z scores are derived from summed read-depth skews for read depth classifier, BAF score for BAF classifier, and summed fragment length entropy for fragment length entropy classifier (see **Plasma read depth denoising, Evaluation of B-allele frequency in plasma**, and **Evaluation of tumor-informed fragment-length entropy**).

### Generation of experimental plasma DNA admixtures

For synthetic MRD-EDGE^SNV^ performance evaluations, we generated synthetic admixtures (range, 10^-6^–10^-3^) from pretreatment plasma from the melanoma patient MEL-137 mixed with expired plasma from a plasma donor without known cancer (Plasma Bag-01). Initial TF estimation from MEL-137 (TF 13%) was drawn from ichorCNA^53^ and diluted in expired plasma harvested from a single plasma donor without known cancer to form two 1:10^-3^ admixtures in duplicates. Plasma samples were serially diluted in duplicates to create 1*10^-4^, 1*10^-5^, and 1*10^-6^ mix fractions (Supplementary Table 4). To form a noise distribution for ctDNA detection, TF=0 samples were downsampled to 90% coverage to form 15 independent replicates (n=30 replicates in noise distribution, 15 downsampled alignment files from 2 TF=0 replicates). Positive ctDNA detection was defined as MRD-EDGE^SNV^ or MRDetect^SNV^ Z score above 95% specificity against this noise distribution for each platform (see **Plasma SNV-based ctDNA detection and quantification in the tumor-informed approach**).

### Plasma SNV-based ctDNA detection and quantification in the tumor-informed approach

As described previously^14^, we modeled the relationship between coverage, mutation load (SNV/tumor), number of detected variants in cfDNA WGS, and the tumor fraction according to the following equation:

Eq. 3 $$M=N\left(1-{\left(1-TF\right)}^{cov}\right)+\mu *R$$

Where *M* denotes the number of SNVs detected in the plasma sample, *N* denotes the number of SNVs (mutation load) in the patient-specific mutation profile, *TF* denotes the tumor fraction, *cov* denotes the mean coverage depth of the sample (used to approximate the number of opportunities to detect a given variant aggregated across all SNVs in the sample), *μ* denotes the mean noise rate (number of_errors/number of reads evaluated) that corresponds to the patient-specific SNV profile evaluated in control plasma WGS data (see below), and *R* denotes the total number of reads covering the patient-specific mutation profile. This relationship allows the calculation of the plasma TF from the mutation detection rate, even in extremely low allele fraction where the mutation allele fraction itself is not informative (random sampling between 0 and 1 supporting read at best).

To address variation in sequencing artifact noise (*μ*) across patients with different mutation profiles, we apply the patient-specific mutation profile to calculate the expected noise distribution across the cohort of control plasma samples. This process is performed to detect the patient-specific SNVs in control plasma samples or other patients (cross-patient analysis). These detections represent the background noise model for which we calculate the mean and standard-deviation (μ,σ) of artifactual mutation detection rate. Confident ctDNA detection can then be defined by converting the patient-specific detection rate (*det_rate* = number of SNVs detected in cfDNA/number of reads checked = M/R) to a $\;\text{Z}-\text{score}\;=\frac{\;det\_rate-\;\mu }{\sigma }$ , and define a threshold that will keep the specificity above 95%. Specificity and sensitivity performance values were further validated using ROC analyses using the Python (version 3.6.8) module sklearn sklearn.metrics.roc_curve.

The patient TF was then calculated based on point mutation detection using the following equation (which is an inversion of Eq.3), as described previously^14^:

Eq. 4 $$TF=1-{\left(1-\frac{\left[M-\mu *R\right]}{N}\right)}^{\frac{1}{cov}}$$

Where *M* denotes the number of SNVs detected in the plasma sample, *N* denotes the number of SNVs (mutation load) in the patient-specific mutation profile, *TF* denotes the tumor fraction, *cov* denotes the local coverage in sites with a tumor-specific SNV, *μ* denotes the noise rate (number of errors/number of reads evaluated) that corresponds to the patient-specific SNV profile, and R denotes the total number of reads covering the patient-specific mutation profile.

In preoperative plasma samples, MRD-EDGE^SNV^ and MRD-EDGE^CNV^ Z scores are evaluated independently and are summed via Stouffer’s method to form a composite MRD-EDGE detection Z score. In postoperative plasma samples, we define MRD as ctDNA detection with either the MRD-EDGE^SNV^ or MRD-EDGE^CNV^ classifier, in keeping with our prior work^14^. We used this approach to maximize MRD sensitivity, as we view a role for our classifier in de-escalation from adjuvant therapy, which requires optimal sensitivity to justify withholding standard-of-care treatment. This approach further allows us to optimize the most salient features of each tumor type for classification (e.g., MRD-EDGE^CNV^ will be most useful for detecting plasma samples from highly aneuploid tumors, while MRD-EDGE^SNV^ will function optimally in plasma samples from tumors with high mutational burden).

### Selection of control plasma samples for tumor-informed approaches

In the tumor-informed setting, patient-specific mutation profiles are applied to both matched plasma and control plasma. To exclude batch-specific biases, we used control plasma samples obtained from the same collection site, sequencing platform and sequencing location as our cancer plasma samples. For example, our HiSeq CRC plasma, sequenced at the New York Genome Center on Illumina HiSeq X, was compared to similarly sequenced healthy control plasma (HiSeq Controls, Supplementary Table 5), while NovaSeq stage III perioperative CRC cohort, sequenced with Illumina NovaSeq 1.5 at Aarhus University in Denmark, was compared to healthy control plasma sourced and sequenced from that institution (Aarhus Controls). Control plasma samples used in model training or to construct a read depth classifier PON were not used in downstream clinical analyses (Supplementary Table 14).

### Cross-patient analysis

To address potential batch confounding between cancer samples and non-cancer controls sequenced in different batches, we performed cross patient analyses in which we apply the patient-specific mutation profile to calculate the expected noise distribution across the cohort of different patients plasma samples sequenced in the same batch. This is enabled by the low rate of shared variation between any two tumor VCFs in WGS cohorts (Supplementary Fig. 11), consistent with expectations from other WGS datasets^60^. Thus, the unmatched plasma from other cancer patients is an effective control. The cross-patient analysis is otherwise entirely consistent with the control plasma approach detailed above in **Plasma SNV-based ctDNA detection and quantification in the tumor-informed approach**.

### Plasma read depth denoising

We recently introduced a read depth denoising approach for reducing recurrent noise and bias for WGS-based tumor CNV detection^30^. Our read depth pipeline separates foreground (CNV signal) from background (technical and biological bias) in read depth data by learning a low rank subspace across a panel of normal samples (PON) using robust Principal Component Analysis (rPCA) and applies this subspace to a tumor sample to infer CNV events. To optimize our approach for plasma, we first created PONs from healthy control plasma generated with the same sequencing preparation (see **Selection of control plasma for tumor-informed approaches**, Supplementary Table 15). We then created sample-wide median-normalized read depths across the PON for each sample within 10-kb genomic windows. Loess regression was applied at the sample level to account for GC bias^76^. We performed a window-based rPCA decomposition on our PON to yield a subspace of biases that define “background” noise. Cancer plasma samples were subsequently projected on this background subspace to produce two vectors: a background bias projection and a residual corresponding to plasma CNV read depth skews.

To generate sample read depth scores for our read depth classifier, we median-normalized window-level GC-corrected read depth values. We then aggregated this signal based on the direction of the CNV change in tumor (-1 * deletion and +1 * amplification) to produce a mean per-window read depth score as described previously^14^. This sample-level read depth score was compared to read depth scores from held-out control plasma samples in matched genomic regions to generate a final sample-level Z score.

### Evaluation of B-allele frequency in plasma

We applied GATK (v4.1.8, https://software.broadinstitute.org/gatk) HaplotyeCaller to identify genome-wide germline SNPs in PBMC WGS data (cancer patients) and plasma WGS data (non-cancer controls). Plasma and PBMC allelic imbalance may be biased due to allele-specific read-mapping^77^ which may confound inference of allelic imbalance. We applied WASP (v0.3.4, https://github.com/bmvdgeijn/WASP) to filter heterozygous SNP reads that map to an alternative location in the genome when one genotype is permutated to the other. In PBMC, SNPs sites that exhibit mapping bias (e.g., a SNP site with one or more reads in which permutation of the SNP allele causes the read to map to an alternative location) are removed.

Matched tumor tissue was used to identify regions of LOH and to identify the major allele, defined as the allele with highest read count in the tumor, at each SNP site (see **Tumor / Normal somatic mutation calling**). In control plasma, where matched tumor tissue is unavailable, we chose the major allele randomly. To ensure that we evaluated only true SNPs and that our signal was not biased by coverage or subtle clonal mosaicism in PBMCs, we also implemented outlier filters based on BAF in plasma (0.1 < Plasma BAF < 0.9) and normal tissue (0.2 < Normal BAF < 0.8) at the individual SNP level. To enrich for local signal, we aggregated SNPs into bins of 50 SNPs matched with other SNPs from the same tumor copy number state (e.g., major copy number 2 and minor copy number 0 as assigned by Sequenza in cnLOH in a ploidy 2 tumor sample). Outlier bins (BAF > 2 standard deviations from the sample mean for each copy number state) were excluded.

We reasoned that quantifying allelic imbalance from ctDNA in plasma cfDNA is similar to using BAF to estimate tumor fraction in impure tumor samples^78^. Thus, bin-level allelic imbalance score can be calculated from plasma TF and total and major copy numbers for tumor and normal per the following published equation^78^:

Eq. 5 $$\;BAF=\frac{TF*\;MajorCN_{t}+\;MajorCN_{n}(1-TF)}{TF*(\;TotalCN_{t})+\;TotalCN_{n}(1-TF)}$$

Where *TF* denotes the tumor fraction, *MajorCN*_*t*_ and *MajorCN*_*n*_ denote the copy number of the greater expressed allele in tumor and normal, respectively, and *TotalCN*_*t*_ and *TumorCN*_*n*_ denote total ploidy of tumor and normal, respectively.

In a diploid normal where TF is very small, as is the case in cfDNA from early-stage cancer and MRD, major allele fraction attributable to normal as described in the second term of Eq. 6 should approximate 0.5.

Eq. 6 $$\frac{MajorCN_{n}(1-TF)}{TF*\left(TotalCN_{t}\right)+TotalCN_{n}(1-TF)}\approx \frac{1}{2}$$

We can represent observed BAF in terms of coverage as follows:

Eq. 7 $$BAF_{obs}=\frac{cov_{m}}{cov_{t}}$$

Where *cov*_*t*_ denotes total read counts and *cov*_*m*_ denotes number of reads in a given site or bin expressing the major allele.

This, when combined with assumptions made in Eq. 7, allows us to reframe Eq. 6 to characterize the relationship between TF, copy number, and number of reads in a SNP or bin:

Eq. 8 $$\frac{cov_{m}}{cov_{t}}=\frac{\;TFMajorCN\;}{TF(\;TotalCN\;)+2(1-TF)}+\frac{1}{2}$$

Isolating for cellularity produces the following equation:

Eq. 9 $$\;BAFScore\;=\frac{-4cov_{M}+2cov_{T}}{2cov_{m}\;TotalCN\;-4cov_{M}-cov_{T}\;TotalCN\;+2cov_{T}-2cov_{T}\;MajorCN\;}$$

We then fit our observed BAFScore values and copy number calls, aggregated into equal sized bins of SNPs (n=50), to this equation using linear least squares regression (sklearn) to yield a sample-wide BAF score. Sample-level BAF scores in cancer plasma were compared to controls in matching genomic regions to produce a final sample-level Z score that reflected the contribution of ctDNA in cancer plasma compared to noise in non-cancerous control plasma.

### Evaluation of tumor-informed fragment length entropy

We calculated fragment length entropy to capture the heterogeneity of fragment insert size for cfDNA fragments within consecutive non-overlapping 100-kb genomic windows. We restricted analyses to fragments with insert size between 100–240bp. First, we calculated in each window the fraction of fragment sizes in each 5bp interval from 100 – 240bp. We then calculated Shannon’s entropy on the set of these fractional inputs. At the sample level, we converted window entropy values from all 100-kb windows (neutral and CNV) to median-normalized robust Z scores. By normalizing to the distribution of entropy values in each sample, neutral regions served as an internal control that accounted for the baseline fragment length heterogeneity within each sample inclusive of entropy noise from different sample preparations and pre-analytic biases. Following normalization, we multiplied window-level Z scores based on the direction of the CNV change using our underlying knowledge of tumor events. We expected more fragment length entropy from the contribution of additional ctDNA fragments in tumor amplifications and thus multiplied these values by +1, versus less fragment length entropy from the contribution of fewer ctDNA fragments in tumor deletions and therefore multiplied these values by -1. We noted that recurrent amplifications in chromosome 1p and 22q were uniformly present in control plasma samples in HiSeq controls (*n*=38 plasma samples) and NYGC controls (*n*=35 plasma samples), and these regions were excluded from analysis as likely cfDNA WGS-specific artifacts.

At the sample level, we aggregated signed window-level CNV Z scores (after multiplication by expected direction based on matched tumor amplification / deletions) across windows to generate a sample-level fragment length entropy score. Sample-level fragment length entropy scores in cancer plasma were compared to controls in matching genomic regions to produce a final sample-level Z score that reflected the contribution of ctDNA in cancer plasma compared to noise in non-cancerous control plasma.

### Aggregation of CNV scores

Our 3 CNV features (read depth, fragment length entropy, and BAF) independently informed our estimation of ctDNA signal. We therefore aggregated our features into an MRD-EDGE^CNV^ Z score by combining Z scores using Stouffer’s method $Z=\frac{\Sigma_{i=1}^{k}Z_{i}}{\sqrt{k}}$.

### Integration of MRD-EDGE^SNV^ and MRD-EDGE^CNV^ scores to form MRD-EDGE

To combine MRD-EDGE^SNV^ and MRD-EDGE^CNV^ into a composite MRD-EDGE Z score, we combined the Z scores for both platforms independently using Stouffer’s method. This was performed in the preoperative setting to assess the combined sensitivity of the MRD-EDGE^SNV^ and MRD-EDGE^CNV^ classifiers.

In the assessment of MRD and ctDNA shedding from adenomas and pT1 lesions, MRD-EDGE^SNV^ and MRD-EDGE^CNV^ classifiers provided orthogonal sources of information and were used to independently quantify ctDNA. We evaluated MRD and pT1 / adenoma detection as a sample-level Z score above either the MRD-EDGE^SNV^ or MRD-EDGE^CNV^ Z score threshold, obtained through calculating the 95% specificity boundary compared to plasma from healthy controls matched against the same patient-specific mutational compendium. For example, in stage III CRC, we defined a positive detection as a Z score threshold above 95% specificity against healthy control plasma sequenced at the same laboratory (Aarhus University) and sequencer (Illumina NovaSeq with v1.5 flow cells). We applied these same, prespecified Z score thresholds to identify postoperative MRD (Fig. 3b) in our pT1 and adenoma lesions (Fig. 4a), and our HiSeq CRC samples (Supplementary Fig. 4). The same was done in NSCLC for our neoadjuvant immunotherapy cohort (Fig. 3g), and the same, prespecified Z score threshold was applied to identify MRD in our HiSeq NSCLC samples (Supplementary Fig. 7). For MRD-EDGE^CNV^ application in TNBC cohort, we defined MRD-detection as an MRD-EDGE^CNV^ Z score in excess of 95% specificity among held-out control samples (Fig. 3m).

### Evaluating SNVs for *de novo* mutation calling in MRD-EDGE^SNV^

We collected all variants against the hg38 reference genome through samtools (v.3.1) mpileup with no exclusion filters. Only SNVs mapping to chromosomes 1 - 22 were included in our analysis. Indels were excluded. We ran a custom Python (v3.6.8) script to collect all fragments containing SNVs that matched pileup variants from the bam alignment. Fragments were then subjected to quality filters and the recurrent artifact blacklist and encoded as inputs to our model architecture (see **SNV deep learning model architecture and model training)**. We defined SNV detection rate, a function of the two unknown variables plasma TF and tumor mutational burden (TMB), as the number of fragments classified as ctDNA over the number of post-filter fragments evaluated.

### Determination of *de novo* mutation calling specificity threshold

In a tumor agnostic setting (*de novo* mutation calling), our datasets were more heavily imbalanced between signal and noise than in the tumor-informed setting, where knowledge of tumor SNVs was used to inform candidate variants. We determined the specificity threshold for *de novo* mutation calling within our plasma-only MRD-EDGE^SNV^ deep learning classifier by optimizing the trade-off at the fragment level between increasing signal enrichment at higher specificity thresholds (Supplementary Fig. 9a) vs. decreasing signal availability from overly stringent filtering (Supplementary Fig. 9b). We therefore evaluated performance of our classifier at high specificity thresholds within *in silico* TF admixtures of MEL-100 and a healthy control plasma sample (CTRL-216, Supplementary Table 4). We evaluated detection sensitivity vs TF=0 in admixtures TF=5*10^-5^ and found AUC to be highest at a specificity threshold of 0.995 (Supplementary Fig. 9b), with decreasing AUC at 0.9975 and 0.9925. We used this empirically chosen specificity threshold for evaluation of plasma TF in subsequent *de novo* mutation calling analyses. The MEL-100 plasma sample used in threshold determination was excluded from all downstream analysis.

### Evaluating TF trends with MRD-EDGE^SNV^, tumor-informed panel, and de novo panel

While in the tumor-informed setting, the fraction of mutations from the tumor detected in the plasma can serve to estimate TF (Methods), in the *de novo* setting, the number of mutations in the tumor is unknown. To enable dynamic assessment of plasma ctDNA burden, we used a normalized detection rate (nDR) to evaluate MRD-EDGE^SNV^ TF trends. Detection rates (fragments detected / fragments evaluated) in subsequent timepoints were normalized to the pretreatment TF to indicate increasing or decreasing plasma TF. For comparison in targeted panels, VAF across all mutations was normalized to the pretreatment timepoint (‘normalized VAF’, nVAF).

### Application of MRDetect support vector machine (MRDetect^SNV^) and MRDetect^CNV^

MRDetect^SNV^ and MRDetect^CNV^ were applied as described previously^14^. Both components were trained on NovaSeq samples for clinical application in NovaSeq and trained on Illumina HiSeq samples for clinical application in HiSeq. Application of both classifiers in the HiSeq setting was performed as described previously^14^. For application in NovaSeq, plasma samples included in the MRDetect^CNV^ plasma reference sample are listed in Supplementary Table 15.

### ichorCNA

ichorCNA^53^ (version 2.0) was used as an orthogonal CNA-based method for cfDNA detection and the estimation of plasma TF in high-burden plasma samples. We optimized the input setting for more sensitive detection in low-tumor-burden disease using the modified flags - altFracThreshold 0.001, -normal .99 along with a GRCh38 panel of normal (https://gatk.broadinstitute.org/). All other settings were set to default values.

### Evaluation of TF through ddPCR

A set of plasma samples (*n* = 48, Supplementary Table 15) from CRC patients were analyzed by ddPCR using the Bio-Rad platform. For each patient, a single clonal mutation was chosen for ddPCR analysis based on whole-exome sequencing of the patient’s tumor as previously described^52^. The ddPCR approach, including assay design, cycling optimization, and error correction was performed as previously described^79,80^. In brief, all ddPCR assays consisted of a single primer set amplifying the target regions, one mutation-specific probe, and a wild-type-specific probe. An assay-specific noise profile was generated for each assay by analyzing PBMC DNA from healthy donors. For each patient sample, cfDNA from 8 mL of plasma was used for ddPCR analysis. Each ddPCR setup included a no-template control (water), a tumor DNA positive control, and a PBMC DNA negative control. Droplets were generated by the Automated Droplet Generator (Bio-Rad) and read on the QX200™ Droplet Reader (Bio-Rad). The CASTLE (v.1.0) algorithm^79^ was used to compare the observed plasma signal to the assay-specific noise profile thereby statistically determining the ctDNA status and variant allele frequency (VAF) of each sample.

### Tumor-informed and *de novo* targeted panel

MSK-ACCESS^7^ was used as an orthogonal SNV-based method for evaluation of plasma TF in melanoma samples. MSK-ACCESS was run independently on a subset of pre- and posttreatment plasma samples for 14 patients with cutaneous melanoma with available material allowing concurrent analysis. Application of MSK-ACCESS panel and data analysis was performed by the MSK-ACCESS team. Results for the tumor-informed panel were informed by somatic mutations found in matched tumor samples through MSK-IMPACT^81^ and were reported as average adjusted VAF across evaluated genes. Criteria for detection was 1 supporting duplex read or 2 simplex reads found in plasma in the tumor-informed setting in accordance with the MSK-ACCESS mutation calling pipeline.

VAF was adjusted to account for copy number alterations at the locus of interest. Copy number alterations are inferred by applying FACETS^82^ to Whole Exome or Whole Genome tumor tissue used in MSK-IMPACT analysis. The ACCESS team assumes that there are no changes to copy numbers of these segments between the IMPACT and ACCESS samples. Adjusted VAF is calculated as follows

Eq. 10 $$VAF=\frac{T_{ALT}*TF}{T_{CN}*TF+N_{CN}*(1-TF)}$$

Where *VAF* is the expected variant allele fraction, *TF* is tumor fraction, *T*_*ALT*_ = alternate copies in tumor, *T*_*CN*_ = total copies in tumor, and *N*_*CN*_ = total copies in normal.

Solving the equation for *TF* yields:

Eq. 11 $$TF=\frac{N_{CN}*VAF}{T_{ALT}+\left(N_{CN}-T_{CN}\right)*VAF}$$

For ACCESS samples, this TF value is computed and named adjusted *VAF* (*VAF*_*adj*_). For the *de novo* panel, only adjusted VAFs above 0.005 contributed to average VAF. Reads classified as containing a somatic mutation and total reads evaluated for use in MSK-ACCESS *VAF*_*adj*_ are listed in Supplementary Table 9.

### Statistical analysis

Statistical analyses were performed with Python 3.6.8 and R version 3.6.1. Continuous variables were compared using Student’s *t*-test, the Wilcoxon rank-sum test or the nonparametric permutation test, as appropriate. All *P* values were two sided and considered significant at the 0.05 level, unless otherwise noted. Cox proportional hazards models were fit using lifelines^83^ and forest plots were plotted using EffectMeasurePlot from zEpid^84^ (v0.9.0).

### Data exclusions

Perioperative plasma samples (*n*=2) from the HiSeq NSCLC patient NSCLC-136 was excluded from analysis because of low tumor purity which prevented identification of SNVs or tumor aneuploidy.

## Extended Data

## Supplementary Material

Extended Data Fig. 1

Supplementary Fig. 1

Supplementary Tables

## Data Availability

Clinical samples sequenced at the New York Genome Center are available from the European Genome-Phenome Archive (EGA) under the accession codes EGAS00001007306 and EGAS00001007545. Sequence data from previous work^14^ is available from the EGA under accession codes EGAS00001004406 and EGAS00001007451. For clinical samples from Aarhus University, to protect the privacy and confidentiality of patients in this study, personal data including clinical and sequence data are not made publicly available in a repository or the supplementary material of the article. The data can be requested at any time from Claus Lindbjerg Andersen’s laboratory (cla@clin.au.dk). Any requests will be reviewed within a time frame of 2 to 3 weeks by the data assessment committee to verify whether the request is subject to any intellectual property or confidentiality obligations. All data shared will be de-identified. Clinical information and sequencing metrics pertinent to clinical samples from Aarhus University are therefore withheld from Supplementary Tables and present in Restricted Tables as indicated in Supplementary Tables 1-16. Access to clinical data and processed sequencing data output files (Mutect2 v4.2.4.1, Strelka2 v2.9.10, and FACETS v0.6.2) used in the article requires that the data requestor (legal entity) enter into Collaboration and Data Processing Agreements, with the Central Denmark Region (the legal entity controlling and responsible for the data). Request for access to raw sequencing data furthermore requires that the purpose of the data re-analysis is approved by The Danish National Committee on Health Research Ethics. Upon a reasonable request, the authors, on behalf of the Central Denmark Region, will enter into a collaboration with the data requestor to apply for approval. Additional info can be found at https://genome.au.dk/library/GDK000010/.

## Code availability

Computer code and computational models for reproducing results from this study is included in revision materials and will be available upon request from the Center for Technology Licensing at Cornell University for academic (non-commercial) groups. The code can be accessed at the following address:

Link: ctl.cornell.edu/industry/mrdedge-license-request/
