# Supplementary material for: Ultrasensitive plasma-based monitoring of tumor burden using machine learning-guided signal enrichment
Source: Nat Med. Author manuscript; Available in PMC 2024 Jul 10. (PMC7616143; doi:10.1038/s41591-024-03040-4)
Supplement: Extended Data Fig. 1 [file EMS197223-supplement-Extended_Data_Fig__1.docx]

**Extended Data Figures**

**Extended Data Fig. 1, Widman et al.**

**
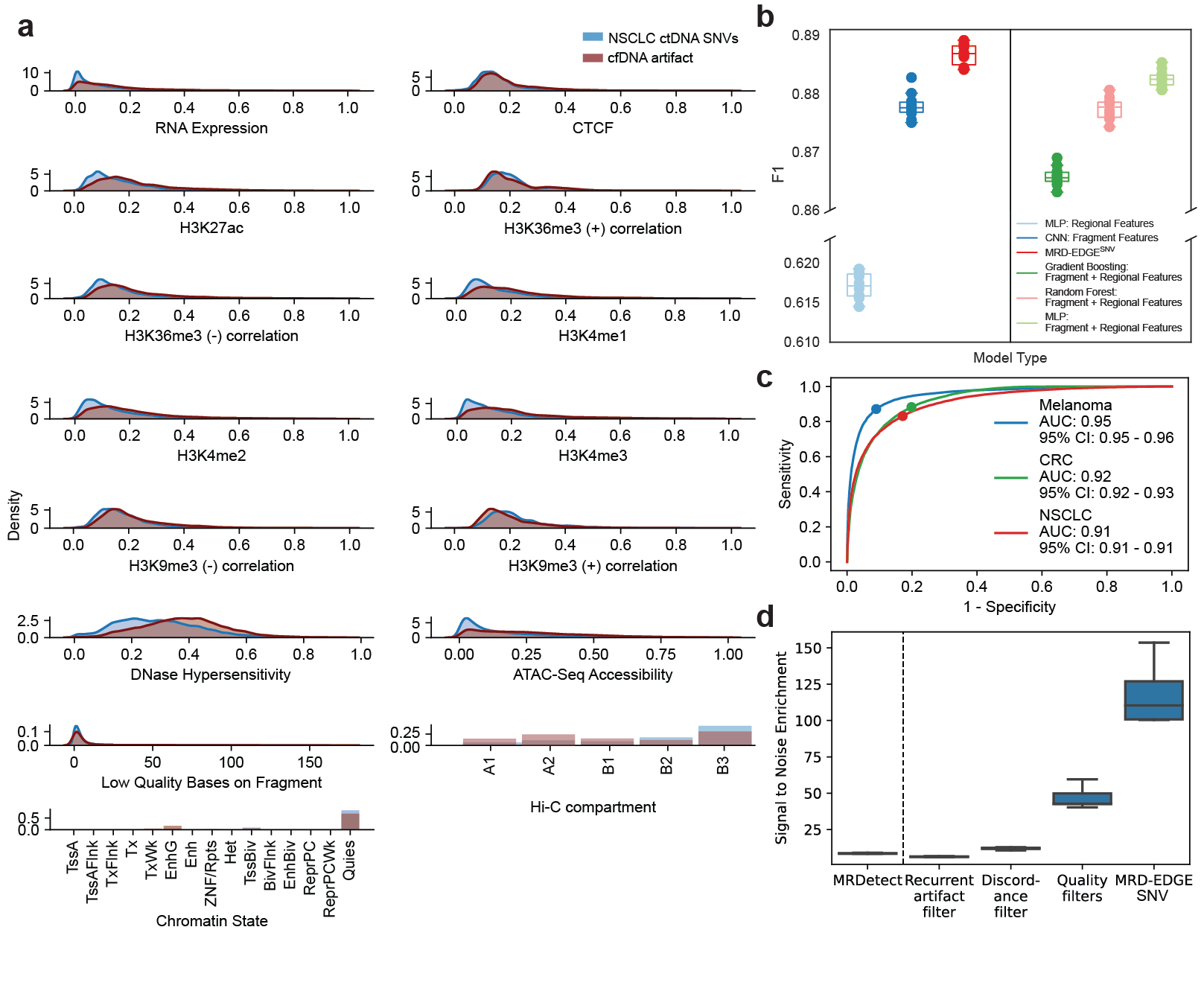
**

**Extended Data Fig. 1: MRD-EDGE^SNV^ feature selection, model architecture and performance**

**a)** Feature density plots for ctDNA and cfDNA SNV artifacts used in the MRD-EDGE^SNV^ NSCLC model. These fragments were subject to quality filters (Supplementary Table 2) to remove low quality SNV artifacts prior to this analysis. In this comparison, ctDNA SNV fragments are identified from consensus mutation calls in high-burden NSCLC plasma samples (Supplementary Table 1) and compared to cfDNA SNV fragments (sequencing errors) drawn from within the same plasma sample to preclude sample-specific biases when establishing predictive ability of individual features. **b)** SNV classification performance for different machine learning models. F1 score was assessed on tumor-confirmed melanoma ctDNA SNV fragments vs. cfDNA artifacts from healthy controls. Random subsamplings were drawn from the held-out melanoma validation set (Supplementary Table 1), which was split into tenths for this analysis. We compared performance between MRD-EDGE^SNV^ and its separate components (left), as well as to other ML architectures (right) **c)** Fragment-level ROC analysis for MRD-EDGE^SNV^ classifier for different cancer types. Performance is assessed on filtered fragments (~90% of low-quality cfDNA artifacts are excluded by quality filters) in held-out validation sets (Supplementary Table 1) for melanoma (blue), CRC (green), and NSCLC (red). Colored dots on curves indicate the tumor-informed decision threshold (0.5) used in each tumor type to classify individual SNV fragments as ctDNA or cfDNA artifact. **d)** Signal-to-noise enrichment analysis for MRDetect and for each step of the MRD-EDGE^SNV^ tumor-informed pipeline. Final pipeline enrichment is 118-fold for MRD-EDGE^SNV^ vs. 8.3-fold for MRDetect^SNV^ in the same datasets.

**Extended Data Fig. 2, Widman et al.**

**
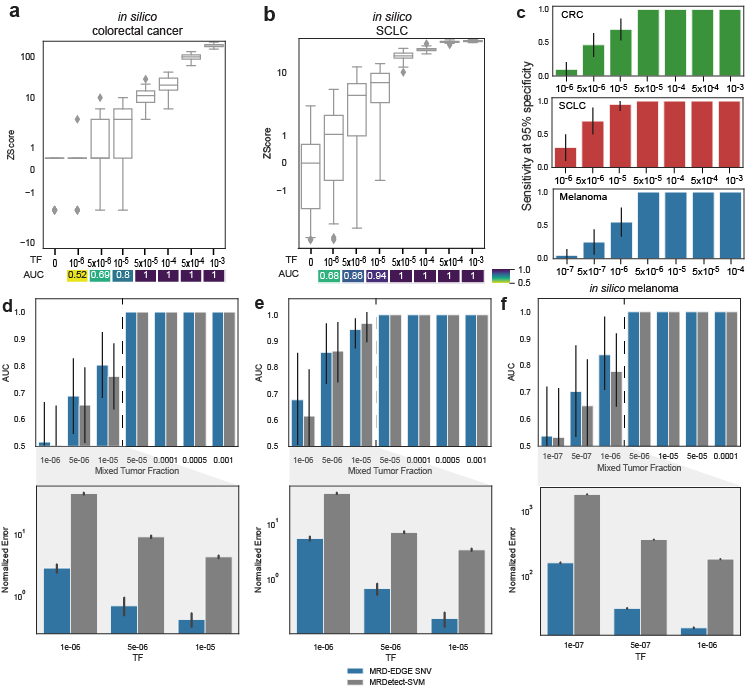
**

**Extended Data Fig. 2: Lower limit of detection studies with MRD-EDGE^SNV^**

**a)** *In silico* studies of cfDNA from the metastatic colorectal cancer sample CRC-863 mixed into cfDNA from a healthy plasma sample (CTRL-335) at mixing fractions TF = 10^-6^–10^-3^ at 29X coverage depth, performed in 30 technical replicates with independent sampling seeds. Tumor-informed MRD-EDGE^SNV^ enables sensitive detection of TF as low as 1*10^-5^ (AUC 0.80), measured by Z score of SNV detection rates against unmixed control plasma (TF=0, n=30 randomly chosen replicates). **b)** *In silico* studies of cfDNA from the metastatic small cell lung cancer sample SC-128_0w mixed into cfDNA from a healthy plasma sample (CTRL-216) at mixing fractions TF = 10^-6^–10^-3^ at 25X coverage depth, performed in 20 technical replicates with independent sampling seeds. Tumor-informed MRD-EDGE^SNV^ enables sensitive detection of TF as low as 5*10^-6^ (AUC 0.86), measured by Z score of SNV detection rates against unmixed control plasma (TF=0, *n*=20 randomly chosen replicates). Box plots represent median, lower and upper quartiles; whiskers correspond to 1.5 x interquartile range. An AUC heatmap measures detection vs. TF=0 at different mixed TFs*.* **c)** Sensitivity at 95% specificity for tumor-informed MRD-EDGE^SNV^ *in silico* studies in green) CRC, red) SCLC, and blue) melanoma. Mixed TF replicates were compared to TF=0 replicates by sample-level MRD-EDGE^SNV^ Z score. **d-f)** Detection performance vs. TF=0 at different mixed TFs for MRD-EDGE^SNV^ (blue) and MRDetect^SNV^ SVM (gray). The AUC is measured by a sample Z score (positive label) compared to TF=0 distribution (negative label) for each replicate at each TF. Error bars represent 95% CI (DeLong AUC variance). (bottom) Normalized error for a subset of mixed TFs between MRD-EDGE^SNV^ and MRDetect^SNV^. Error bars represent 95% CI. Normalized error is shown for TFs where AUC is less than 1 and is measured as (TF_estimated_-TF_mixed_)/TF_mixed_. **d)** *in* *silico* CRC studies as defined in **(a), e)** in silico SCLC studies as defined in **(b), f)** *In silico* studies of cfDNA from the metastatic cutaneous melanoma sample MEL-100 mixed into cfDNA from a healthy plasma sample (CTRL-216) at mixing fractions TF = 10^-7^–10^-4^ at 16X coverage depth, performed in 20 technical replicates with independent sampling seeds. .

**Extended Data Fig. 3, Widman et al.**

**
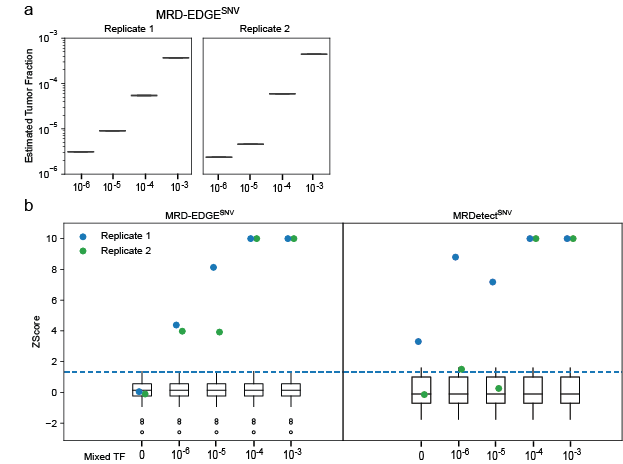
**

**Extended Data Fig. 3: Estimated tumor fractions in experimental mixing studies with MRD-EDGE^SNV^**

**a)** Plasma TF inference with MRD-EDGE^SNV^ using genome-wide SNV integration for *in vitro* dilutions of the pretreatment melanoma plasma MEL-137_A in expired plasma harvested through plasmapheresis from a donor without known cancer. Dilutions were performed in 2 replicates, and a mean noise rate for the patient-specific mutation profile was drawn from *n*=17 concurrently sequenced SCLC plasma samples (Supplementary Table 5). **b)** MRD-EDGE^SNV^ (left) and MRDetect^SNV^ (right) Z score discrimination between ctDNA detected in experimental plasma replicates (blue dots, replicate 1, and green dots, replicate 2) from the patient MEL-137 and downsampled TF=0 replicates (white boxes, n=30, 15 downsampled alignment files from 2 TF=0 replicates). Signal is measured from SNV detection rates on patient plasma and the downsampled TF=0 plasma samples using the patient-specific SNV profile for MEL-137. Positive ctDNA detection (dotted blue line) was defined as patient plasma MRD-EDGE^SNV^ or MRDetect^SNV^ Z score above a detection threshold of 95% specificity against downsampled TF=0 plasma in the ROC for each platform (Supplementary Table 4). Sample-level Z scores were capped at 10 to allow greater visibility of Z scores around the detection threshold.

**Extended Data Fig. 4, Widman et al.**

**
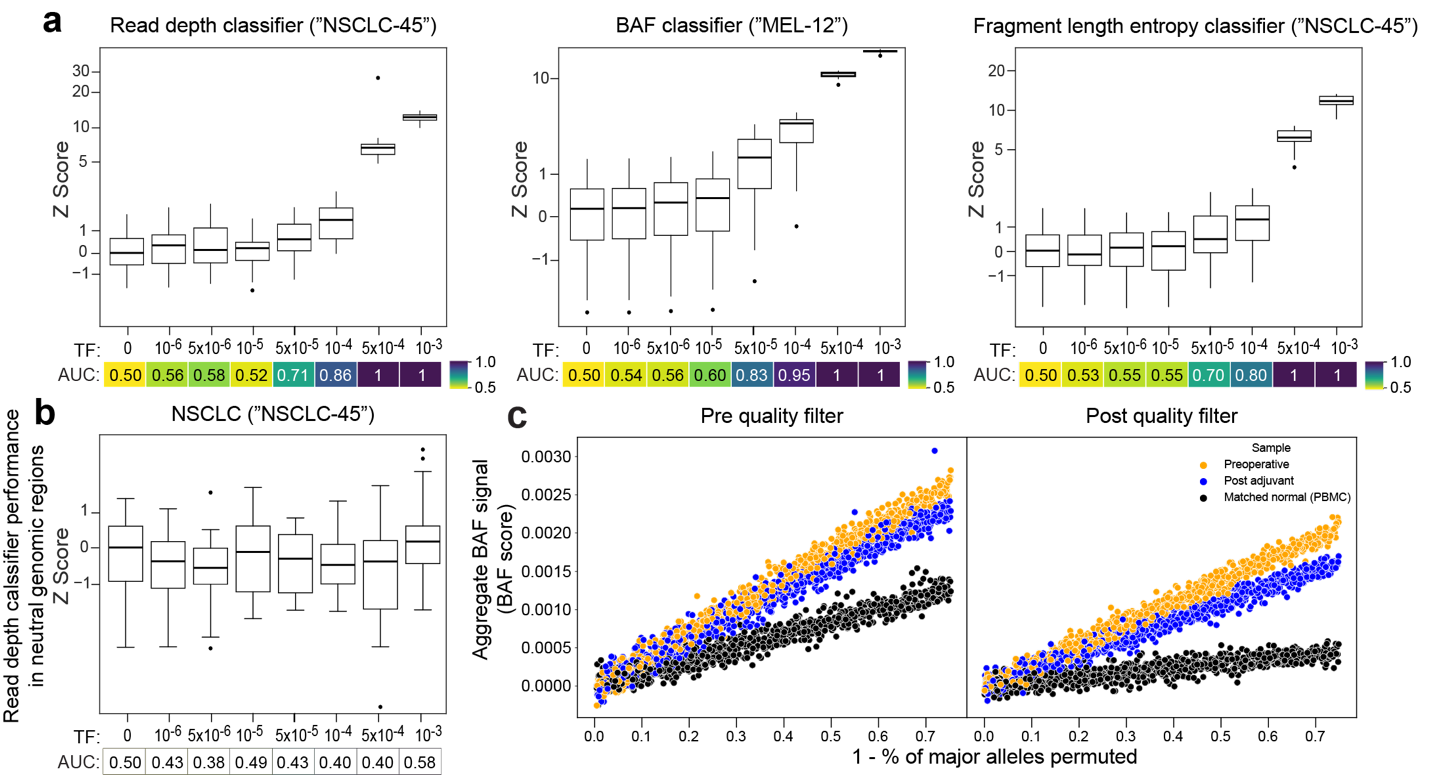
**

**Extended Data Fig. 4: *In silico* mixing studies of MRD-EDGE^CNV^ in CRC, NSCLC, and melanoma**

**a,b**) *In silico* mixing studies in which high TF plasma samples were admixed into non-cancer plasma (**a**) or low TF plasma samples (**b**). Admixtures model tumor fractions of 10^-6^–10^-3^ (see Methods for detailed description of *in silico* admixture process). Box plots represent median, lower and upper quartiles; whiskers correspond to 1.5 x interquartile range. An AUC heatmap demonstrates detection performance vs. TF=0 at different mixed TFs as measured by a sample Z score (derived from summed read-depth skews for read depth classifier, BAF score for BAF classifier, summed fragment length entropy for fragment length entropy classifier, Methods) compared to TF=0 distribution for each replicate. **a**) Pretreatment NSCLC plasma from the patient NSCLC-45 was mixed into non-cancer control plasma from the patient CTRL-206 in 25 technical replicates (each subsampling seed represents a technical replicate). The read depth (left) and fragment length entropy (right) classifiers demonstrate similar performance in pretreatment NSCLC admixtures compared to CRC admixtures (**Fig. 2b-d**). (middle) Pretreatment melanoma plasma from the patient MEL-12 was mixed into posttreatment plasma following a major response to immunotherapy in 25 technical replicates. The BAF classifier demonstrates similar performance compared to CRC admixtures (**Fig. 2c**) and accounts for bias that may be encountered when mixing plasma into matched peripheral blood mononuclear cell (PBMC) normal, as performed in CRC. **b**) Z scores for the read depth classifier in neutral regions (no copy number gain or loss in the matched tumor WGS data) for NSCLC demonstrates the expected absence of directional read depth skew in copy neutral regions. **c**) Assessment of preoperative plasma, post adjuvant plasma, and matched normal (from PBMCs) BAF in SNPs before (left) and after (right) SNP quality filters in CRC (patient CRC-465). Filters include mapping bias correction and outlier exclusion criteria (Methods). BAF signal is calculated through least squares linear regression on SNPs from LOH regions identified in matched tumor WGS, accounting for underlying copy number state in tumors (Methods). To demonstrate the relationship between signal and phased SNPs, the major allele in plasma is randomly permuted to be in phase or out of phase at the percentage specified along the x axis. Following quality filtering, signal can be appropriately inferred and demonstrates the expected relationship between preoperative plasma (highest signal), postoperative MRD (intermediate signal), and PBMC BAF (minimal signal).

**Extended Data Fig. 5, Widman et al.**

**
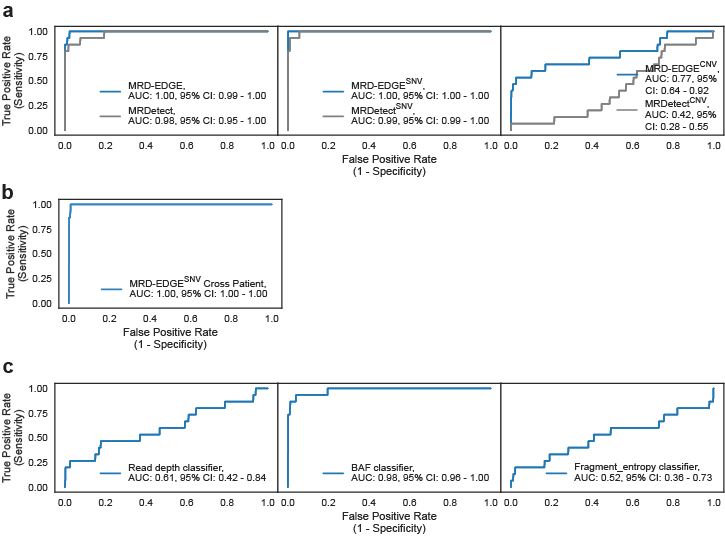
**

**Extended Data Fig. 5: Clinical performance of tumor-informed MRD-EDGE in stage III perioperative colorectal cancer**

**a)** (left) ROC analysis on MRD-EDGE (blue), a combined detection model of SNV and CNV mutation profiles, and MRDetect (gray) in preoperative stage III CRC. Preoperative plasma samples with matched tumor mutation profiles (*n*=15, Supplementary Table 5) are compared with control plasma samples assessed against all unmatched stage III CRC tumor mutation profiles (*n*=15 tumor profiles assessed across 25 control samples from Aarhus controls cohort, *n*=375 control-comparisons). Twenty control samples included in SNV model training and / or used in the MRD-EDGE^CNV^ read depth panel of normals were withheld from this analysis*.* (middle) ROC analysis with MRD-EDGE^SNV^ (blue), and MRDetect^SNV^ (gray). Preoperative plasma samples with matched tumor mutation profiles (*n*=15) are compared with unmatched control plasma samples assessed against all unmatched stage III CRC tumor mutation profiles (*n*=15 tumor profiles assessed across 40 control samples from Aarhus controls cohort, *n*=600 control-comparisons). Five control samples included in SNV model training were withheld from this analysis*.* (right) ROC analysis with MRD-EDGE^CNV^ (blue), and MRDetect^CNV^ (gray). Preoperative plasma sample CNV-based Z scores (*n*=15) are compared against control plasma samples assessed against all unmatched stage III CRC tumor mutation profiles (*n*=15 tumor profiles assessed across 25 control samples from Aarhus controls cohort, *n*=375 control-comparisons). Twenty control samples included in the read depth panel of normals were withheld from this analysis*.* **b)** Cross-patient ROC analysis on preoperative stage III CRC plasma samples for MRD-EDGE^SNV^ demonstrates similar performance to control (non-cancer) plasma. Preoperative plasma samples with matched tumor profiles (*n*=15) are compared with stage III CRC plasma samples assessed against all unmatched stage III CRC tumor profiles (*n*=15 tumor profiles assessed across 14 cross-patient samples, *n*=210 cross-comparisons). **c)** ROC analysis performed on CNV-based Z-score values for read depth (left), BAF (middle), and fragment length entropy (right) CNV classifiers in preoperative stage III CRC. Preoperative plasma samples with matched tumor profiles (*n*=15) are compared with control plasma samples assessed against all unmatched tumor profiles (*n*=375 comparisons for read depth, 15 tumor profiles assessed across 25 control samples; *n*=675 comparisons for BAF and fragment length entropy, 15 tumor profiles assessed across 45 control samples). Twenty control samples included in the read depth panel of normal samples were withheld from read-depth analysis.

**Extended Data Fig. 6, Widman et al.**

**
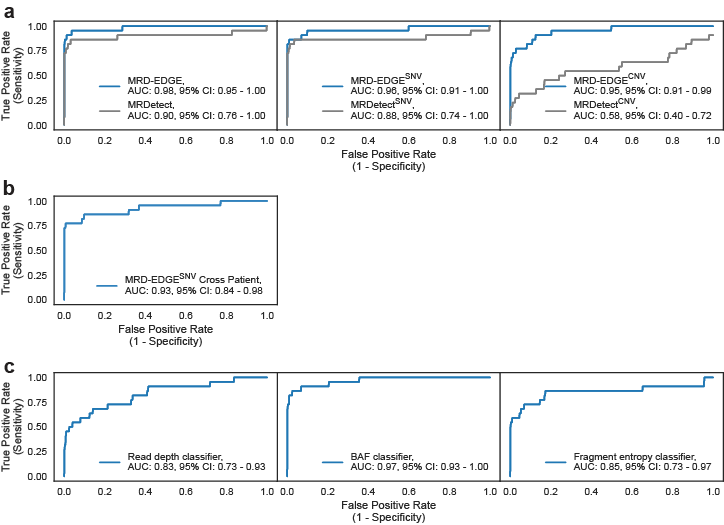
**

**Extended Data Fig. 6: Comparison of MRD-EDGE and MRDetect in preoperative, pretreatment NSCLC**

**a)** (left) ROC analysis of NSCLC plasma samples for MRD-EDGE (blue) and MRDetect (gray). NSCLC plasma samples with matched tumor profiles (*n*=22 samples, Supplementary Table 5) are compared with control plasma samples assessed against all unmatched NSCLC tumor mutation profiles (*n*=22 tumor profiles assessed across 20 control samples from NYGC controls cohort, *n*=440 control-comparisons). (middle) ROC analysis of NSCLC plasma samples for MRD-EDGE^SNV^ (blue) and MRDetect^SNV^ (gray). NSCLC plasma samples with matched tumor profiles (*n*=22, Supplementary Table 5) are compared with control plasma samples assessed against all unmatched NSCLC tumor mutation profiles (*n*=22 tumor profiles assessed across 40 control samples from NYGC controls cohort, *n*=660 control-comparisons). Five patients used in MRD-EDGE^SNV^ NSCLC model training were excluded from downstream analysis. (right) ROC analysis of NSCLC plasma samples for MRD-EDGE^CNV^ (blue) and MRDetect^CNV^ (gray). NSCLC plasma samples with matched tumor profiles (*n*=22, Supplementary Table 5) are compared against control plasma samples assessed against all unmatched NSCLC tumor mutation profiles (*n*=22 tumor profiles assessed across 20 control samples from NYGC controls cohort, *n*=440 control-comparisons). Fifteen patients used in the read depth panel of normal samples were excluded from downstream analysis. **b)** Cross-patient ROC analysis on pretreatment NSCLC tumor profiles for MRD-EDGE^SNV^ demonstrates similar performance to control (non-cancer) plasma. Preoperative plasma samples with matched tumor profiles (*n*=22) are compared with NSCLC plasma samples assessed against all unmatched NSCLC tumor profiles (*n*=22 tumor profiles assessed across 21 cross-patient samples, *n*=462 cross-comparisons). **c)** ROC analysis performed on CNV-based Z-score values for read depth (left), BAF (middle), and fragment length entropy (right) CNV classifiers in preoperative stage III CRC. Preoperative plasma samples with matched tumor profiles (*n*=22) are compared with control plasma samples assessed against all unmatched tumor profiles (*n*=440 comparisons for read depth, 22 tumor profiles assessed across 20 control samples; *n*=770 comparisons for BAF and fragment length entropy, 22 tumor profiles assessed across 35 control samples). Twenty control samples included in the read depth panel of normal samples were withheld from read-depth analysis.

**Extended Data Fig. 7, Widman et al.**

**
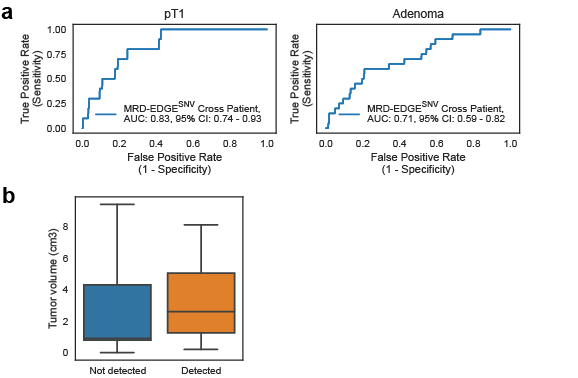
**

**Extended Data Fig. 7: MRD-EDGE detection of ctDNA from colorectal pT1 carcinomas and adenomas**

**a)** Cross-patient ROC analysis for MRD-EDGE^SNV^ in screen-detected pT1 lesions (left) and adenomas (right). Preoperative plasma samples with matched tumor mutation profiles are compared with a cross-patient panel of plasma samples assessed against all unmatched cross-patient tumor profiles (*n*=44, including 29 pT1 and adenoma cross patients and 15 stage III preoperative patients).  **b)** Tumor resection volume for adenoma samples in which ctDNA was detected (orange) and non-detected (blue). Box plots represent median, bottom and upper quartiles; whiskers correspond to 1.5 x interquartile range.

**Extended Data Fig. 8, Widman et al.**

**
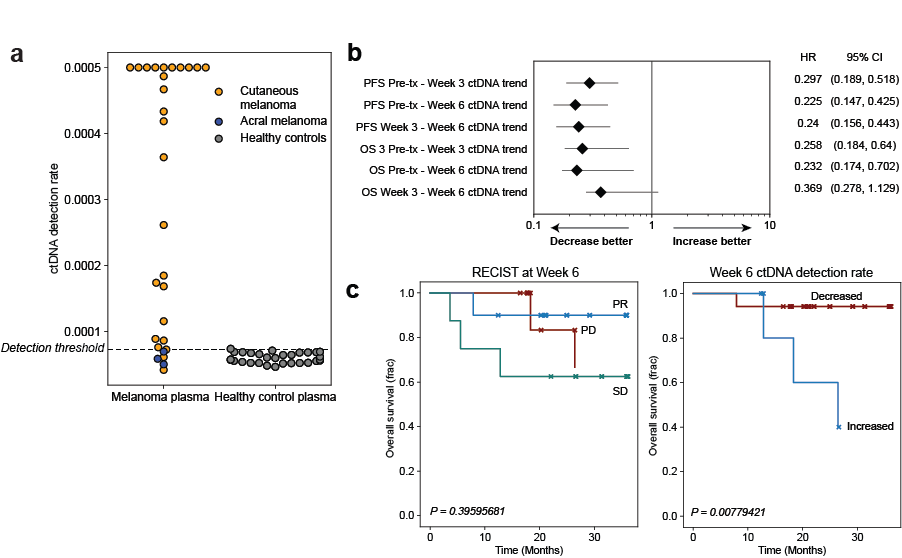
**

**Extended Data Fig. 8: Use of MRD-EDGE^SNV^ in acral melanoma and monitoring response to immunotherapy with MRD-EDGE^SNV^**

**a)** ctDNA detection rates for pretreatment cutaneous melanoma samples from the adaptive dosing cohort (*n*=26, orange, detection rate was capped at 0.0005) compared to acral melanoma samples (*n*=3, blue, pre- and posttreatment timepoints from one patient with acral melanoma) sequenced within the same batch and flow cell and detection rates as healthy control plasma (*n*=30, gray). ctDNA is not detected from acral melanoma plasma, demonstrating absence of batch effect and the specificity of MRD-EDGE^SNV^ for the UV signatures associated specifically with cutaneous melanoma. **b)** Forest plot demonstrating relationship between ctDNA TF trend (increase or decrease) and progression-free survival (PFS) and overall survival (OS) at serial posttreatment timepoints. MRD-EDGE^SNV^ TF estimates are measured as a detection rate normalized to the pretreatment sample (normalized detection rate, nDR). Each posttreatment timepoint is prognostic of PFS outcomes. HR, hazard ratio. **c)** (left) Kaplan–Meier overall survival analysis for Week 6 RECIST response (*n*=10 partial response, ‘PR’, *n*=8 stable disease, ’SD’, *n*=6 progressive disease, ‘PD’) in the adaptive dosing melanoma cohort (*n*=26 patients) where CT imaging was available at Week 6 shows no significant relationship with OS (multivariate logrank test). (right) Kaplan–Meier OS analysis for Week 6 ctDNA trend in adaptive dosing melanoma patients with decreased (*n*=17) or increased (*n*=5) nDR compared to pretreatment timepoint as measured by MRD-EDGE^SNV^. Patients with undetectable pretreatment ctDNA *(n=*2) were excluded from the analysis, as were 2 patients where Week 6 plasma was not available for analysis. Increased nDR at Week 6 was associated with shorter overall survival (two-sided log-rank test).

**Extended Data Fig. 9, Widman et al.**

**
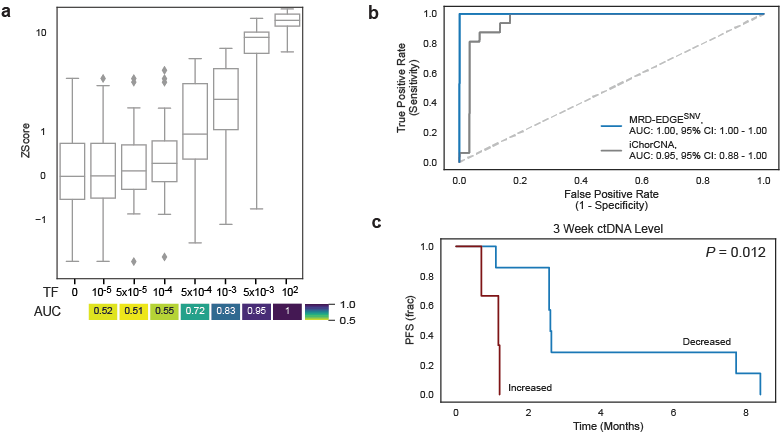
**

**Extended Data Fig. 9: Use of MRD-EDGE^SNV^ to monitor response to ICI in small cell lung cancer**

**a)** *In silico* studies of cfDNA from the SCLC sample SC-128 (pretreatment TF = 22.9%) mixed in *n*=20 replicates against cfDNA from a healthy plasma sample (TF=0) at mix fractions 10^-5^–10^-2^ at 25X coverage depth. MRD-EDGE^SNV^ enables sensitive detection of TF as low as TF=5*10^-4^ (AUC 0.72), measured by Z score of SNV fragment detection rate against unmixed control plasma (TF=0, n=20 randomly chosen replicates), without matched tumor tissue to guide SNV identification. Box plots represent median, bottom and upper quartiles; whiskers correspond to 1.5 x interquartile range. An AUC heatmap measures detection vs. TF=0 at different mixed TFs. **B)** ROC analysis on detection rates for MRD-EDGE^SNV^ (blue) and TF estimation with ichorCNA (gray) in pretreatment SCLC plasma samples (Supplementary Table 7). Fragment detection rates in SCLC plasma samples (*n*=16 plasma samples, Supplementary Table 5) were compared with fragment detection rates in control plasma samples (*n*=30). **C)** Kaplan–Meier progression-free survival analysis for Week 3 ctDNA trend in SCLC patients with decreased (*n*=7) or increased (*n*=3) normalized detection rate (nDR) as measured by MRD-EDGE^SNV^. Increased nDR at Week 3 was associated with shorter progression-free survival (two-sided log-rank test).
