# Supplementary material for: Ultrasensitive plasma-based monitoring of tumor burden using machine learning-guided signal enrichment
Source: Nat Med. Author manuscript; Available in PMC 2024 Jul 10. (PMC7616143; doi:10.1038/s41591-024-03040-4)
Supplement: Supplementary Fig. 1 [file EMS197223-supplement-Supplementary_Fig__1.pdf]

nature medicine

Supplementary information

https://doi.org/10.1038/s41591-024-03040-4

Ultrasensitive plasma-based monitoring of tumor burden using machine-learning-guided signal enrichment

In the format provided by the authors and unedited

Evaluation of neural network interpretability for MRD-EDGE^{SNV}. To assess behavior of individual MRD-EDGE^{SNV} features within a neural network, we converted all features to tabular values (see **Comparison of MRD-EDGE^{SNV} deep learning classifier performance to other machine learning models, Methods**) and trained MLPs for CRC, melanoma, and NSCLC according to the training sample paradigms in Supplementary Table 1. Aggregate feature importances (**Supplementary Fig. 1a**) and individual feature Shapley values (**Supplementary Fig. 1b**) were obtained from the application of GradientExplainer from the python SHAP[85] library (v0.37.0) to the trained model from each cancer type.

Discrimination of MRD-EDGE between in silico mixing TFs. We generated *in silico* TF admixtures (Methods) from the melanoma plasma sample MEL-100 mixed into cfDNA from an individual with no known cancer (**Fig. 1e,** Supplementary Table 4). In this *in silico* study, MRD-EDGE^{SNV} provided effective discrimination between mixing fractions, demonstrating accurate quantification of tumor burden (SNV AUCs in **Supplementary Fig. 3a**, *P* values from Student's t-test in **Supplementary Fig. 3b**). We further evaluated discrimination between mix fractions for the read depth, BAF, and fragment length entropy classifiers of MRD-EDGE^{CNV} (**Supplementary Fig. 4**).

Application of tumor-informed MRD-EDGE to HiSeq re-analysis cohorts. Though MRD-EDGE was trained on Illumina NovaSeq plasma samples, to demonstrate generalizability we also tested the platform on our previously reported[14] clinical cohort of Illumina HiSeq plasma samples from patients with CRC ("HiSeq CRC" *n*=19 patients, including 6 with microsatellite instability (MSI), Supplementary Table 6), compared with controls without known cancer (*n*=38) and from the same sequencing platform. As further proof of generalizability, we used the same detection thresholds as in our preoperative stage III CRC NovaSeq cohort. Composite MRD-EDGE and MRD-EDGE^{SNV} produced comparable performance to MRDetect in the preoperative setting (**Supplementary Fig. 5**). Moreover, the ability to evaluate cnLOH with MRD-EDGE^{CNV} allowed

us to apply CNV-based detection to 18 / 19 samples in this cohort, compared to 15 / 19 samples with MRDetect^{CNV} without any loss of performance. Postoperative plasma was drawn for each of these patients at a median of 43 days after surgery. In this postoperative setting, MRD-EDGE was highly specific for disease recurrence in microsatellite stable (MSS, *n*=13) samples (**Supplementary Fig. 5**) and was associated with shorter disease-free survival over a median follow up of 49 months (range 18-76). False positives in the postoperative setting were confined to a patient who received adjuvant chemotherapy and a patient with overall survival time below the median time to recurrence in CRC[86]. Among the broader cohort, association between postoperative ctDNA detection with MRD-EDGE and shorter disease-free survival did not reach statistical significance (*P*=0.0546, **Supplementary Fig. 5**) due to false positives among MSI samples with MRD-EDGE (6 of 6 MSI samples detected with MRD-EDGE^{SNV}). This suggests that due to distinct mutational signatures[18], patients with MSI tumors may require a separate SNV training paradigm or should only be evaluated with MRD-EDGE^{CNV}, which detected no false positives among MSI samples. Integrating the two CRC cohorts in a survival analysis, MRD-EDGE was highly sensitive and specific for disease recurrence in patients with MSS tumors ($P=7*10^{-4}$ logrank, **Supplementary Fig. 6**), demonstrating the outstanding potential for MRD detection with plasma WGS.

To demonstrate generalizability in another tumor type, we applied MRD-EDGE to a cohort of early-stage NSCLC patients evaluated previously[14] ("HiSeq NSCLC", Supplementary Table 5). Composite MRD-EDGE and MRD-EDGE^{SNV} performed similarly to MRDetect in the preoperative setting while MRD-EDGE^{CNV} had superior performance (**Supplementary Fig. 7**). MRD-EDGE performed comparably to MRDetect in the detection of postoperative MRD associated with shorter disease-free survival (logrank HR 6.4, $P=1.2*10^{-2}$ for MRD-EDGE vs HR 8.4, $P=3.7*10^{-3}$ for MRDetect, **Supplementary Fig. 8**).

Assessing statistical significance for adenoma detections. Detections for MRD-EDGE for pT1 lesions and adenomas were significantly above our expected false positive rate of 5% (binomial P=3*10^{-4} and 1.1*10^{-3}, respectively, accounting for detection opportunities with both MRD-EDGE^{SNV} and MRD-EDGE^{CNV,}). To more stringently demonstrate detection, we evaluated our detections against the lower limit of the 95% confidence intervals for specificity for MRD-EDGE^{SNV} (0.934) and MRD-EDGE^{CNV} (0.923) and found that detections surpassed the expected false positive rate in both cases (SNV: binomial P=1.2*10^{-3} for pT1 lesions and 4.6*10^{-3} for adenomas; CNV: binomial P=2.6*10^{-3} for pT1 lesions and 1.0*10^{-2} for adenomas).

For the detection of ctDNA shedding in adenomas and pT1 lesions, we further sought to provide orthogonal validation for our TF estimates using our *in silico* mixing analysis for CRC (**Extended Data Fig. 2a**), as the TFs of these lesions may be of interest to early detection efforts. For TF admixtures at 1*10^{-5}, comparable to the median adenoma estimated TF of 8.0*10^{-6}, 95% confidence interval was 7.3*10^{-6}-1.1*10^{-5} as calculated from a normal distribution and standard error of the mean of n =27 seeds with ≥ 1 fragment detected, similar to the TF range for detected adenomas range 5.7*10^{-6}-1.6*10^{-5}, from **Tumor-informed MRD-EDGE detects ctDNA shedding in precancerous adenomas and minimally invasive pT1 carcinomas**). This suggests that an assay sensitivity of 1*10^{-5} may be needed to detect precancerous lesions.

Specificity threshold for de novo mutation calling. To determine an appropriate *de novo* specificity threshold for our MRD-EDGE^{SNV} deep learning classifier (**Fig. 1d**) in melanoma we used the same *in silico* admixtures as in the tumor-informed setting (validation melanoma sample MEL-100 admixed with a held-out healthy control plasma sample, **Fig. 1e**). We compared signal-to-noise enrichment with detection AUC at different specificity thresholds imposed on the MRD-EDGE^{SNV} ensemble model output to find an optimal threshold for *de novo* classification of ultrasensitive TFs (TF 5*10^{-5}). As expected, our empirically chosen threshold in the *de novo*

classification context (0.995) was higher than the balanced threshold (0.5) used in the tumor-informed setting (**Extended Data Fig. 9a-b**, Methods).

MRD-EDGE additional fragment classification and generalizability analyses

Evaluation of fragment-level classification on sample level results. To evaluate the contribution of MRD-EDGE^{SNV} fragment-level classification to sample level results, we compared our tumor-informed WGS pipeline with and without application of the MRD-EDGE^{SNV} individual fragment classifier ("No WGS error suppression). All quality filters and recurrent artifact filters were conserved between the two approaches. Fragment-level classification with MRD-EDGE^{SNV} significantly improved sensitivity vs. controls in preoperative stage III CRC plasma samples (**Supplementary Fig. 12**).

Fragment-level variability for non-cancer (control) samples. We performed several analyses to demonstrate generalizability between non-cancer (control) populations at the fragment level for MRD-EDGE^{SNV}. In our NovaSeq stage III perioperative CRC cohort, our ctDNA detection threshold of 95% specificity against held-out controls was highly conserved among 4 control noise distributions including: (i) Aarhus controls (95.0% in *n*=40 controls, 5 controls were held out for CRC SNV model training, sequenced on Illumina NovaSeq with 1.5 flow cells at Aarhus University), (ii) NYGC controls (94.9% in *n*=35 controls, sequenced on Illumina NovaSeq with v1.0 flow cells at the New York Genome Center), (iii) HiSeq controls, (95.4% in *n*=38 controls, sequenced on Illumina HiSeq X at the New York Genome Center), and (iv) a cross-patient noise distribution (95.2% *n*=14 cross patient controls from patients with stage III colorectal cancer, sequenced on Illumina NovaSeq with v1.5 flow cells at Aarhus University, **Supplementary Fig. 13**). Therefore, applying the prespecified Z score threshold defined using the NovaSeq stage III CRC cohort provided highly conserved estimates of the sensitivity (100%) and specificity (~95%) when investigated in 4 different control noise distributions. This indicates that MRD-EDGE^{SNV} sample classification is highly generalizable with different control cohorts. Furthermore, our

analysis indicates that future implementations of MRD-EDGE^{SNV} are unlikely to need a new panel of control samples at every application of the platform, though this will have to be confirmed in future studies. We further found broadly similar side-by-side trends for detection rate noise distributions for each patient-specific mutation profile (*n*=15) (**Supplementary Fig. 14a**) and detection rate variance (**Supplementary Fig. 14b**).

As a further evaluation of MRD-EDGE^{SNV} generalizability, we assessed the performance of our MRD-EDGE^{SNV} platform on HiSeq cancer samples against non-cancer control plasma samples sequenced on 2 different sequencing platforms. We applied our prespecified NovaSeq stage III perioperative CRC cohort Z score detection threshold (95% specificity against held-out controls from the same center and sequencing platform), to the same controls used in the stage III CRC analysis (Aarhus controls, *n*=40), as well as the HiSeq controls (*n*=38) as described above in "**Application of tumor-informed MRD-EDGE to HiSeq re-analysis cohorts**". We found similar AUC when either set of cohorts was used as the noise distribution for the patient-specific SNV profiles (Aarhus controls AUC 0.97, 95% CI: 0.92 - 1.00, HiSeq controls AUC 0.98, 95% CI: 0.95 - 1.00; **Supplementary Fig. 15**). The prespecified 95% specificity threshold from our NovaSeq stage III CRC analysis reflected a Z Score specificity of 0.963 in Aarhus controls and 0.957 in HiSeq controls.

Evaluating for fragment-level biases due to sequencing batch. We evaluated potential batch effects related to DNA extraction date, library preparation date and sequencing date in MRD-EDGE^{SNV} sample level classification. We performed an analysis of variance (ANOVA) on neoadjuvant NSCLC plasma, as these samples were processed in our laboratory at different timepoints over two years (July 2020 to May 2022). No significant differences were found for extraction, library preparation dates, or sequencing dates. However, time of collection within treatment course, such as whether a sample was drawn prior to treatment, during radiation, or postoperatively, produced statistically significant differences in the prediction of MRD-EDGE^{SNV} Z

score (P=0.014, Supplementary Table 16), which conforms to our expectation of changing plasma TF throughout treatment. Standard checking plots are included as **Supplementary Fig. 16**.

Evaluating the role of sequencing batch in MRD-EDGE^{SNV} performance. We performed a series of training experiments on the melanoma classifier, in which cases and controls are sequenced in the same batch, to evaluate whether training batch in the positive or negative label confounds results. We compared our original training paradigm to a series of different control batches (**Supplementary Fig. 17**). We found that the sequencing batch of the negative label (whether on same batch or different batches) did not significantly affect model performance, as validation accuracy scores remained similar for each group. As a negative control, we trained a model in which the positive and negative labels in training are from separate batches (as in experiment two). However, in the validation set, the positive and negative labels are both derived from control samples. The validation positive labels are non-cancer controls from the same batch as the melanoma sample in the training positive label, and the negative labels are from the same batch as the training negative label. Therefore, if the model learned technical features of the positive label batch or the negative label batch, we would expect the validation set to show performance above noise. Instead, validation accuracy approached 0.5, suggesting that the model does not learn significant differences between control batches (**Supplementary Fig. 17**).

Read depth PON generalizability. To ensure generalizability of read-depth PONs among control samples, we performed random sampling of plasma samples in the PON vs. held-out of the PON and evaluated results in pretreatment, preoperative plasma samples from our neoadjuvant immunotherapy and SBRT NSCLC cohort. Compared to results from our original PON (**Extended Data Fig. 4a**), we saw no significant differences in preoperative sensitivity or AUC performance (**Supplementary Data Fig. 18**).

Evaluation of drop-out rate and training sample selection in MRD-EDGE^{SNV}. To mitigate overfitting, we locked our model at training and validated performance in held-out validation and

test sets for each cancer type (Supplementary Table 1). We further performed a sparsity analysis in which we evaluated accuracy at different dropout rates, which randomly drop nodes within neural networks to reduce overfitting[87], in our melanoma held-out validation set. Here, we found that our dropout rate of 0.5 appeared to be appropriately fit (not under or overfit) for optimal performance (**Supplementary Fig. 19a**). Finally, we performed random sampling with replacement in CRC to confirm that our number of training samples was poised for optimal performance. We found that performance (as measured by classification accuracy) in our fragment-based training paradigm plateaued at 4 or higher positive label training samples or 150,000 total ctDNA fragments, suggesting that training with a small number of clinical samples is appropriate due to the large number of fragments in high-burden disease (**Supplementary Data Fig. 19b**).

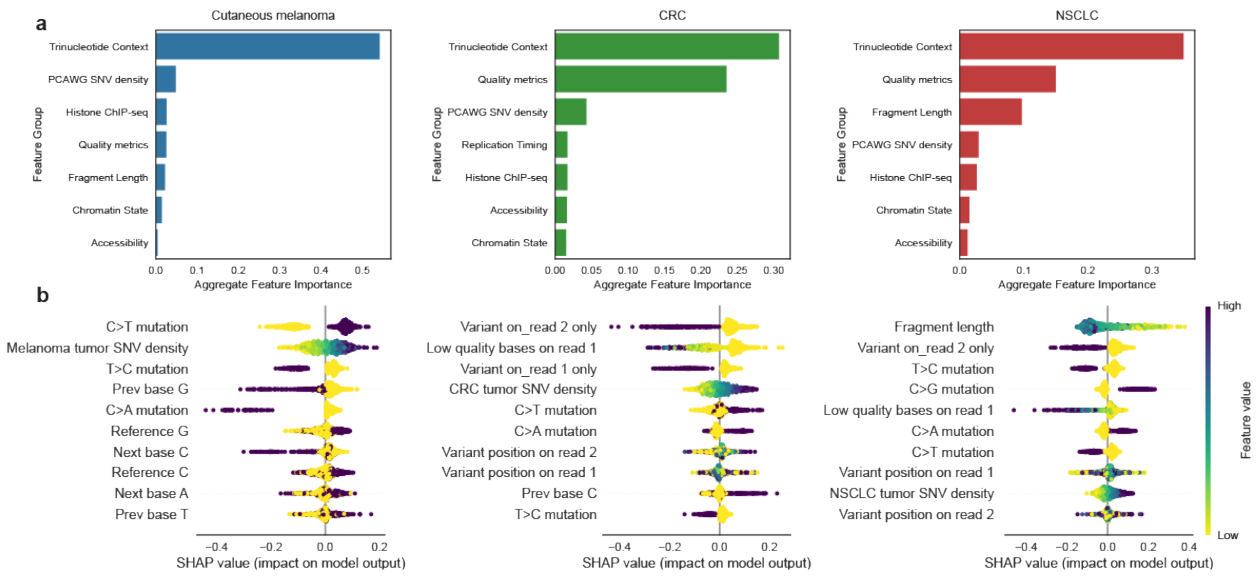

Supplementary Fig. 1: Shapley feature importance for MRD-EDGE^{SNV} in different tumor types

a) Shapley feature importance plots for MRD-EDGE^{SNV} features in (left) cutaneous melanoma (middle) CRC, and (right) NSCLC. SNV model features were converted to tabular features for Shapley evaluation. Feature groups were aggregated through sum of mean feature importance to determine category-level aggregate feature importance. **B**) Top ten individual Shapley features in (left) cutaneous melanoma (middle) CRC, and (right) NSCLC ordered according to importance (impact on model output). Each X-axis point is a Shapley value (Methods) for a feature within the neural network at a given feature value. Color represents the value of the feature from low to high.

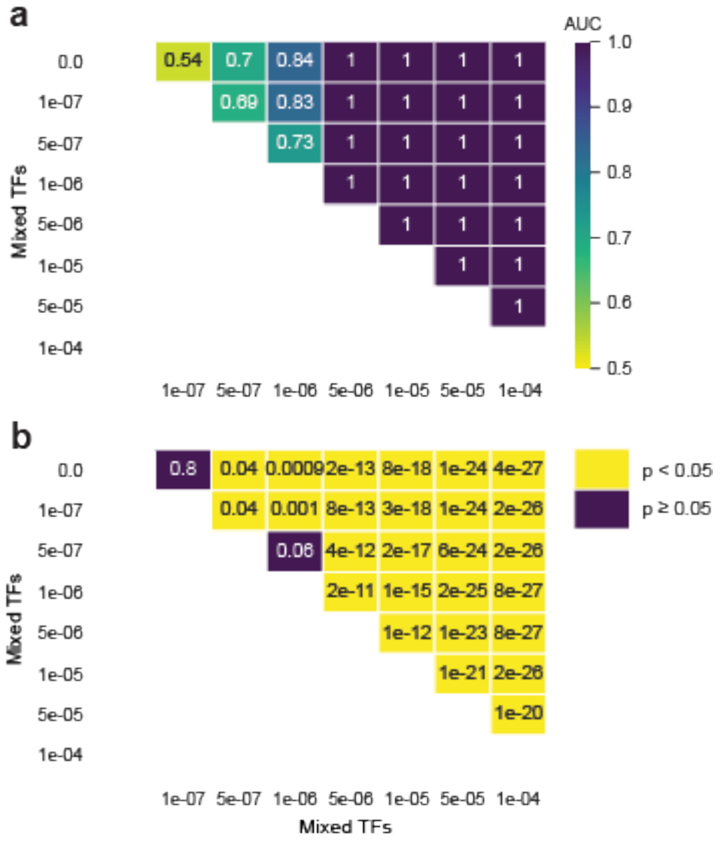

Supplementary Fig. 2: Discriminating *in silico* mix fractions with MRD-EDGE^{SNV}

In silico studies of cfDNA from the metastatic cutaneous melanoma sample MEL-100 mixed into cfDNA from a healthy plasma sample (CTRL-216) at mixing fractions TF = 10^{-7}–10^{-4} at 16X coverage depth, performed in 20 technical replicates with independent sampling seeds. **a)** An AUC heatmap benchmarks discrimination between different mixed TFs as measured by MRD-EDGE^{SNV} detection rate. **b)** A P-value heatmap benchmarks significant differences between detection rates at different mixed TFs (two-sided Student's t-test).

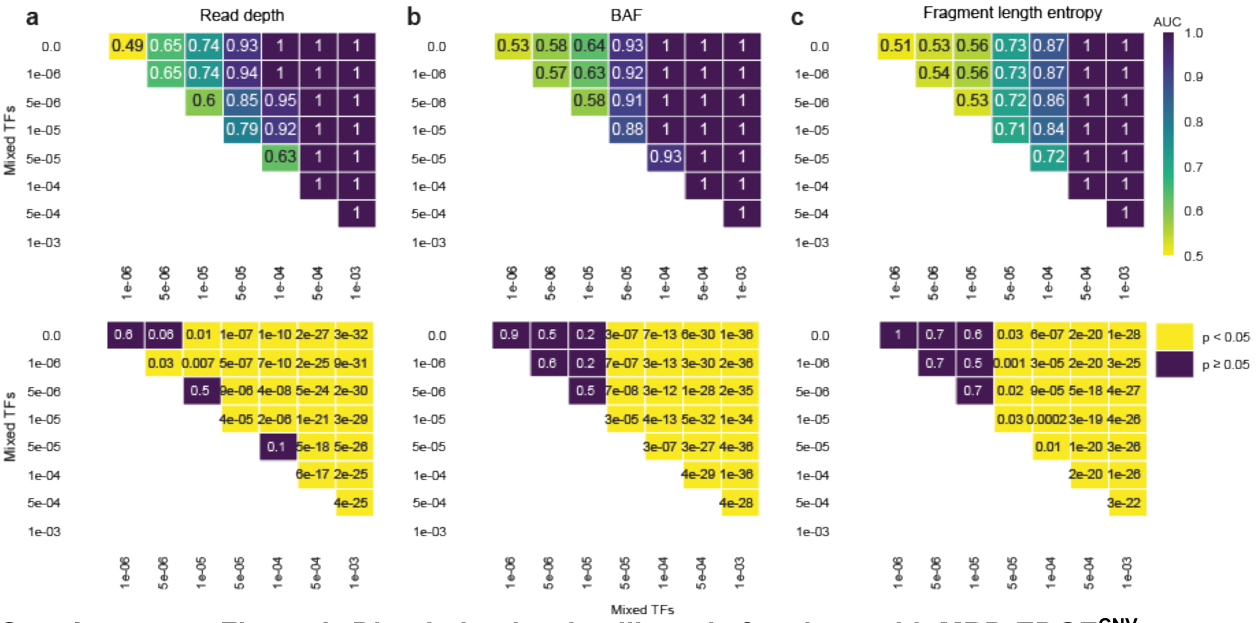

Supplementary Figure 3: Discriminating *in silico* mix fractions with MRD-EDGE^{CNV}

In silico studies of cfDNA from the metastatic colorectal cancer sample CRC-930 mixed into cfDNA from a healthy plasma sample (CTRL-443) at mixing fractions TF = 10^{-6}–10^{-3} at 29X coverage depth, performed in 25 technical replicates with independent sampling seeds for read depth (**a**), BAF (**b**), and fragment length entropy (**c**) classifiers. Top) An AUC heatmap benchmarks discrimination between different mixed TFs. Bottom) A P-value heatmap benchmarks significant differences between read depth, BAF, and fragment length entropy signal at different mixed TFs (two-sided Student's t-test).

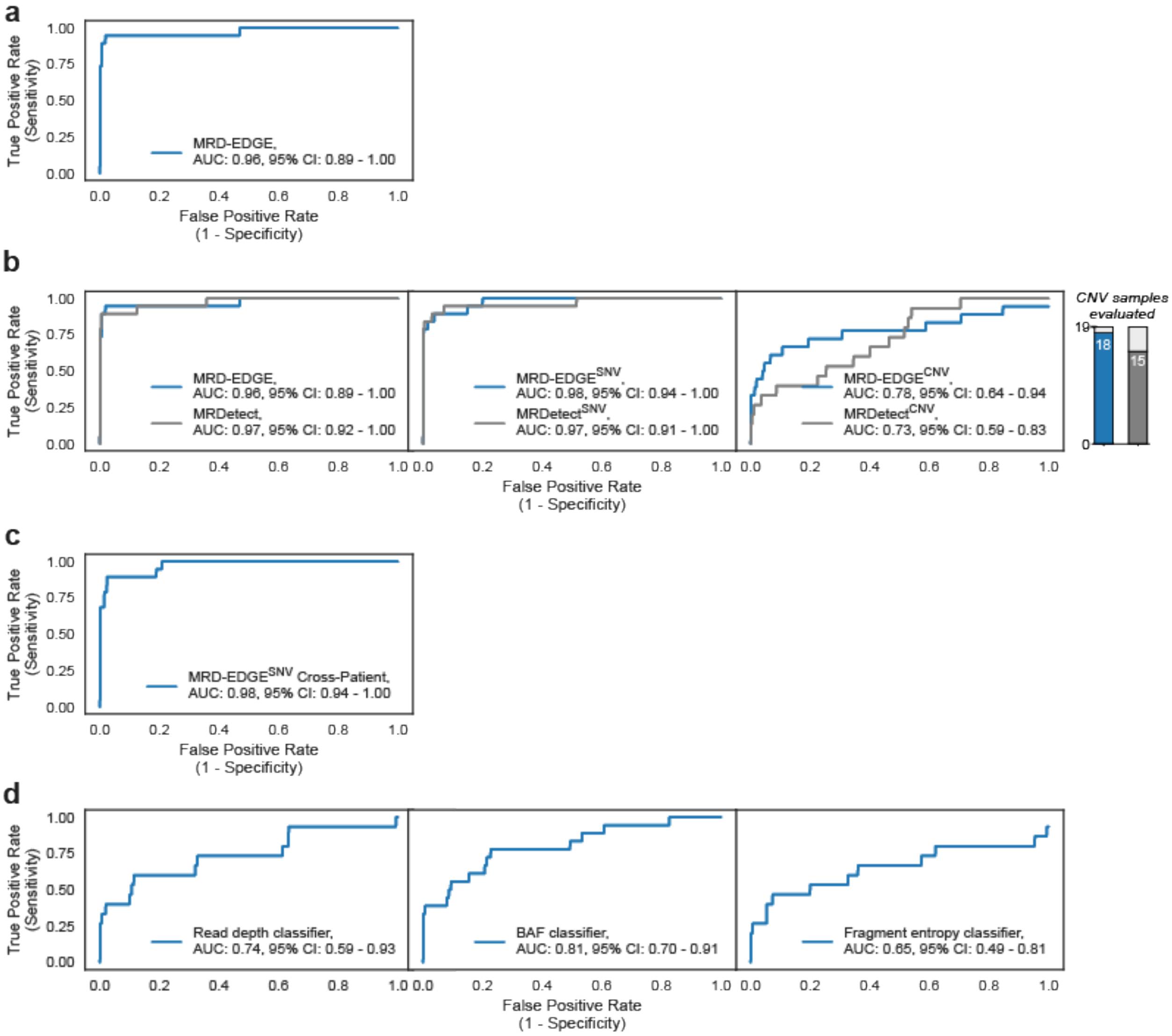

Supplementary Figure 4: MRD-EDGE preoperative performance in colorectal cancer plasma sequenced with Illumina HiSeq X (HiSeq CRC cohort)

a) ROC analysis on MRD-EDGE (combined detection model of SNV and CNV mutations) in pretreatment early-stage colorectal cancer. Preoperative plasma samples with matched tumor mutation profiles (*n*=19, Supplementary Table 5) are compared with control plasma samples assessed against all unmatched HiSeq CRC tumor mutation profile (*n*=15 tumor profiles assessed across 10 control samples from HiSeq controls cohort, *n*=190 control-comparisons). Twenty-eight control samples used in the HiSeq read depth panel of normals were withheld from downstream analysis. **b)** (left) ROC analysis for MRD-EDGE (blue) as detailed in (**a**) and MRDetect (gray), a

composite of MRDetect^{SNV}and MRDetect^{CNV}. For MRDetect, preoperative plasma samples with matched tumor mutation profiles (*n*=19, Supplementary Table 5) are compared against control plasma samples assessed against all unmatched HiSeq CRC tumor mutation profile (*n*=19 tumor profiles assessed against 29 controls from HiSeq controls, *n*=551 comparisons). Nine control samples used in the MRDetect^{CNV} panel of normals were withheld from downstream analysis (Supplementary Table 14). (middle) ROC analysis on preoperative HiSeq colorectal SNVs for MRD-EDGE^{SNV} (blue) and MRDetect^{SNV} (gray). Preoperative plasma samples with matched tumor mutation profiles (*n*=19, Supplementary Table 5) are compared with control plasma samples assessed against all unmatched HiSeq CRC tumor mutation profiles (for MRD-EDGE, 19 mutation profiles assessed across 38 control samples for *n*=722 control-comparisons; for MRDetect, 19 mutation profiles assessed across 29 control samples for *n*=551 control-comparisons). (right) ROC analysis on preoperative colorectal CNVs for MRD-EDGE^{CNV} (blue) and MRDetect^{CNV} (gray). Preoperative plasma samples (*n*=18 for MRD-EDGE^{CNV} with 1 sample excluded due to insufficient aneuploidy; *n*=15 for MRDetect, 4 samples excluded due to insufficient aneuploidy) with matched tumor mutation profiles are compared with control plasma samples assessed against all HiSeq CRC tumor mutation profiles (*n*=18 tumor profiles assessed across 10 control samples from HiSeq controls cohort, *n*=180 control-comparisons). Twenty-eight samples from HiSeq controls included in the read depth classifier panel of normal samples were held out from the MRD-EDGE^{CNV} ROC analysis. **c**) Cross-patient ROC analysis on HiSeq CRC plasma samples demonstrates similar performance to control (non-cancer) plasma ROC analysis. Preoperative plasma samples (*n*=19) with matched tumor mutation profiles are compared with HiSeq CRC plasma samples assessed against all unmatched HiSeq CRC tumor profiles (*n*=19 tumor profiles assessed across 18 cross-patient samples, *n*=342 cross-comparisons) **d)** ROC analysis performed on CNV-based Z-score values for read depth (left), BAF (middle), and fragment length entropy (right) CNV classifiers in preoperative HiSeq CRC. Preoperative plasma samples with matched tumor profiles (*n*=15 for read depth and fragment length entropy, *n*=18 for BAF) are compared with control plasma samples assessed against all unmatched tumor profiles (*n*=150 comparisons for read depth, 15 tumor profiles assessed across 10 control samples; *n*=684 comparisons for BAF, 18 mutation profiles assessed across 38 control samples; *n*=570 comparisons for fragment length entropy, 15 tumor profiles assessed across 38 control samples). Twenty-eight control samples included in the read depth panel of normal samples were withheld from read-depth analysis.

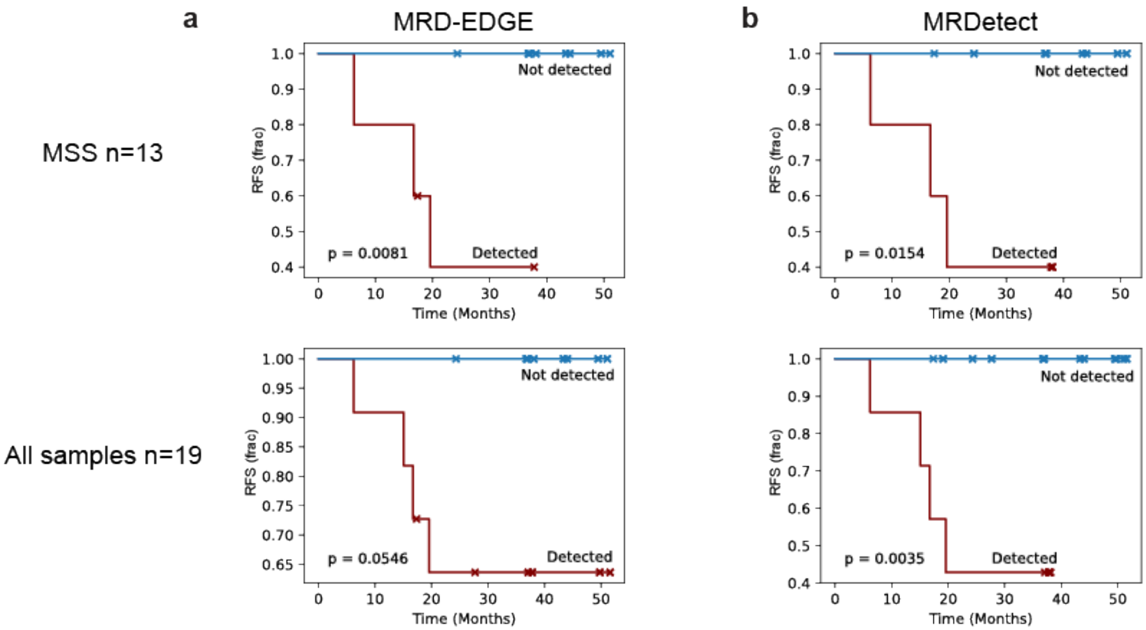

Supplementary Fig. 5: Postoperative MRD detection in HiSeq CRC

a) (top) Kaplan–Meier disease-free survival analysis was performed for MRD-EDGE across patients with detected (*n*=5) and non-detected (*n*=8) postoperative ctDNA in MSS HiSeq colorectal samples (*n*=13). Postoperative ctDNA detection was associated with shorter recurrence-free survival (two-sided log-rank test). (bottom) Survival analysis was performed on all HiSeq CRC patients (*n*=6 patients with MSI tumors and *n*=13 patients with MSS tumors) with detected (n=11) and non-detected (*n*=8) postoperative ctDNA. Association between postoperative ctDNA detection and shorter recurrence-free survival was not statistically significant (*P*=0.0546, two-sided log-rank test). **b**) The same survival analyses were performed with MRDetect per published results[14] including one sample that recurred in subsequent follow up. (top) Survival analysis was performed on patients with detected (*n*=5) and non-detected (*n*=8) postoperative ctDNA in MSS HiSeq colorectal samples (*n*=13). Postoperative ctDNA detection was associated with shorter recurrence-free survival (two-sided log-rank test). (bottom) Survival analysis was performed on all patients (*n*=19) with detected (*n*=7) and non-detected (*n*=12) postoperative ctDNA. Postoperative ctDNA detection was associated with shorter recurrence-free survival (two-sided log-rank test). Adjustments were not made for multiple comparisons. MSS, microsatellite stable. MSI, microsatellite instability.

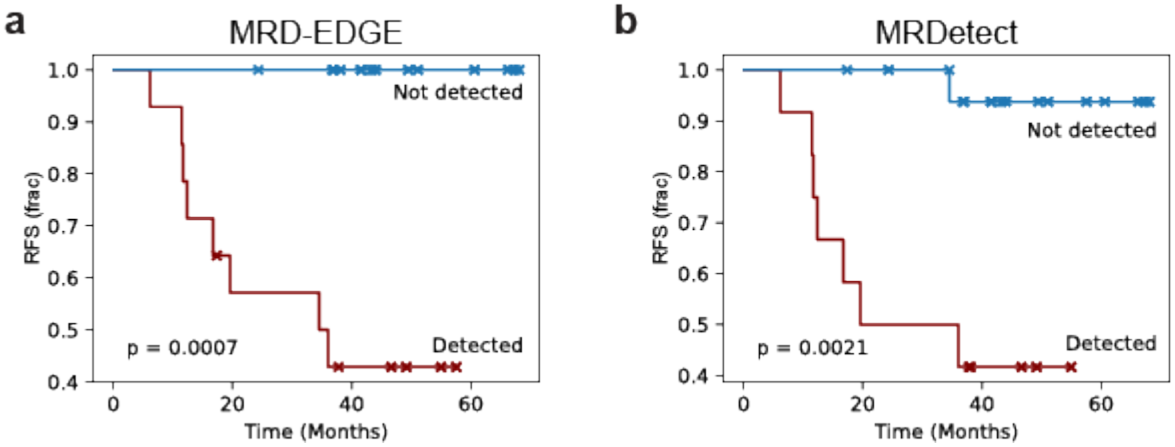

Supplementary Fig. 6: Postoperative MRD detection in combined CRC and stage III CRC cohorts

a) Kaplan–Meier disease-free survival analysis for MRD-EDGE in combined HiSeq CRC and NovaSeq stage III CRC cohorts was performed over all patients with MSS tumors with detected (*n*=14) and non-detected (*n*=14) postoperative ctDNA. **b**) Kaplan–Meier disease-free survival analysis for MRDetect in the same patients was performed over patients with detected (*n*=12) and non-detected (*n*=16) postoperative ctDNA. Postoperative ctDNA detection was associated with shorter recurrence-free survival (two-sided log-rank test) for both platforms. MSS, microsatellite stable.

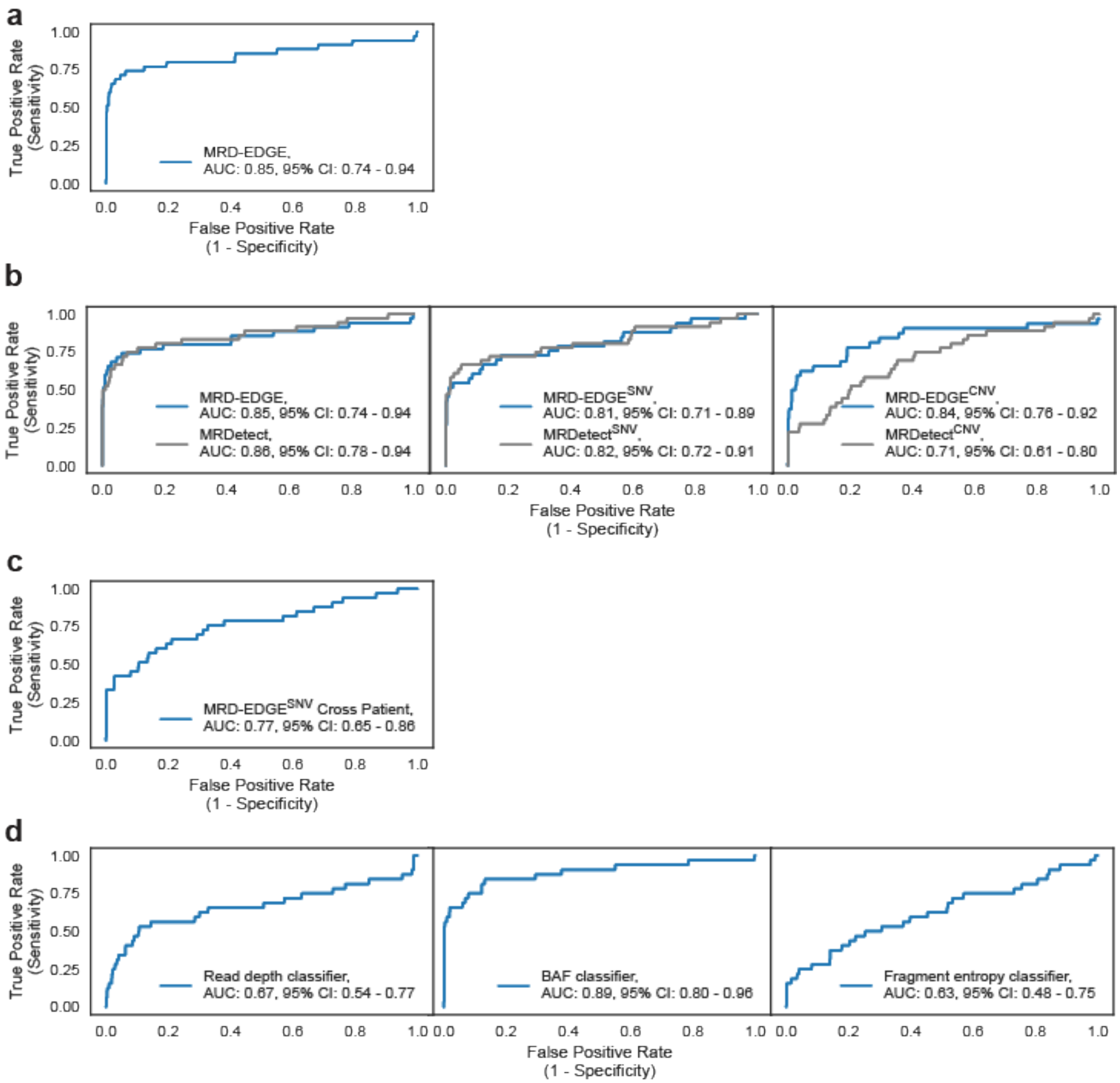

Supplementary Fig. 7: Re-analysis of HiSeq NSCLC data with MRD-EDGE

a) ROC analysis on MRD-EDGE (combined detection model of SNV and CNV mutations) in pretreatment early-stage NSCLC. Preoperative plasma samples with matched tumor mutation profiles (*n*=35, Supplementary Table 5) are compared with control plasma samples assessed against all unmatched HiSeq CRC tumor mutation profile (*n*=15 tumor profiles assessed across 10 control samples from HiSeq controls cohort, *n*=350 control-comparisons). Twenty-eight control samples used in the HiSeq read depth panel of normals were withheld from downstream analysis.

b) (left) ROC analysis for MRD-EDGE (blue) as detailed in (**a**) and MRDetect (gray), a composite of MRDetect^{SNV}and MRDetect^{CNV}. For MRDetect, preoperative plasma samples with matched tumor mutation profiles (*n*=35, Supplementary Table 5) are compared against control plasma samples assessed against all unmatched HiSeq NSCLC tumor mutation profile (*n*=36 tumor profiles assessed against 29 controls from HiSeq controls, *n*=1,044 comparisons). Nine control samples used in the MRDetect^{CNV} panel of normals were withheld from downstream analysis. (middle) ROC analysis on preoperative HiSeq NSCLC SNV mutation profiles for MRD-EDGE^{SNV} (blue) and the MRDetect^{SNV} SVM (gray). Preoperative plasma samples samples with matched tumor mutation profiles (*n*=33 for MRD-EDGE^{SNV}, 3 samples were excluded due to an absence of high-confidence SNVs in tumor tissue due to low tumor purity; *n*=36 for MRDetect) are compared with control plasma samples assessed against all unmatched HiSeq NSCLC tumor mutation profiles (for MRD-EDGE^{SNV}; 33 mutation profiles assessed across 38 HiSeq control samples, *n*=1,254 comparisons; for MRDetect^{SNV} SVM; 36 mutation profiles assessed across 29 HiSeq control samples, *n*=1,044 comparisons). For MRDetect, 9 controls used to train the MRDetect^{CNV} CNA panel of normals were excluded from downstream analysis. (right) ROC analysis on preoperative NSCLC CNVs for MRD-EDGE^{CNV} (blue) and MRDetect^{CNV} CNA (gray). Preoperative plasma samples with matched tumor mutation profiles (*n*=32 for MRD-EDGE^{CNV}; 2 samples were excluded due to insufficient aneuploidy and 2 samples were excluded due to the absence of a matched normal sample; *n*=36 for MRDetect) are compared with control plasma samples assessed against all unmatched HiSeq NSCLC tumor mutation profiles. Twenty-eight samples from HiSeq controls included in the read depth classifier panel of normal samples were held out from the CNV ROC analysis. **c**) Cross-patient ROC analysis on HiSeq NSCLC plasma samples demonstrates similar performance to control (non-cancer) plasma ROC analysis. Preoperative plasma samples (*n*=33) with matched tumor mutation profiles are compared with HiSeq NSCLC plasma samples assessed against all unmatched HiSeq NSCLC tumor profiles (33 mutation profiles assessed across 35 cross-patient samples, *n*=1,260 cross-comparisons). **d)** ROC analysis performed on CNV-based Z-score values for read depth (left), BAF (middle), and fragment length entropy (right) CNV classifiers in preoperative HiSeq NSCLC. Preoperative plasma samples with matched tumor profiles (*n*=32) are compared with control plasma samples assessed against all unmatched tumor profiles (*n*=320 comparisons for read depth, 32 tumor profiles assessed across 10 control samples; *n*=1,216 comparisons for BAF and fragment length entropy, 32 mutation profiles assessed across 38 control samples). Twenty-eight control samples included in the read depth panel of normal samples were withheld from read-depth analysis.

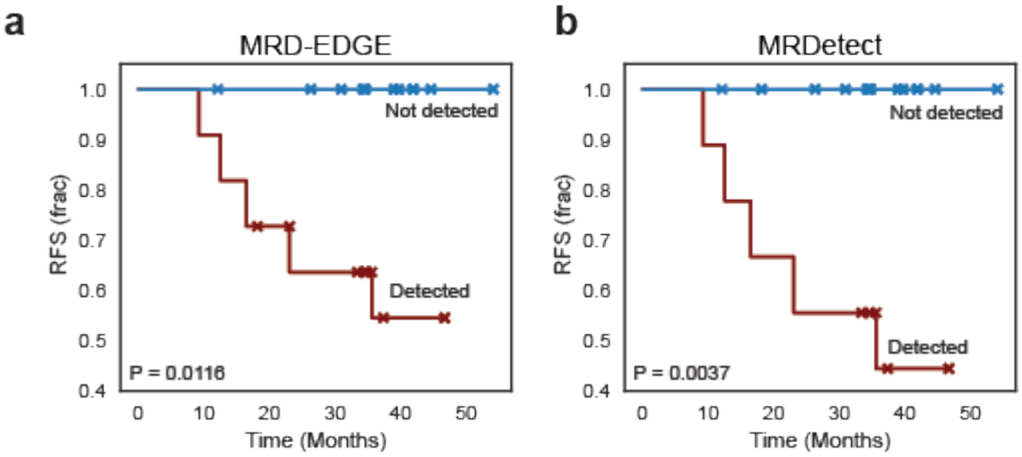

Supplementary Fig. 8: Re-analysis of previous NSCLC data accounting for updated results with MRD-EDGE.

Kaplan–Meier disease-free survival analysis was performed over all patients with detected and non-detected postoperative ctDNA for MRD-EDGE (**a**) and MRDetect (**b**). Postoperative ctDNA detection showed association with shorter recurrence-free survival (two-sided log-rank test) for both platforms. Results were updated to account for one additional recurrence in extended follow up. This sample (NSCLC-111, Supplementary Table 6) was detected by both MRD-EDGE and MRDetect.

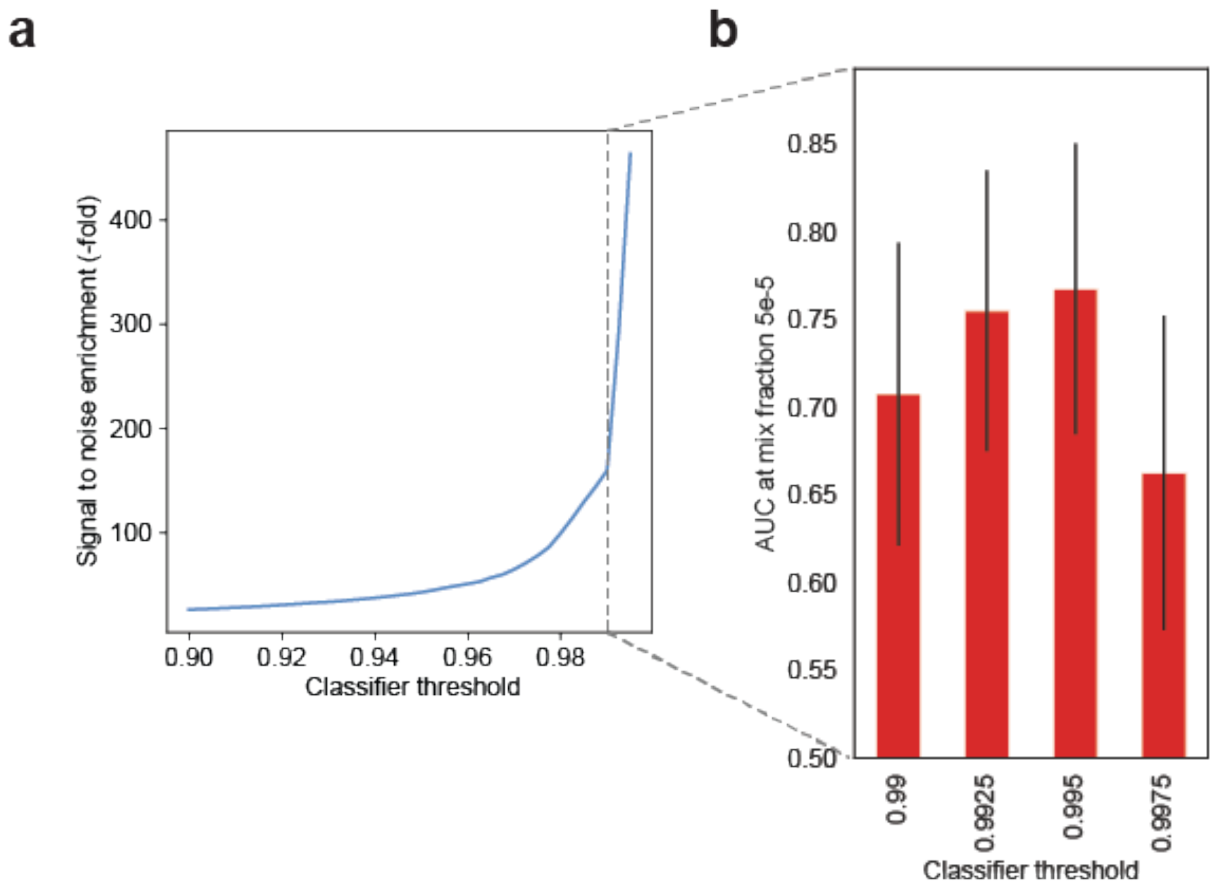

Supplementary Fig. 9: Determination of MRD-EDGE *de novo* mutation calling classification threshold

a) Fragment-level signal-to-noise enrichment, defined as the fraction of remaining ctDNA fragments (signal) over remaining cfDNA SNV artifacts (noise), for different MRD-EDGE^{dnSNV} classification thresholds in the melanoma held-out validation set derived from tumor-confirmed ctDNA SNVs from the melanoma patient MEL-100 and post-quality filtered cfDNA artifacts from healthy control plasma (Supplementary Table 1). The MRD-EDGE^{SNV} deep learning classifier uses a sigmoid activation function that outputs the likelihood between 0 and 1 that a candidate SNV fragment is a mutated ctDNA fragment or cfDNA harboring a sequencing error, and the classification threshold is used as a decision boundary for these two classes. Signal-to-noise enrichment increases at higher classification thresholds, as expected. **b)** As increased specificity will ultimately eliminate most of the signal, to choose an optimal threshold for classification, we

compared sensitivity vs. TF=0 in an *in silico* study of cfDNA from the metastatic melanoma sample MEL-100 mixed in *n*=20 replicates against cfDNA from a healthy plasma sample (TF=0) at $5*10^{-5}$ at 16X coverage depth. We found optimal performance at a classifier threshold of 0.995 as measured by AUC of mixed replicates against TF=0. This threshold was subsequently applied in *de novo* mutation calling analyses. Error bars indicate Delong AUC variance.

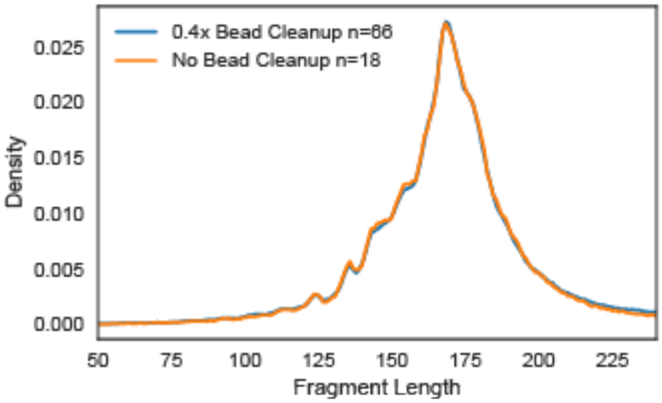

Supplementary Fig. 10: Fragment size distribution for melanoma samples +/- bead cleanup

Fragment size distribution for melanoma samples that did and did not undergo bead cleanup. A subset of melanoma plasma samples (blue, *n*=66) stored in an immunotherapy biobank underwent 0.4x magnetic bead cleanup to remove contamination. No differences were seen in fragment length distribution compared to samples from the same cohort that did not undergo cleanup (orange, *n*=18). Fragment size was estimated from paired-end sequencing.

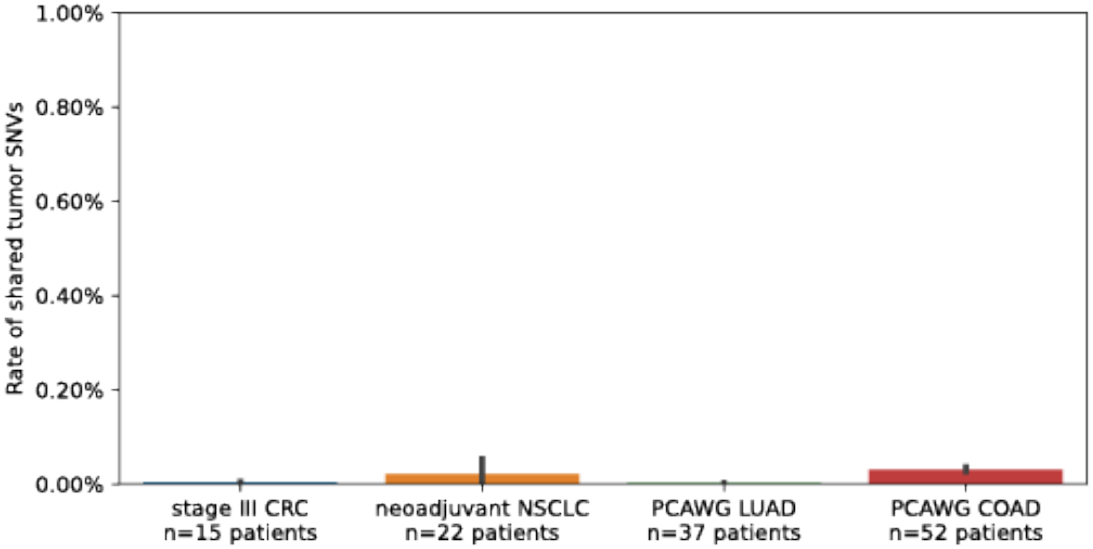

Supplementary Figure 11: Rate of shared SNVs between WGS tumor samples.

Rate of shared tumor SNVs between any 2 samples in 4 WGS cohorts: stage III CRC (n=15 patients, median rate=0, mean=$4*10^{-5}$), neoadjuvant NSCLC (n=22, 0, $2*10^{-4}$), PCAWG LUAD cohort (n=37, 0, $2*10^{-5}$), and PCAWG COAD (n=52, $6*10^{-5}$, $3*10^{-3}$). Error bars indicate 95% CI.

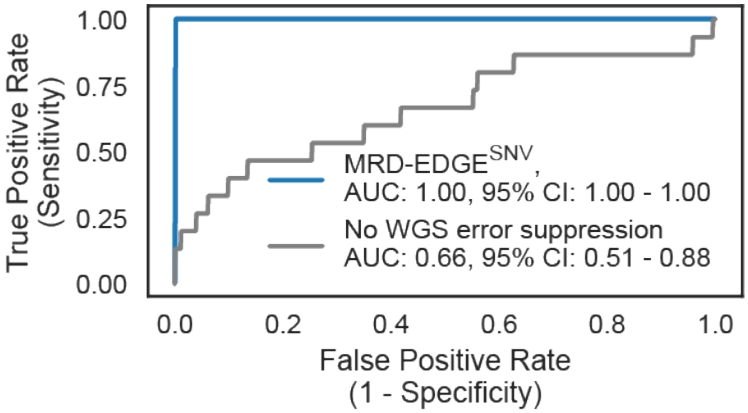

Supplementary Figure 12: Impact of individual fragment classification on MRD-EDGE^{SNV} performance in preoperative stage III colorectal cancer

ROC analysis with MRD-EDGE^{SNV} (blue), and without WGS error suppression (gray) in stage III CRC cohort. Preoperative plasma samples (*n*=15) were used as the true label (Supplementary Table 5). Control plasma samples (*n*=40) from the Aarhus controls cohort assessed against all stage III CRC tumor mutation profiles (*n*=15) were used as the false label (*n*=600 comparisons). Five control samples included in SNV model training were withheld from this analysis (Supplementary Table 14).

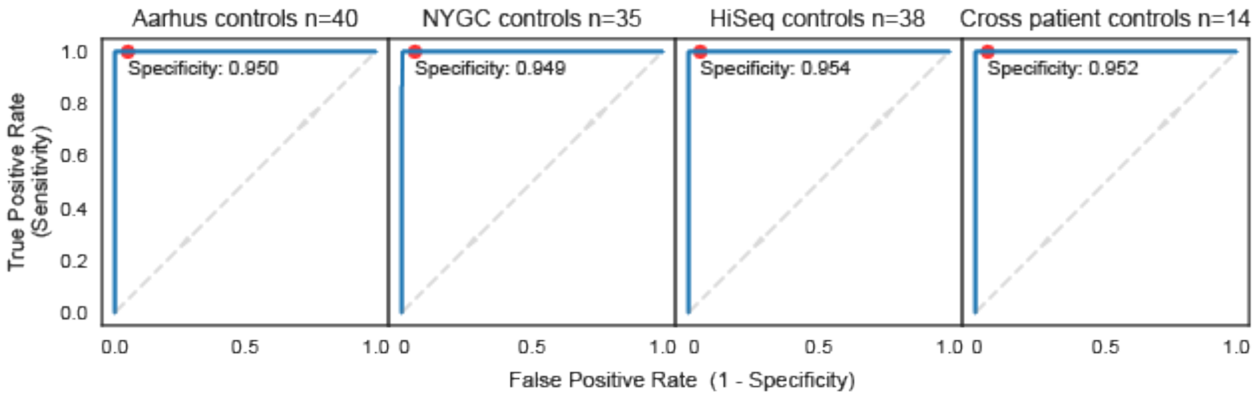

Supplementary Fig. 13: MRD-EDGE^{SNV} Z scores compared to 4 non-cancer control plasma cohorts

ROC analysis on preoperative colorectal SNV mutation profiles for MRD-EDGE^{SNV} (blue) vs. noise distributions from different sequencing centers and sequencing platforms. Preoperative stage III colorectal plasma samples (*n*=15) were used as the true label, and the panel of control plasma samples assessed against all stage III CRC tumor mutation profiles was used as the false label. Aarhus controls (*n*=40) that were sequenced at the same sequencing center (Aarhus University) and the same sequencing platform (NovaSeq) were used as the baseline noise distribution. The 95.0% specificity threshold is marked in red in the other noise distributions: NYGC controls, sequenced on Illumina NovaSeq at the New York Genome Center (94.9% specificity); HiSeq controls, sequenced on Illumina HiSeq X at the New York Genome Center (95.4% specificity); and cross-patient controls from other stage III CRC patients from the same center and sequencing platform (specificity 95.2%).

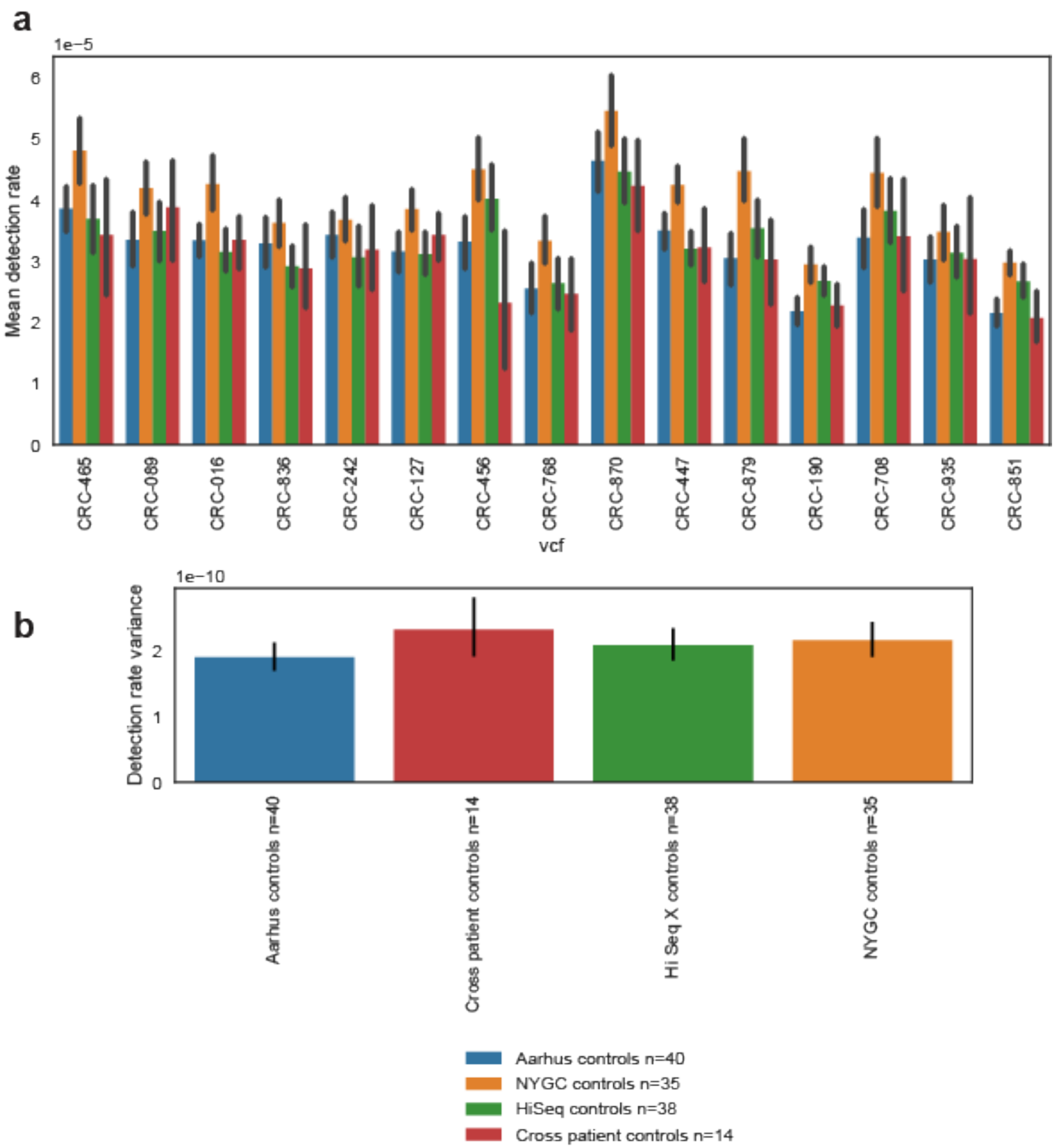

Supplementary Fig. 14: Mean detection rate and variance in 4 non-cancer control cohorts

a) Side-by-side comparison of mean detection rates in non-cancer (control) noise distributions for 15 stage III CRC patient-specific SNV mutation profiles. Whiskers represent standard error for detection rate for each control noise distribution. **b**) Detection rate variance for each control noise distribution. Error bars indicate Bayesian 95% confidence interval for population variance.

Supplementary Fig. 15, Widman et al.

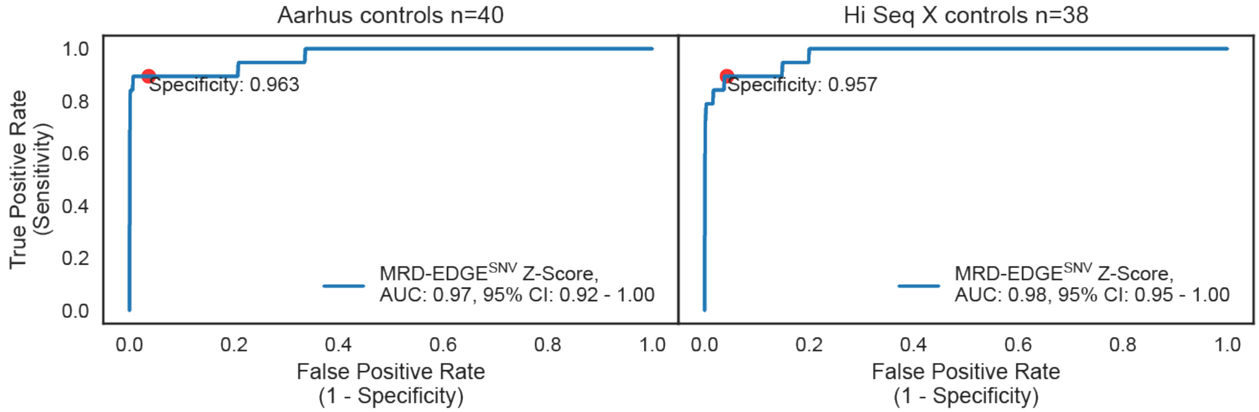

Supplementary Figure 15: Comparison of non-cancer control plasma samples sequenced on 2 different sequencing platforms in preoperative early-stage colorectal cancer re-analysis cohort

ROC analysis on preoperative HiSeq colorectal SNV mutation profiles for MRD-EDGE^{SNV} (blue) vs. noise distributions from different sequencing centers and sequencing platforms. Preoperative early-stage colorectal plasma samples (*n*=19) re-analyzed from prior work[14] were used as the true label, and the panel of control plasma samples assessed against all HiSeq CRC tumor mutation profiles was used as the false label. The Z Score ctDNA detection threshold was prespecified in the stage III CRC cohort (Fig. 3a-b). The threshold is marked in red for two noise distributions: Aarhus controls (*n*=40), sequenced on Illumina NovaSeq at Aarhus University (96.3% specificity), and HiSeq controls, sequenced on Illumina HiSeq X at the New York Genome Center (95.7% specificity). Preoperative ctDNA sensitivity is 89.5% (17/19 samples detected above the threshold) when either control cohort is used as the control noise distribution.

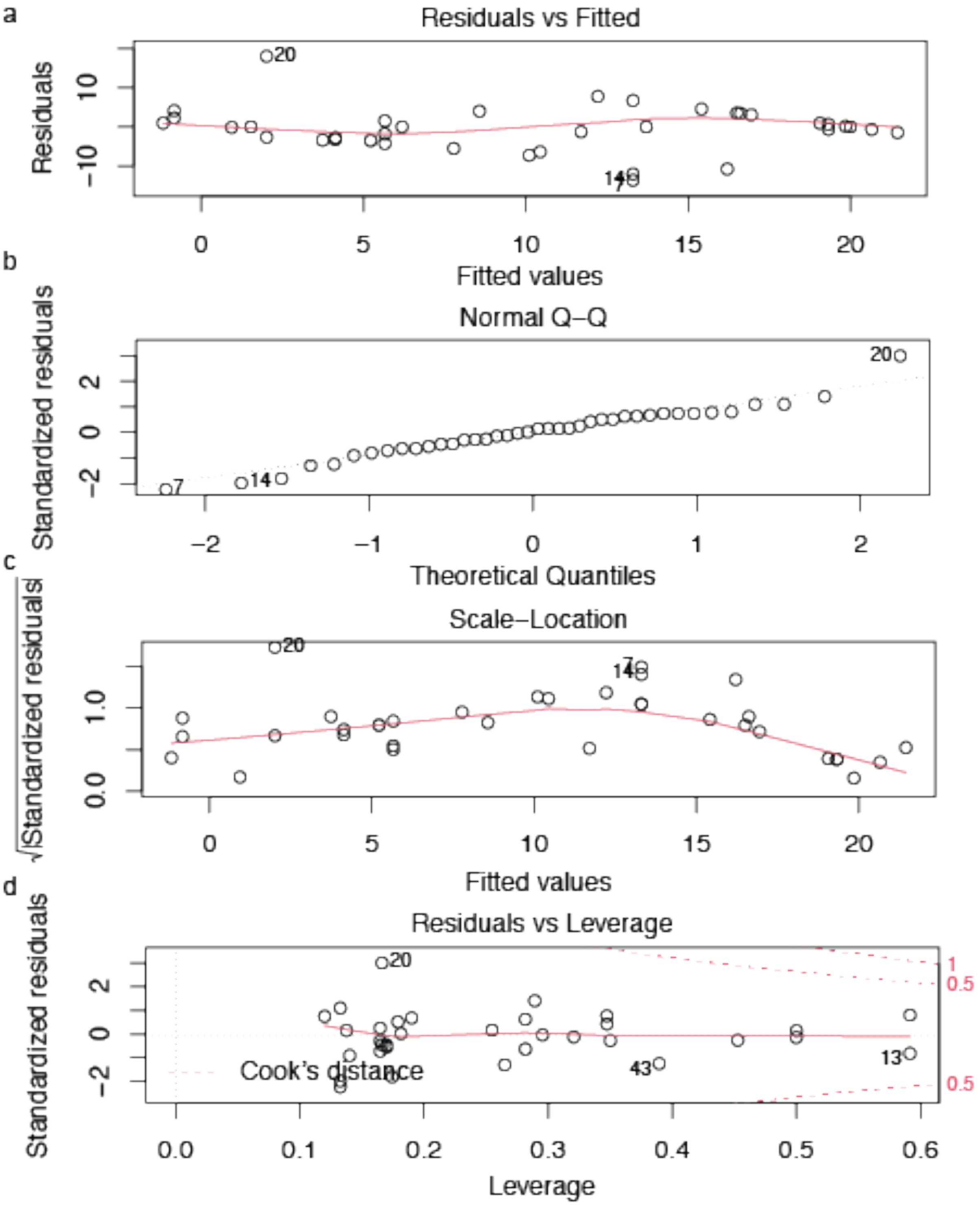

Supplementary Figure 16: ANOVA model checking plots.

Residuals vs. Fitted, Normal Q-Q, Scale Location, and Residuals vs. Leverage plots for two-way ANOVA for relationship between categorical variables and MRD-EDGE^{SNV} Z score in neoadjvuvant NSCLC (*n*=44) cancer samples. ANOVA was performed using stats package in R to model the continuous variable MRD-EDGE^{SNV} Z score as the dependent variable and the variables ‘DNA extraction date’, ‘Library Preparation Data’, ‘Sequencing date’, and ‘Timepoint’ as independent variables. MRD-EDGE^{SNV} Z Scores were capped at 20 to exclude outliers. **a**) residuals vs fitted plot, x-axis is fitted values from the model (Predicted values), y-axis is residuals (Difference between observed and predicted values). **b**) normal Q-Q Plot: x-axis is theoretical quantiles from a standard normal distribution, y-axis is ordered residuals from the model (Quantiles of the residuals). **c**) scale-location plot: x-axis is fitted values from the model (Predicted values), y-axis is square root of standardized residuals. **d**) residuals vs leverage plot: x-axis is leverage values (Measure of influence of each data point on the model), y-axis is standardized residuals (Measure of how far each observed value is from the expected value). Plots were constructed from R stats package.

a

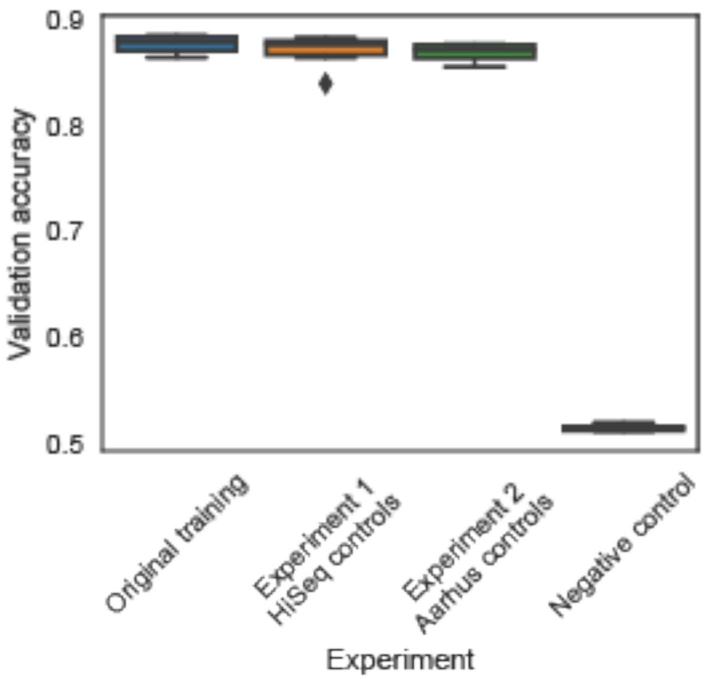

b

**Approach**	**Training set positive label**	**Training set negative label**	**Validation set positive label**	**Validation set negative label**
Original training (positive and negative labels from same batch)	NovaSeq **Melanoma**, batch 2020-08-25	NYGC **controls**, batch 2020-08-25	HiSeq **Melanoma**, batch 2019-05-22	NYGC **controls**, batch 2020-08-25
Experiment 1 (positive and negative labels from different batches)	NovaSeq **Melanoma**, batch 2020-08-25	HiSeq **controls**, batch 2018-04-26	HiSeq **Melanoma**, batch 2019-05-22	HiSeq **controls**, batch 2018-04-26
Experiment 2 (positive and negative labels from different batches)	NovaSeq **Melanoma**, batch 2020-08-25	Aarhus **controls**, batch 2021-02-10	HiSeq **Melanoma**, batch 2019-05-22	Aarhus **controls**, batch 2021-02-10
Negative control (positive and	NovaSeq **Melanoma**,	Aarhus **controls**, batch	NYGC **controls**, batch 2020-08-25	Aarhus **controls**, batch

negative labels from different batches with validation using *only control* samples from different batches)	batch 2020-08-25	2021-02-10		2021-02-10

Supplementary Figure 17: Assessment of validation accuracy in 4 different melanoma deep learning model training set approaches.

a) Classifiers (*n*=6 per experiment) were trained with the same training and validation positive labels as used in our MRD-EDGE^{SNV} melanoma classifier (Supplementary Table 1). In our original training paradigm, negative labels for training and validation were drawn from NYGC controls, which were sequenced within the same batch as our training positive label (New York Genome Center, Illumina NovaSeq, Supplementary Table 5). In Experiment 1 and Experiment 2, negative labels for validation and training were drawn from samples sequenced within different batches on different platforms (Experiment 1: HiSeq controls, Illumina HiSeq, New York Genome Center) or different sequencing centers (Experiment 2: Aarhus controls, Illumina NovaSeq, Aarhus University). As a negative control, we trained the original melanoma positive label (batch 2020-08-25) against controls from a different batch (Aarhus controls) and substituted the validation positive label with non-cancer controls from the training positive label batch (batch 2020-08-25). We observed minimal discriminatory signal in this setting. Box plots represent median, lower and upper quartiles; whiskers correspond to 1.5 x interquartile range. **b)** Color table demonstrating sequencing batches used in classifier training and validation in panel **a)**. Each color denotes a distinct sequencing batch.

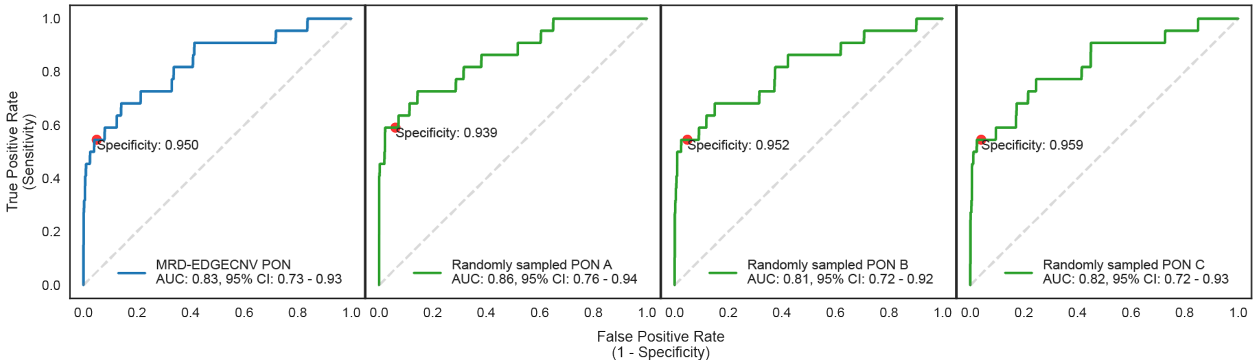

Supplementary Figure 18: Comparison of read depth panel of normal samples (PONs) in pretreatment, preoperative neoadjuvant non-small cell lung cancer

ROC analysis performed on read-depth Z-score values with 4 different PONs. Preoperative plasma samples (n=22) were used as the true label, and the patient-specific mutation profiles assessed against unmatched plasma samples (22 mutation profiles assessed across 20 control samples) was used as the false label (n=440 comparisons). Non-cancer plasma samples were randomly sampled to be in the PON or held-out from the PON. Performance in the original read-depth PON (blue) is highly generalizable compared to randomly sampled PONs in which controls were included in the PON vs. held-out of the PON. In each PON, control samples were held out of the PON and 65 samples were included in the PON. The 95.0% specificity threshold is marked in red in the randomly sampled PONs.

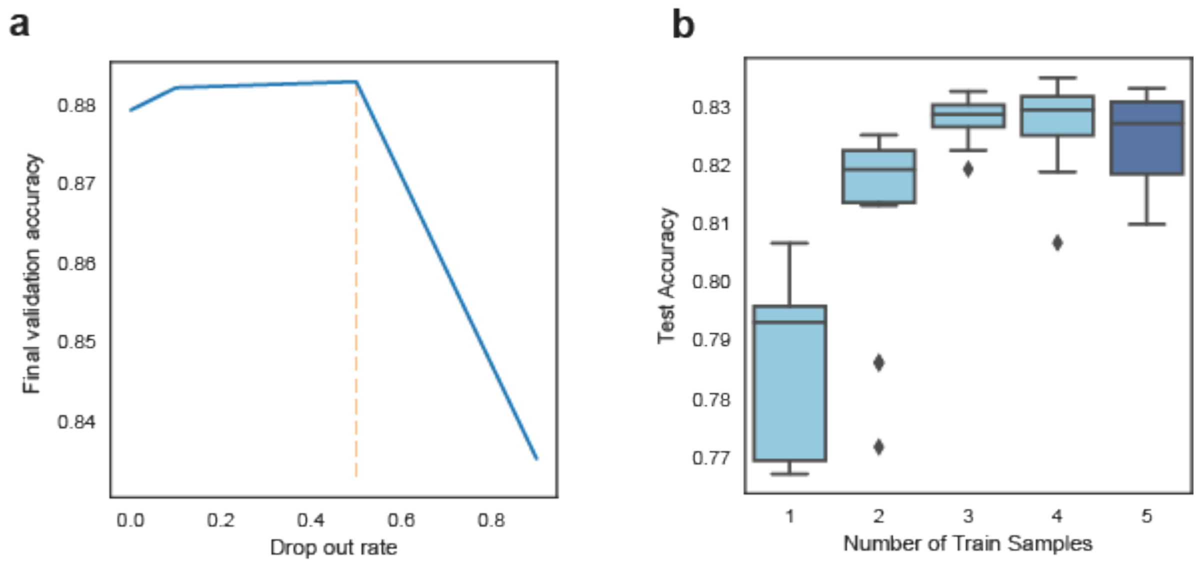

Supplementary Fig. 19: Sparsity and random sampling with replacement analyses for MRD-EDGE^{SNV}

a) Sparsity analysis for MRD-EDGE^{SNV} in melanoma. Melanoma models were trained at different dropout rates (0 to 0.9, blue line) and classification accuracy was evaluated in a held-out cutaneous melanoma validation set (Supplementary Table 1). Our chosen dropout rate of 0.5 (yellow dashed line) produced optimal accuracy in the held-out validation set. **B)** Random sampling with replacement for all possible combinations of training samples within the MRD-EDGE^{SNV} classifier. Models were trained on 1 to 5 high-burden colorectal samples against *n*=5 controls and performance was evaluated based on fragment classification accuracy in a test set held out from training (*n*=2 high-burden samples and *n*=2 non-cancer controls). The final MRD-EDGE^{SNV} classifier used the 5 high-burden samples with the most ctDNA fragments as the train set. Box plots represent median, lower and upper quartiles; whiskers correspond to 1.5 x interquartile range.

Flowchart Figures

Flowchart Fig. 1, Widman et al.

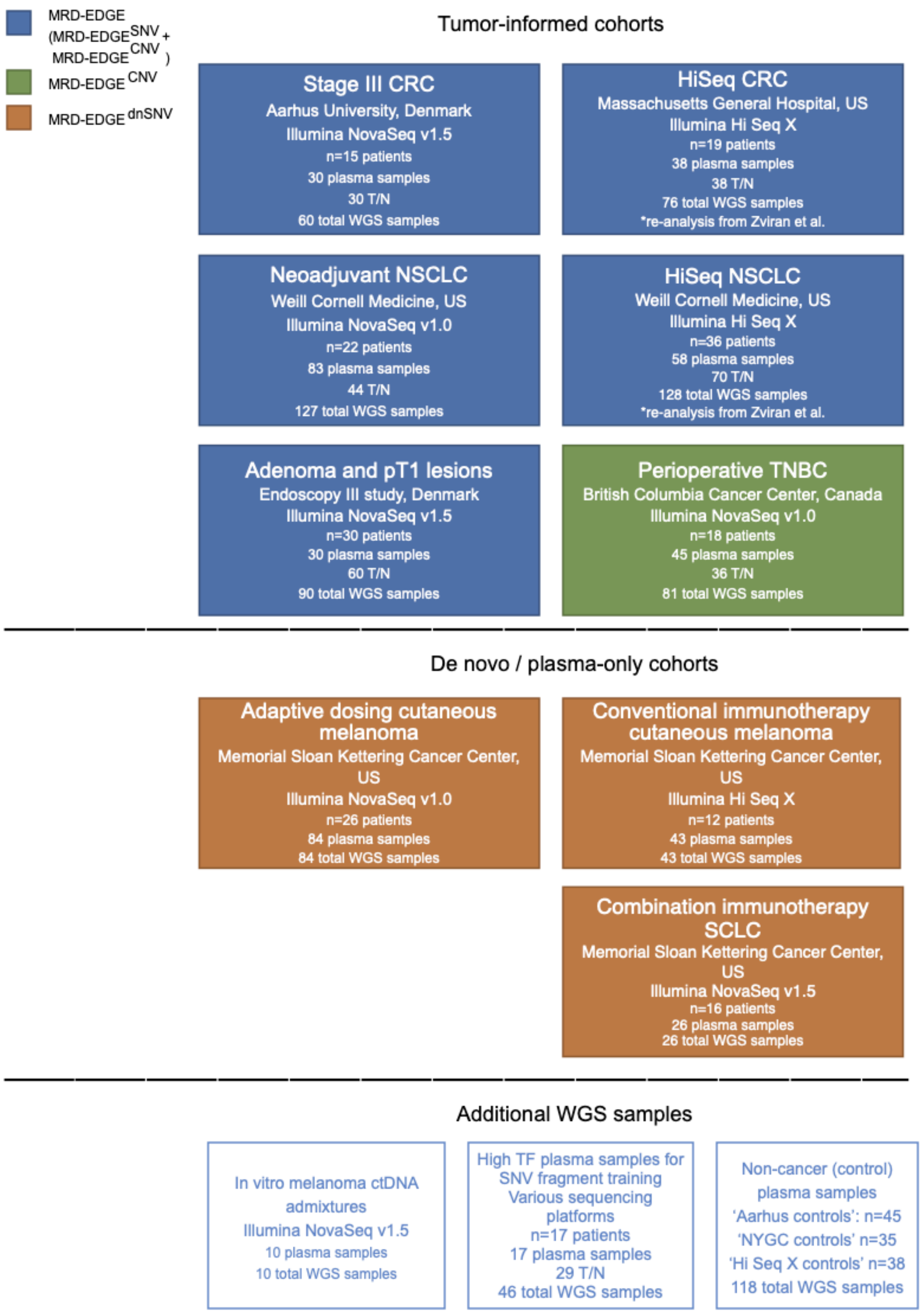

Flowchart Fig. 1: Overview of plasma WGS cohorts

Boxed description of clinical cohorts used throughout the study. Boxes indicate clinical context, sequencing preparation and number of WGS samples. Color indicates which MRD-EDGE workflow was applied (blue: tumor-informed MRD-EDGE, green: MRD-EDGE^{CNV}, orange: MRD-EDGE^{dnSNV}). T/N, tumor-normal pairs.

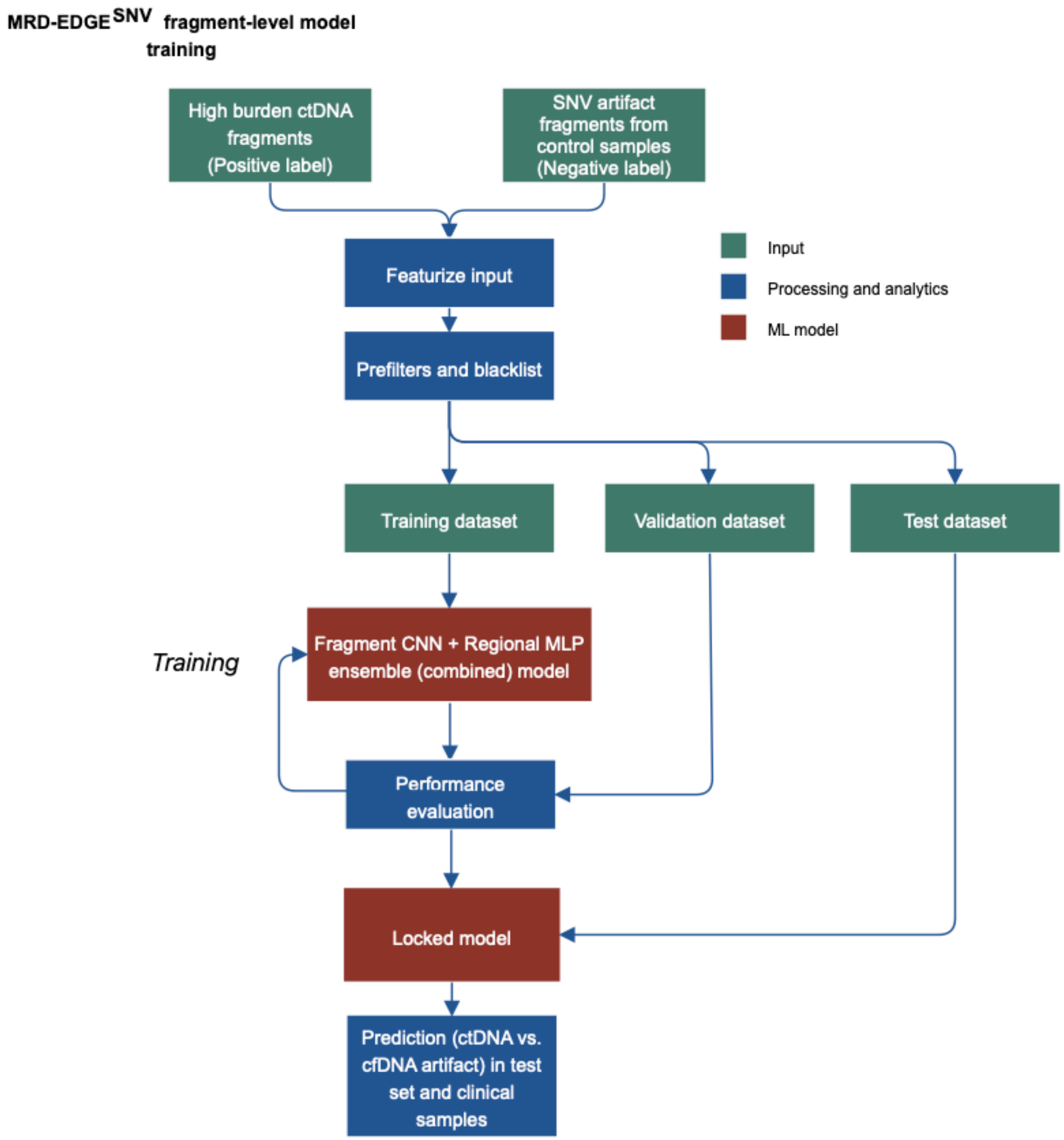

Flowchart Fig. 2: MRD-EDGE^{SNV} model training flowchart

Disease-specific ctDNA SNV fragments (positive label) are collected from patient plasma samples with high-burden metastatic disease. cfDNA SNV fragments (negative label) are sourced from

patient plasma samples from patients without cancer. Relevant features are extracted from genomic information and fragments are passed through our quality filters and blacklists (Methods). Data are partitioned into train, validation, and test datasets as described for each cancer type in Supplementary Table 1. The train dataset is used to train the MRD-EDGE^{SNV} ensemble of the Fragment CNN and Regional MLP (Fig. 1d, training is performed jointly as the ensemble evaluates the latent space outputs of the fragment and regional components) to classify cancer ctDNA vs. SNV artifact. Following training, the ensemble classifier undergoes performance evaluation in a held-out validation dataset. After optimization, the model is locked and undergoes performance evaluation in a held-out test set. The final result is a disease-specific (e.g., NSCLC, cutaneous melanoma, or CRC) SNV fragment classifier that is applied to clinical samples. Supplementary Table 1 provides train, validation, and test set performance metrics.

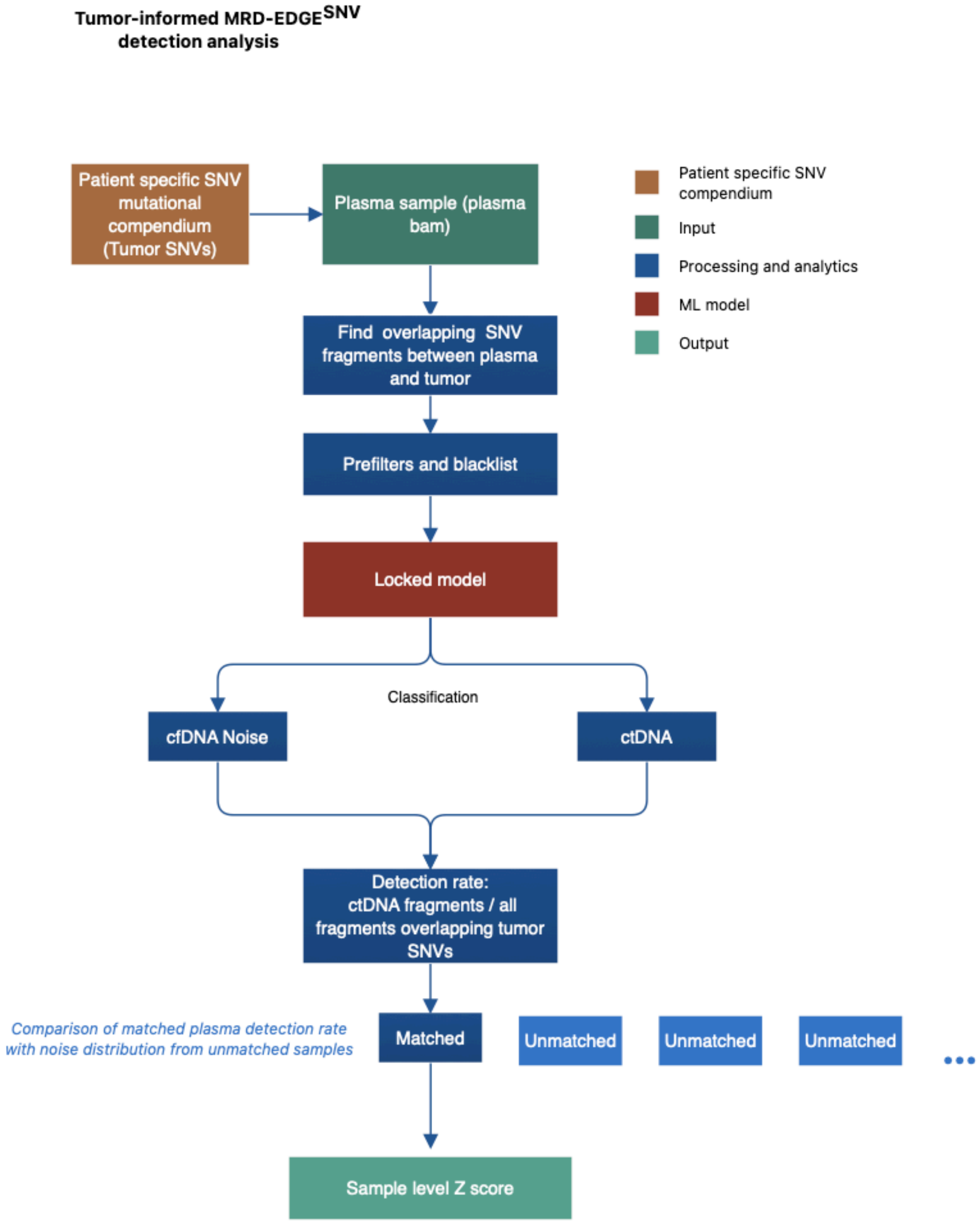

Flowchart Fig. 3: Flowchart for tumor-informed MRD-EDGE^{SNV} evaluation of plasma cfDNA

A patient-specific SNV profile captures SNVs in tumor tissue. Plasma at matching genetic loci is evaluated for matching SNV fragments, which are subsequently filtered by quality metrics and a recurrent SNV blacklist. A locked, disease-specific MRD-EDGE^{SNV} model is applied to post-filter SNV fragments which are classified as ctDNA (positive classification) or cfDNA artifact (negative classification). Detection rate is measured as the number of SNV fragments classified as ctDNA divided by the total number of fragments (SNV and non-SNV) found at all tumor SNV loci. At the sample level, the patient-specific SNV profile is applied to matched and unmatched plasma samples, as the latter form a detection rate noise distribution. Output is an MRD-EDGE^{SNV} Z score indicative of underlying ctDNA content.

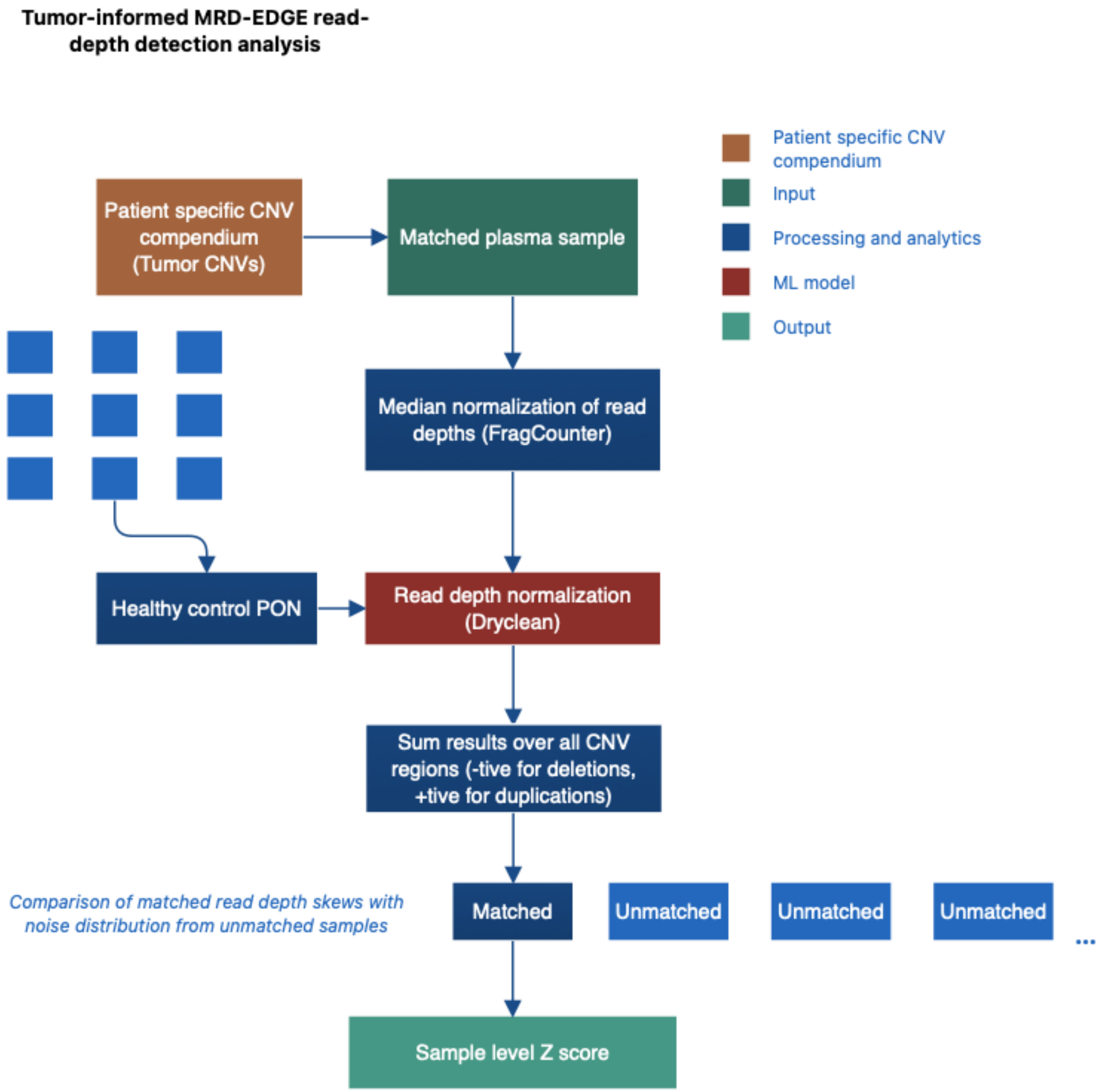

Flowchart Fig. 4: Flowchart illustration of read depth CNV classifier

A patient-specific CNV profile labels genomic windows as amplifications, deletions, and neutral regions in tumor tissue and is subsequently applied to a plasma sample to evaluate aneuploidy-associated read depth skews in cfDNA. Plasma read depths are median normalized and GC-corrected at each 10-kb window of the genome. Values are passed to dryclean, a machine-learning guided CNV denoising platform designed to detect read depth biases from a panel of

non-cancer plasma samples (panel of normal or PON). Foreground signal in excess of background PON signal is calculated for amplifications and deletions and aggregated at the sample level (Methods). Cumulative signal is compared to a noise distribution of foreground signal from unmatched (control) plasma samples, and the final sample-specific ctDNA tumor burden estimate is recorded as a Z score.

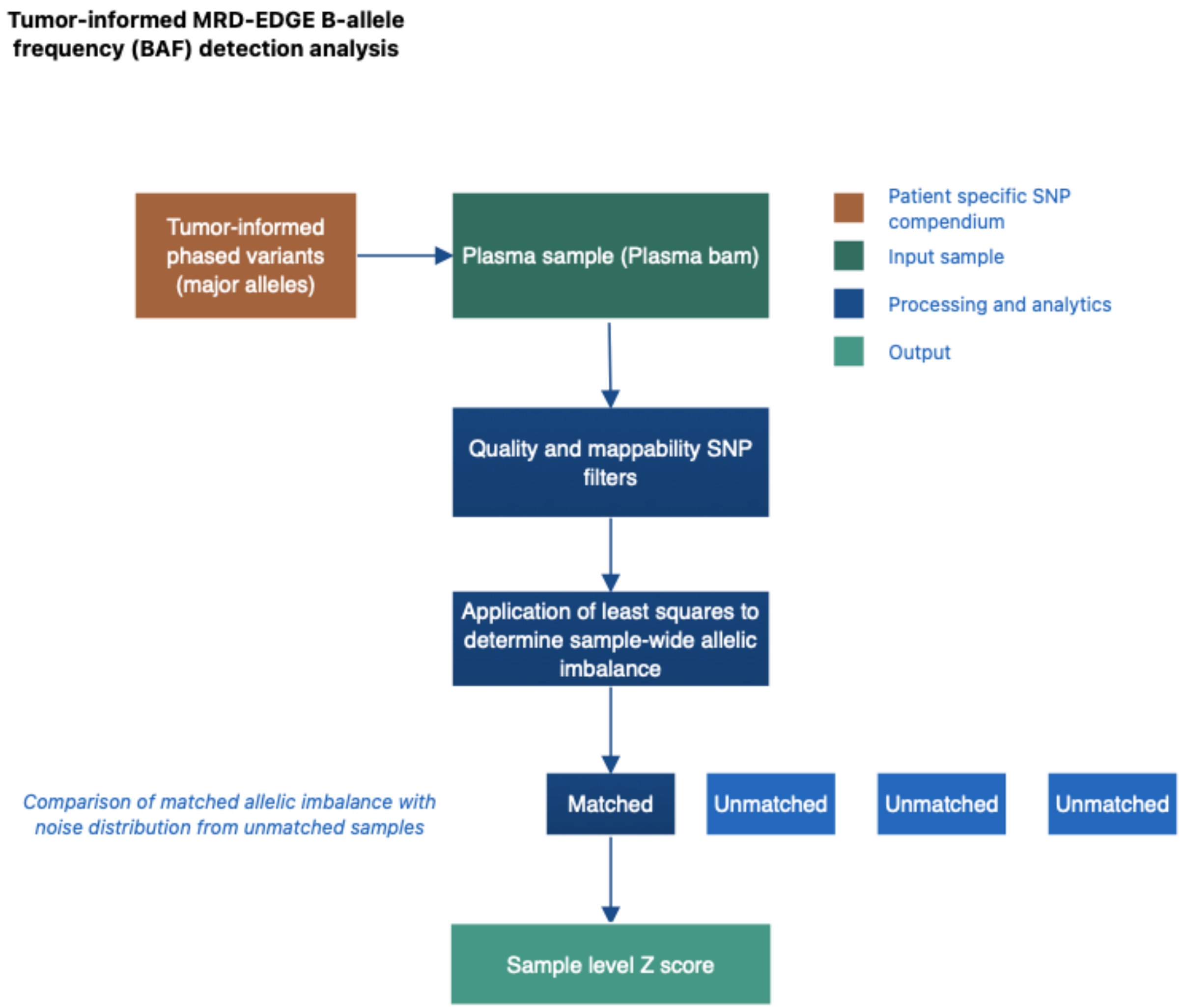

Flowchart Fig. 5: Flowchart illustration of B-allele frequency LOH classifier

A set of patient-specific single nucleotide polymorphisms (SNPs) and corresponding major alleles are sourced from loss of heterozygosity (LOH) regions in tumor tissue. Candidate plasma SNPs are subjected to quality filters and mappability correction (Methods). A least squares regression, based on the expected contribution of alleles per major allele, major and minor copy number state, and underlying plasma coverage, calculates estimated sample level ctDNA burden. The same approach is applied to unmatched (non-cancer) controls (Methods) to form a noise distribution, and the final result is a sample level BAF Z score indicative of plasma tumor burden.

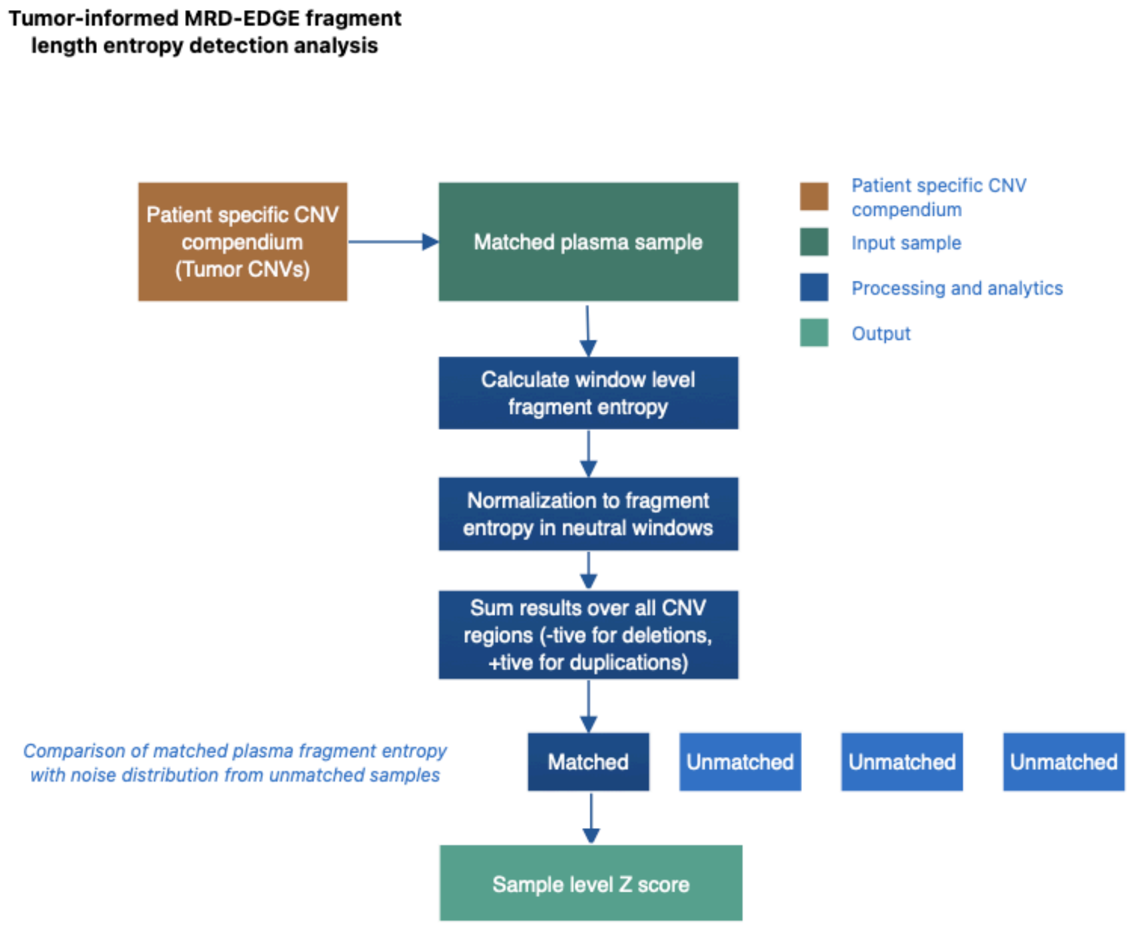

Flowchart Fig. 6: Flowchart illustration of fragment length entropy CNV classifier

A patient-specific CNV profile labels genomic windows as amplifications, deletions, and neutral regions in tumor tissue. In plasma, fragment length entropy is calculated for 100-kb non-overlapping genomic windows across the genome. These windows are normalized to entropy values in neutral regions using robust Z scores. Scores are aggregated across the genome according to segment direction, as windows in amplifications are expected to skew positive (more fragment length entropy than neutral regions due to greater ctDNA content in the cfDNA pool) while deletions are expected to skew negative (less fragment length entropy compared to neutral

regions due lesser ctDNA contribution to the plasma cfDNA pool). The aggregated entropy scores of amplifications and deletions form a sample level entropy score that is compared to a noise distribution of the same CNV regions applied to control samples. Output is a fragment length entropy Z score indicative of underlying ctDNA content.

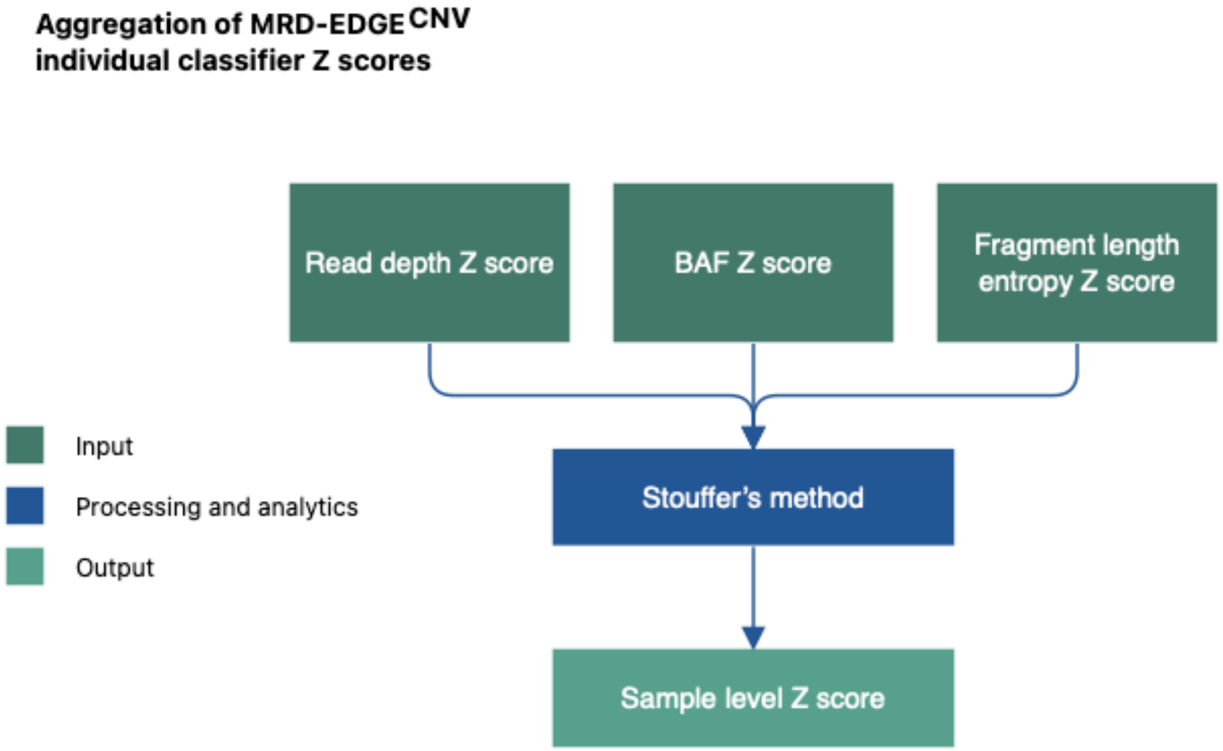

Flowchart Figure 7: Flowchart for integrating information from 3 CNV classifiers to produce sample-level MRD-EDGE^{CNV} Z score

Individual read depth, BAF, and fragment length entropy Z scores are summed via Stouffer's method to form Z scores for cancer plasma samples (signal) and control plasma samples (noise distribution).

Flowchart Fig. 8, Widman et al.

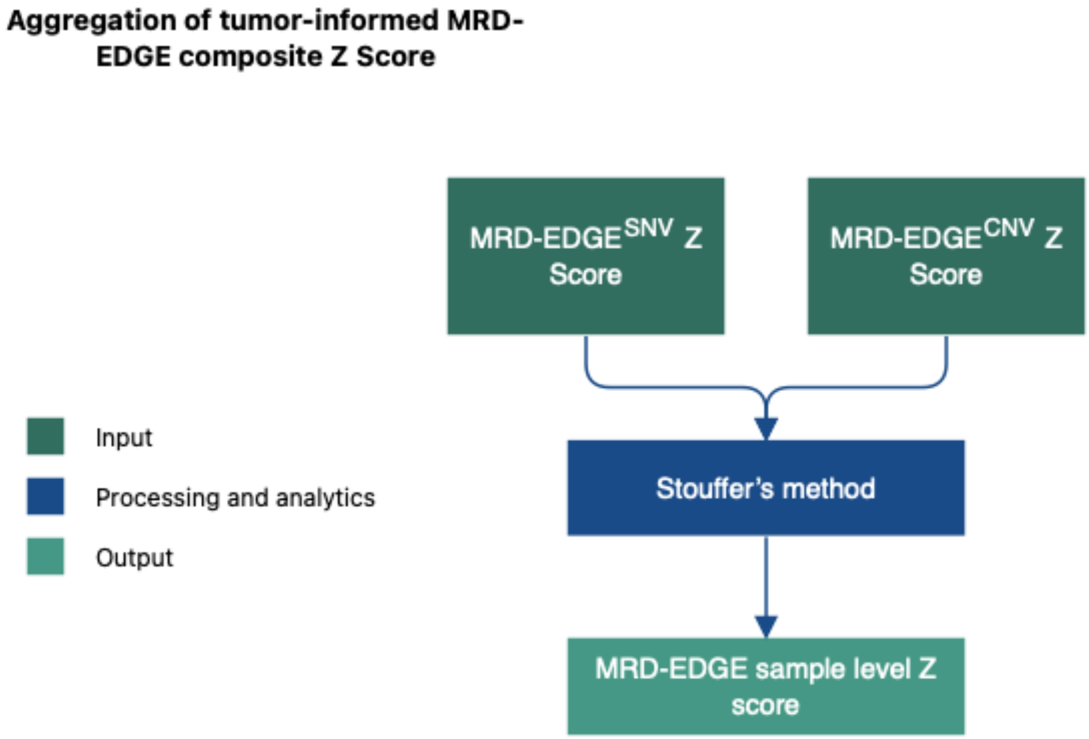

Flowchart Figure 8: Flowchart for integrating information from MRD-EDGE^{SNV} and MRD-EDGE^{CNV} Z scores to produce sample-level MRD-EDGE Z score

MRD-EDGE^{SNV} and MRD-EDGE^{CNV} Z scores are summed via Stouffer's method to form Z scores for cancer plasma samples (signal) and control plasma samples (noise distribution).

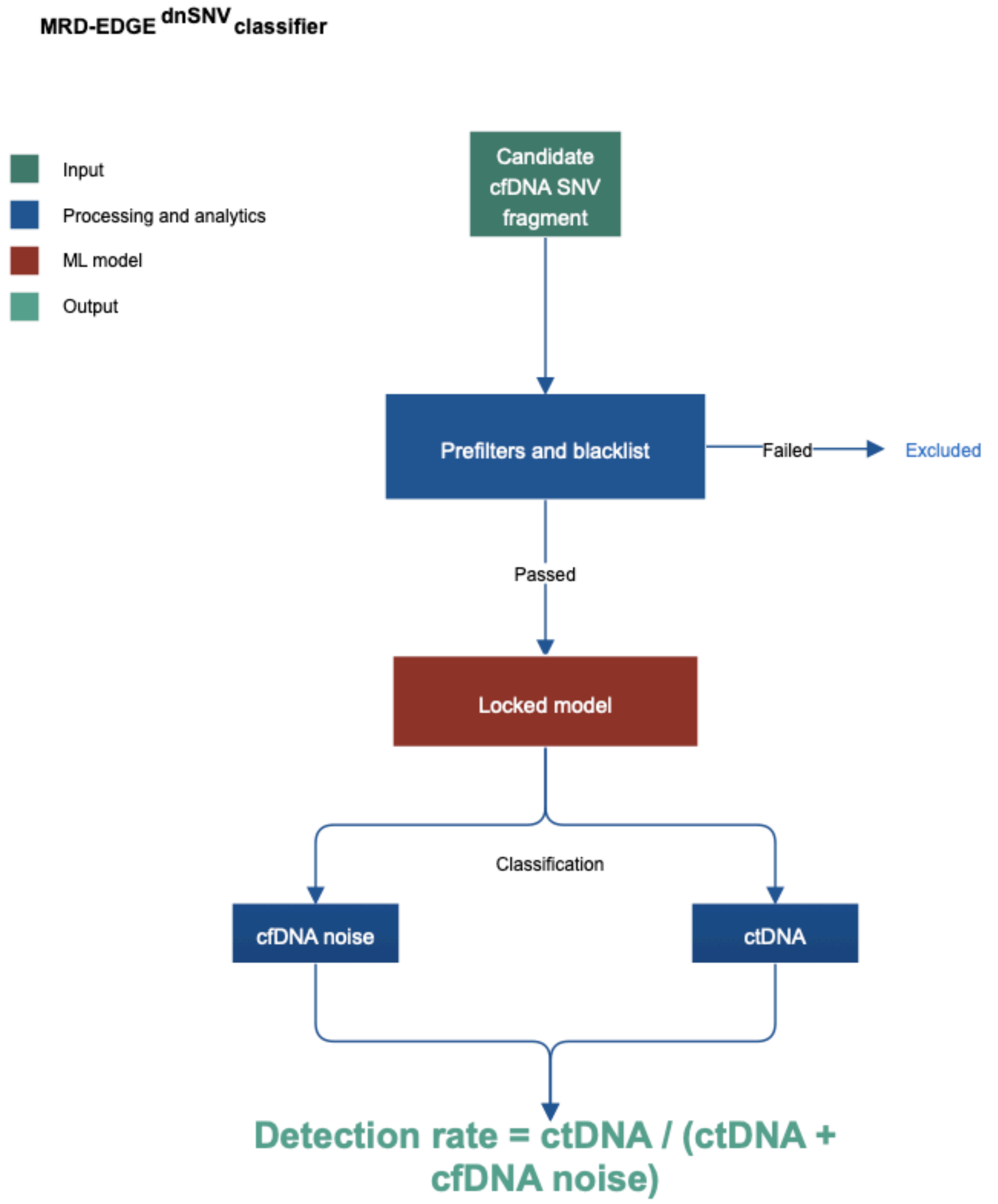

Flowchart Fig. 9: Flowchart for MRD-EDGE^{dnSNV} evaluation of plasma cfDNA

All cfDNA fragments with SNVs are passed through quality filters and recurrent artifact blacklists. A trained, disease-specific MRD-EDGE^{dnSNV} deep learning classifier evaluates post-filter fragments and classifies fragments as ctDNA or noise. Detection rate is measured as the number of SNV fragments classified as ctDNA divided by the number of SNV fragments evaluated and can be used to track changes in plasma TF over time and in response to therapy.

References

85. Lundberg, S. & Lee, S.-I. A Unified Approach to Interpreting Model Predictions. *arXiv [cs.AI]* (2017).

86. Guraya, S. Y. Pattern, Stage, and Time of Recurrent Colorectal Cancer After Curative Surgery. *Clin. Colorectal Cancer* **18**, e223–e228 (2019).

87. Srivastava, N., Hinton, G., Krizhevsky, A., Sutskever, I. & Salakhutdinov, R. Dropout: A Simple Way to Prevent Neural Networks from Overfitting. *J. Mach. Learn. Res.* **15**, 1929–1958 (2014).
